# IL-2 based cancer immunotherapies: an evolving paradigm

**DOI:** 10.3389/fimmu.2024.1433989

**Published:** 2024-07-24

**Authors:** Sushama Rokade, Anita Mehta Damani, Martin Oft, Jan Emmerich

**Affiliations:** Development Department, Synthekine, Menlo Park, CA, United States

**Keywords:** IL-2, cancer immunotherapy, IL-2 receptors, IL-2 toxicities, IL-2 variants, combination therapy, oncolytic viruses

## Abstract

Discovered over 4 decades ago in the supernatants of activated T cells, interleukin-2 (IL-2) is a potent pleiotropic cytokine involved in the regulation of immune responses. It is required for effector T cell expansion and differentiation as well as for peripheral tolerance induced by regulatory T cells. High-dose IL-2 treatment was the first FDA-approved immunotherapy for renal cell carcinoma and melanoma, achieving single agent complete and durable responses, albeit only in a small proportion of patients. The therapeutic potential of wild type IL-2 is clinically limited by its short half-life and severe vascular toxicity. Moreover, the activation of regulatory T cells and the terminal differentiation of effector T cells on IL-2 pose additional restrictions. To overcome the toxicity of IL-2 in order to realize its full potential for patients, several novel engineering strategies are being developed and IL-2 based immunotherapy for cancer has emerged as a burgeoning field of clinical and experimental research. In addition, combination of IL-2 with PD-1/L1 pathway blockade shows vastly improved anti-tumor efficacy over either monotherapy in preclinical tumor models. In this review we discuss the biological characteristics of IL-2 and its receptors, as well as its efficacy and treatment limiting toxicities in cancer patients. We also explore the efforts aimed at developing novel and safer IL-2 therapies to harness the full therapeutic potential of this cytokine.

## 1 Introduction

Interleukin-2 (IL-2), was first identified in 1976 as a T cell growth factor in the supernatants of activated T cells (1). The availability of purified and recombinant IL-2 allowed long term culture of activated T cells and natural killer (NK) cells enabling further investigation of their activation, function, differentiation, and regulatory pathways (2–5). IL-2 is a potent pleiotropic cytokine involved in exerting dual functions of driving effector T (Teff) cell expansion and differentiation as well as mediating peripheral tolerance by inducing regulatory T cells (Tregs) (6–12).

**Figure 1:**
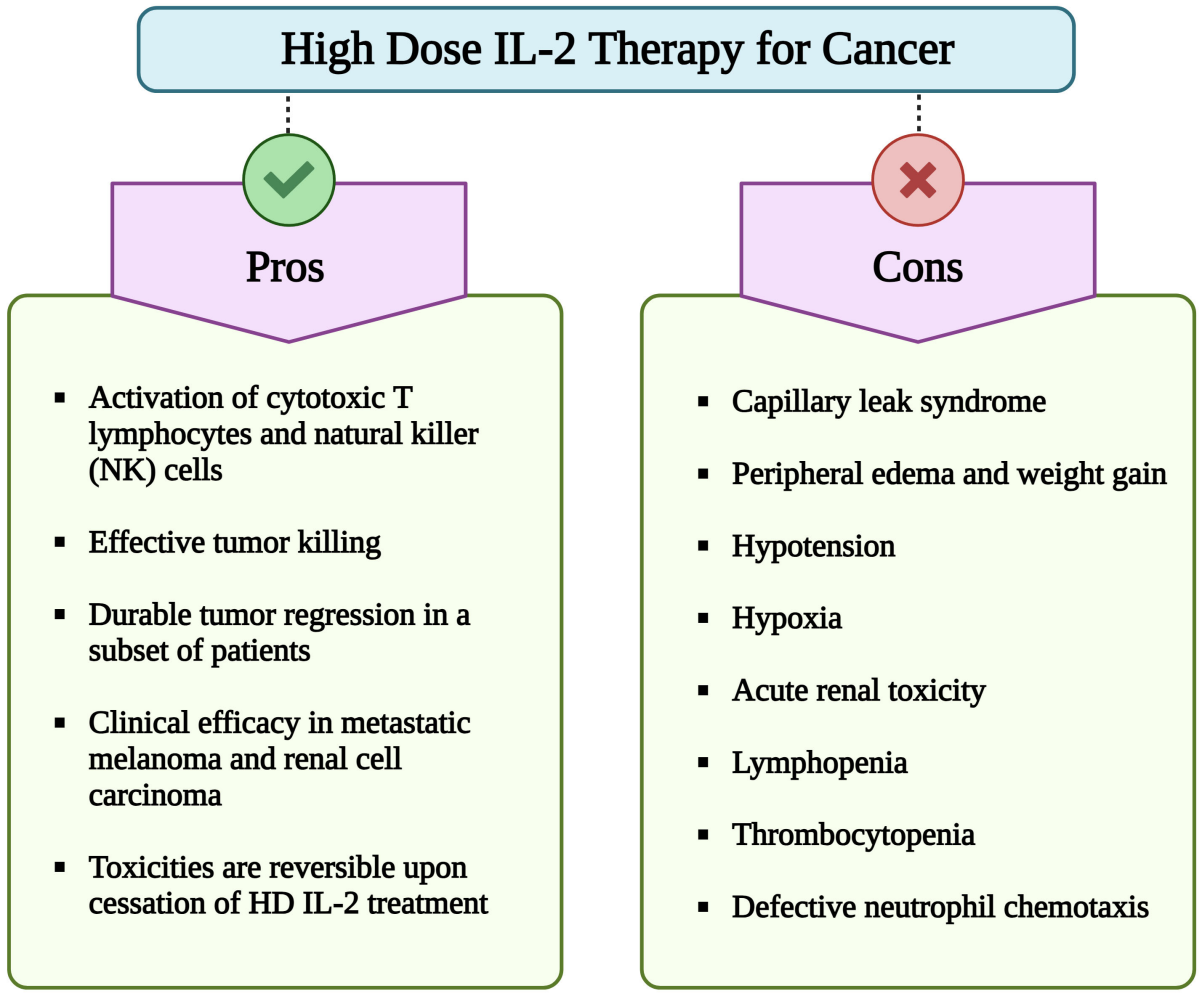
Pros and cons of high dose (HD) interleukin-2 (IL-2) therapy for cancer. Although HD IL-2 therapy for cancer confers advantages such as activation of cytotoxic T cells and NK cells, sustained tumor regression in subset of patients with metastatic melanoma and renal cell carcinoma, its extensive utilization is hindered by numerous adverse effects. Important limitations include a short half-life of less than 30 min, necessitating repeated administration of multiple doses of IL-2. Additionally, major side effects include capillary leak syndrome, hypotension, hypoxia, lymphopenia, thrombocytopenia, and impaired neutrophil chemotaxis. (Image created with Biorender.com).

Due to its capacity to activate and expand cytotoxic effector cells, IL-2 immunotherapy has been examined in numerous clinical trials. In 1992, the US Food and Drug Administration (US-FDA) approved high-dose (HD) IL-2 (aldesleukin) for the treatment of renal cell carcinoma (RCC). This approval was based on 7 phase II clinical trials with 255 metastatic RCC (mRCC) patients treated with HD IL-2 (600,000–720,000 IU/kg, corresponding to 37-44 µg/kg) every 8 hours for a maximum of 14 doses per cycle. HD IL-2 treatment resulted in a 14% overall objective response rate (ORR) with 5% complete responses (CRs) and 9% partial responses (PRs). The median response duration of partial responders was 19 months (13). Further in 1998, HD IL-2 therapy was also approved for the treatment of metastatic melanoma (MM) based on a 16% ORR with 6% CRs, 10% PRs, and 5.9 months median response duration for PRs among 270 patients with MM from 8 clinical trials (14, 15). Importantly, a large number of the CRs induced by HD IL-2 are durable and lead to long-term survival with median duration of overall survival of over 10 years reported (16–19). However, the widespread use of HD IL-2 therapy has been limited due to its significant toxic side effects in responding and non-responding patients, including capillary leak syndrome (CLS) (**Figure 1**). The elucidation of the crystal structure of IL-2 in complex with its trimeric receptor (20) together with data showing that IL-2 synergizes with programmed death-1 (PD-1) pathway blockade in reinvigorating exhausted T cells (21), has reignited the interest to develop improved IL-2 based therapies. In this review efforts undertaken by multiple research groups and pharma companies to develop novel and safer IL-2 therapeutics will be discussed.

## 2 Biology of IL-2 and IL-2 receptors

IL-2 is a 15.5 kDa glycoprotein, consisting of a four α-helix bundle. It plays an important regulatory role during both resting and activated states of the immune system (22, 23). T cells are the dominant source of IL-2 (24) and IL-2 is rapidly and transiently produced upon activation of naïve CD4+ and CD8+ T cells through engagement of T cell receptor (TCR) and costimulatory signals. Interestingly, induction of both IL-2 and the IL-2R *in vivo* corresponds to the intensity of the antigenic stimulus (25). This temporal nature of IL-2 production is determined by TCR signals inducing transcriptional activation and costimulatory signals stabilizing IL-2 transcripts, followed by inhibition of IL-2 gene transcription and rapid degradation of IL-2 mRNA (26).

**Figure 2:**
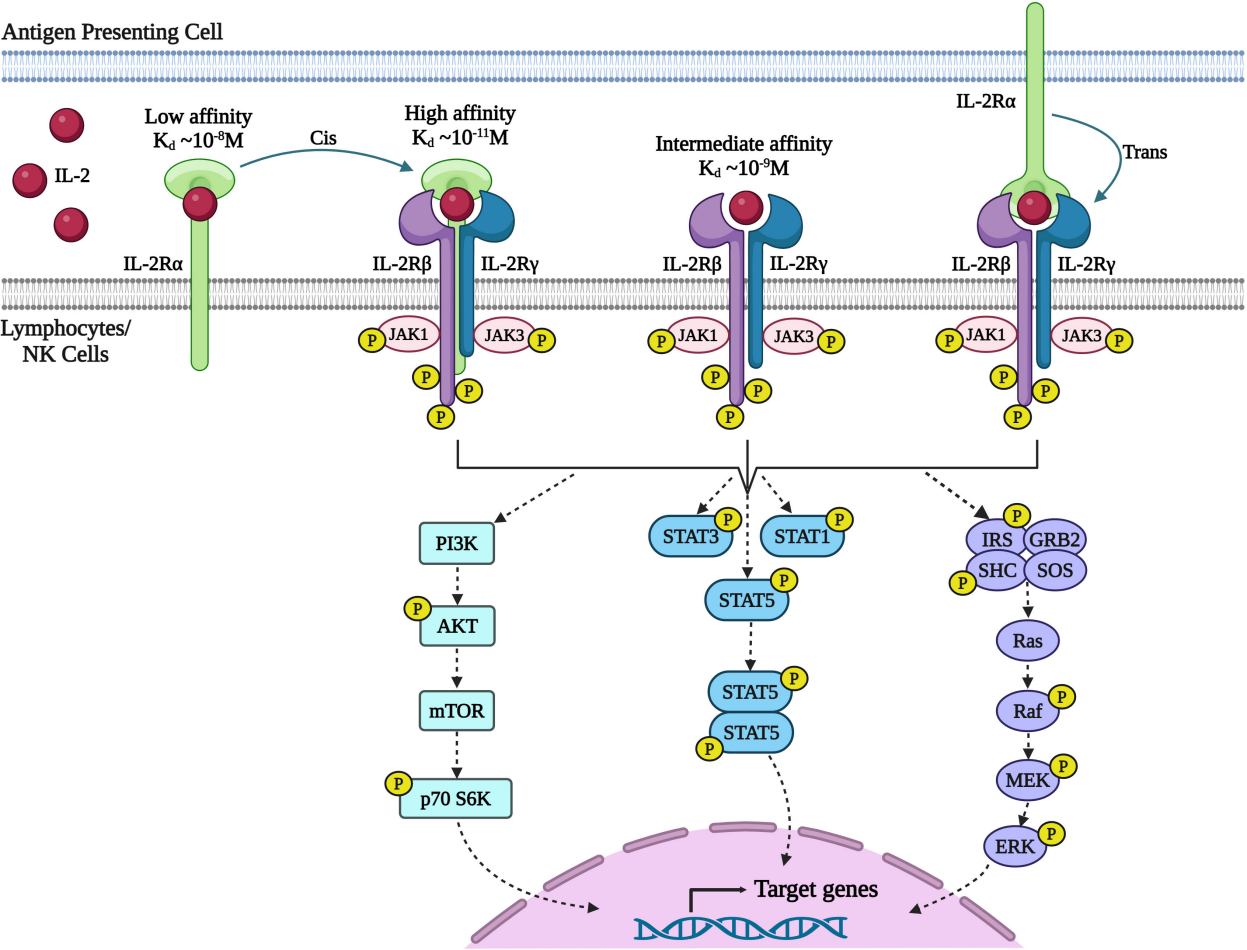
Signaling through IL-2 receptor (IL-2R) pathway. IL-2 exerts its effects on lymphocytes by binding to the multimeric IL-2R. The receptor complex consists of three subunits: IL-2Rα (CD25), IL-2Rβ (CD122), and IL-2Rγ (also known as the common γ-chain or γc/CD132). IL-2Rα is a low-affinity receptor that does not initiate a signal. The IL-2Rβ and IL-2Rγc chains together form a dimeric receptor with intermediate affinity. When combined with IL-2Rβ and IL-2Rγc, the IL-2Rα chain forms a high-affinity receptor with a 100-fold higher affinity for IL-2. Additionally, IL-2 bound to IL-2Rα on one cell, such as dendritic cells, can activate other cells expressing IL-2Rβ and IL-2Rγc through trans-presentation (27). Upon receptor engagement, IL-2 initiates three major signaling pathways (1): Janus kinase (JAK)-signal transducer and activator of transcription (STAT) (2), mitogen-activated protein kinase (MAPK), and (3) phosphoinositide 3-kinase (PI3-K)-AKT. However, recent data has questioned the direct control of the PI3K-AKT pathway by IL-2. The cytoplasmic domains of IL-2Rβ and IL-2Rγc chains heterodimerize, initiating signal transduction by phosphorylating tyrosine kinases that activates JAK-STAT pathway and Ras-MAP kinase pathway leading to activation of target genes necessary for effector T cell function, proliferation, and differentiation. (Image created with Biorender.com).

IL-2 mediates its action on lymphocytes via binding to the multimeric IL-2R resulting in activation of signal transduction pathways that lead to lymphocyte proliferation, survival, and function (**Figure 2**). The IL-2R is formed by different combinations of three subunits: IL-2Rα (CD25), IL-2Rβ (CD122), and IL-2Rγ, also known as the common γ-chain (γc/CD132). IL-2Rβ is also a part of the IL-15 receptor, while IL-2Rγc is the shared receptor chain of the receptors for IL-4, IL-7, IL-9, IL-15, and IL-21. IL-2Rα is a low affinity IL-2R that lacks intracellular signaling domains and interaction of IL-2 with CD25 alone does not induce a signal (22). The β and γc chains together form a dimeric intermediate affinity receptor responsible for signaling that is highly expressed on NK cells and to a lesser degree on memory CD4+ and CD8+ T cells. The α-chain is expressed constitutively on Tregs, type-2 innate lymphoid cells (ILC2), and transiently highly upregulated upon antigen mediated activation on CD4+ and CD8+ T cells (22, 28). The IL-2R α-chain together with the β and γc chains, constitutes a high affinity receptor with 100-fold higher affinity for IL-2 than the intermediate IL-2R. Therefore, high amounts of IL-2 are required for the expansion of resting lymphocytes *in vivo* whereas Tregs and activated T cells require only low doses of IL-2 (20, 29).

Upon receptor binding, IL-2 activates three major signaling pathways (1): Janus kinase (JAK)–signal transducer and activator of transcription (STAT) (2); mitogen-activated protein kinase (MAPK) pathway; and (3) phosphoinositide 3- kinase (PI3K)–AKT (11). Heterodimerization of the cytoplasmic domains of IL-2Rβ and γc initiates signal transduction through activation of tyrosine kinases JAK1 and JAK3 respectively (30). In turn JAK1 and JAK3 phosphorylate three key tyrosine residues within the cytoplasmic tail of IL-2Rβ. Phosphorylation of Y392 and Y510 in humans (Y395 and Y498 in mice) leads predominantly to the recruitment of STAT5A and STAT5B and to a lesser degree STAT1 and STAT3 to IL-2Rβ (31). These STAT proteins then undergo phosphorylation and dimerization, and translocate to the nucleus, where they bring about activation of target genes essential for effector T cell function, proliferation and differentiation (31–33). Phosphorylation of Y338 in humans (Y341 in mice) promotes recruitment of the SHC adaptor protein, leading to the activation of Ras-MAP kinase associated with enhanced cellular proliferation. While the activation of the STAT5 and RAS-MAPK pathways are the best studied signaling pathways downstream of the IL-2R, IL-2-JAK activation modulates a wide range of signaling pathways, altering the phosphorylation status of hundreds of proteins and thereby controlling a wide range of cellular functions such as transcription, translation, vesicle transport and metabolism (12, 34). Phosphoproteome analysis also suggested that IL-2 rather than directly activating the PI3K mTOR pathway, integrates with the PI3K signaling preactivated by SRC family kinases, FYN and LCK in T cells to increase the activity of mammalian target of rapamycin complex (mTOR) pathway (12).

Association of IL-2 with IL-2R causes internalization of the quaternary complex, where IL-2, IL-2Rβ, and IL-2Rγc are degraded in vesicles, while IL-2Rα is recycled to the cell surface via endosomes (35). For sustained IL-2R signaling, persistent stimulation with IL-2 is essential. Autocrine secreted IL-2 must be captured at the cell surface to minimize its loss by diffusion away from the cell. The high affinity of IL-2Rα for IL-2 and the large excess of IL-2Rα over IL-2Rβ and IL-2Rγc on activated T cells facilitate this capture and delivery to IL-2Rβ and the IL-2Rβγc complex through two-dimensional cell surface diffusion (35). However, upon IL-2 binding and signaling, IL-2Rα is cleaved of the cell surface by a disintegrin and metalloproteinase 17 (ADAM17), and this soluble IL-2Rα can potentially act as a decoy receptor (36).

The activation of transcription factor B lymphocyte-induced maturation protein 1 (Blimp1), which is triggered by IL-2 signaling, acts as a negative feedback loop by suppressing the production of IL-2 (37). T cells that are exposed to extensive antigen stimulation and strong IL-2R signaling express high Blimp-1 and become terminally differentiated short-lived effector cells. On the other hand, T cells that receive weak IL-2 signaling tend to develop into a long-lived memory cell phenotype driven by the transcription factor Bcl-6 (38).

IL-2 signaling in Tregs is distinct from T effector cells due to high expression of PTEN (phosphatase and tensin homolog) protein which essentially results in failure to activate the PI3K-AKT pathway. Despite the high-affinity IL-2R expression in Tregs, increased PTEN expression regulates the anergic response of Tregs to IL-2 *in vitro* and Treg homeostasis *in vivo* (39, 40).

## 3 Limitations of HD IL-2 therapy

Despite encouraging results with HD IL-2 therapy for MM and mRCC, the widespread use of HD IL-2 therapy has been hampered due to its significant toxicity, necessitating hospitalization at expert centers and limiting its use to patients with intact or minimally impaired functional status (16) (**Figure 1**, **Table 1**). While lower IL-2 doses were clinically evaluated in an attempt to reduce toxicity, these were shown to be less efficacious than HD IL-2 (54, 55). Therefore, the guideline for HD IL-2 therapy is to administer the maximum number of doses possible without putting the patient at unacceptable risk for severe, irreversible toxicities (16). IL-2 shows bi-phasic clearance with a distribution half-life of 7-13 min and a clearance half-life of 60-86 min (56, 57). Due to its short half-life, HD IL-2 is administered intravenously (i. v.) at a dose of 600,000 - 720,000 IU/kg every 8 hours to a maximum of 14-15 doses per cycle. However, as side effects are generally cumulative, worsening and becoming refractory to management with successive doses, most patients don’t receive a full cycle (16). The most common and complicating toxicities are severe hypotension requiring fluid and vasopressor support and CLS leading to dose-limiting pulmonary edema, pulmonary infiltrates, renal failure, transaminitis, diarrhea, and altered mental status as well as progressive, substantial weight gain (58, 59). Thrombocytopenia and lymphopenia are also commonly observed with HD IL-2 treatment and coincide with CLS. Within hours of administration of the first dose of HD IL-2, lymphocytes are strongly decreased or even disappear from the circulation, most likely due to margination and diapedesis. However, upon cessation of IL-2 administration these cell counts rapidly return to normal and often rebound to 2-5 times their baseline levels (16). Hypotension and CLS are considered mechanism related toxicities of HD IL-2 and both toxicities remain largely refractory to satisfying management leading to a premature stop of IL-2 treatment. Engineering of IL-2 to avoid these toxicities is therefore considered one of the main goals for the development of new IL-2 based therapies. Several mechanisms have been proposed for the induction of CLS. A prominent hypothesis is that IL-2 directly activates IL-2Rαβγc expressing endothelial cells, resulting in the disruption of structural integrity of lung vasculature and the blood-brain barrier (BBB) (60–63) and leading to pulmonary and brain edema. It has also been proposed that the activation of IL-2Rαβγc expressing ILC2 by IL-2 leads to the secretion of IL-5 which in turn activates eosinophils that produce inflammatory cytokines that induce CLS (64, 65). Based on these data and to avoid Treg stimulation, CD25 avoiding/non-alpha IL-2 variants (IL-2v) were developed, such as bempegaldesleukin, SAR444245 and Nemvaleukin. However, challenging these mechanisms of CLS induction, IL-2 receptors are generally believed to be largely restricted to lymphoid cells (11), and, more importantly, non-alpha IL-2s remain to induce CLS associated pathologies including hypotension. In addition, capillary leak occurs starting with the first treatment cycle of HD IL-2, while the induction of eosinophils is observed in subsequent cycles (16). An alternative explanation for the induction of CLS involves the direct, systemic activation of IL-2Rβγc expressing NK cells by IL-2. As a consequence, NK cells produce inflammatory cytokines and chemokines, recruiting and activating neutrophils that in turn mediate the activation of endothelial cells. Depletion or genetic deficiency of NK cells renders mice resistant to develop CLS in response to IL-2 (66–68) or the combination of IL-12 and IL-2 (69). Based on these data, IL-2v selectively stimulating the trimeric receptor and relying on IL-2Rα binding, such as BAY 50-4798 (70, 71) and STK-012 (72) were developed. Lastly, the binding of a toxin motif in IL-2 to endothelial cells has been proposed to damage vascular endothelial cells leading to CLS (73). The anti-tumor activity of IL-2 is generally accepted to be mediated by tumor-specific CD8+ T cells (67, 74–76).

**Table 1:** Side effects of select IL-2 drug candidates in clinical development.

Molecule	HD IL-2 (Aldesleukin)	PEG-IL2	Darleukin	hu14.18-IL2	WTX124	Bempeg	SAR444245	Nemvaleukin	MDNA11	Xilio	NHS-IL2(D20T)	STK-012
Bias	unbiased	unbiased	unbiased/targeted	unbiased/targeted	unbiased/conditional	non-α	non-α	non-α	non-α	non-α/conditional	α-biased	α-biased
Mode of Therapy	monotherapy	monotherapy	monotherapy	monotherapy	monotherapy, first four dose levels	monotherapy	monotherapy	monotherapy	monotherapy	monotherapy, first seven dose levels up to 4 mg/kg	monotherapy	monotherapy
Clinical Trial Phase	II	I	II	II (pediatric)	I	I	I	II	I	I	I	I
Reference	[Package Insert^#^]	(41)	(42)	(43)	(44)	(45, 46)	(47, 48)	(49)	(50) (% estimated based on bar graph)	(51)	(52)	(53)
[n]	525	35	12	38	16	28	58/20(48)	74	30	62	39	47
TRAE	≥10%, any grade	grade 3-4	any grade	grade 3 &4	any grade	≥10%		≥10%	≥10%	≥10%	≥20%	≥10%
Capillary Leak Syndrome and related adverse events												
Capillary Leak Syndrome	+	+	+	32%	0%			0%		0%	0%	0%
Hypotension	71%	46%		16%		57%	20%(48)	34%	23%		31%	
Fatigue / Malaise / Asthenia	27%				38%	71%	26%	23%		19%	23%	26%
Pyrexia (Fever)	29%	34%	Constitutional symptoms 100%	40%	6%		48%	65%	43%	18%	36%	
Flu-like symptoms					13%	68%	45%		50%			
Cytokine Release Syndrome					0%	11%	16%					
AST (SGOT)	23%			24%			40%(48)	29%	23%			
ALT				21%			30%(48)	29%	23%			
Lymphopenia	16%			40%			24%	7%		15%	36%	
Skin & subcutaneous tissue		9%	Dermatology 100%									
Pruritis	24%				13%	64%			13%			15%
Rash	42%			5%	13%	50%			9%		46%	40%
Gastrointestinal			Gastrointestinal 83%									
Diarrhea	67%					11%		5%	20%			20%
Nausea	35%				19%	25%	35%	38%	34%			23%
Vomiting	50%					14%	29%	19%	20%			23%
Nausea and vomiting	19%	9%										
Eosinophilia	+				+	+	–		–		+	

#Copy of package insert provided with **Supplementary Files**.

In addition to its toxicity, IL-2 administration also leads to the expansion of Tregs in the blood of treated cancer patients (77), which can potentially limit the anti-tumoral activity of IL-2 treatment (78). In preclinical models, depletion of Tregs has been shown to improve anti-tumor immune responses. However, Treg depletion has not been successful yet in the clinic, likely due to use of CD25 directed antibodies that not only target Tregs but also activated effector T cells such as denileukin diftitox (Ontak), an IL-2-diphtheria toxin fusion protein (79–81). Conversely, Treg depletion in mice with a CD25 targeting antibody has been shown to increase IL-2 induced CLS (82), suggesting that systemic expansion of Tregs in response to IL-2 treatment might be beneficial in the control of IL-2 toxicities. Moreover, IL-2 treatment can lead to the induction of potent effector T cell responses despite the observed expansion and activation of Tregs (21, 78, 83).

Importantly, recent data suggest that CD25 expression on CD8+ T cells is required for native IL-2 to induce effective anti-viral and anti-tumor CD8+ T cell responses (84–86). In chronic viral infection, IL-2 was shown to lead to the differentiation of stem-like PD1+TCF1+CD8+ T cells into “better effector” CD8+ T cells that were epigenetically and transcriptionally similar to effector cells generated during acute infections (84). However, IL-2 monotherapy had little impact on the viral load during chronic LCMV infection due to PD-L1 expression in the target tissues. Consequently, combination of IL-2 with PD-1 blockade showed strong synergism on viral control. Importantly, blocking IL-2Rα with an antibody or using a mutated version of IL-2 (IL-2v) that does not bind to IL-2Rα abrogated this synergistic effect seen after anti-PD-1 plus IL-2 combination therapy. Neither anti-PD-1 or IL-2v monotherapy nor the combination of the two induced the transcriptional changes seen with wild-type (WT) IL-2 (84). However, targeting the IL-2v to effector memory T cells via fusion to an anti-PD1 antibody, overcame the need for IL-2Rα binding and this anti-PD1-IL-2v potently increased the anti-viral T cell response (87).

In line with these findings, a recent study has shown that Fc-fusion proteins of both WT IL-2 and an IL2Rα-biased IL-2v (N88D) more effectively expand tumor-antigen specific CD8+ T cells (TSTs) and induce more potent anti-tumor responses than a Fc fusion of an IL2Rβγc-biased IL-2v while also exhibiting better safety. As expected, the IL-2Rα-biased IL-2v decreased the CD8/Treg ratio in the blood, but surprisingly increased the CD8/Treg ratio in the tumor. Interestingly, only the combination of anti-PD-1 and WT IL-2, but not IL-2 alone increased the CD8/Treg ratio in the LCMV model (84). The TSTs co-expressed CD25 and PD-1 and the IL-2Rα-biased but not the IL-2Rβγc-biased IL-2v synergized with PD-1 pathway blockade. Moreover, autocrine IL-2–IL-2Rα signaling was found to be crucial for the efficacy of anti-PD-1 therapy as blocking IL-2Rα compromised anti-PD-1 efficacy. IL-2Rα, alongside PD-1, emerged as a more precise predictor of TST phenotypes across various tumors. And in a retrospective analysis of cancer patient data, a high IL-2 pathway signature strongly correlated with response rates to PD-1 blockade. Upregulated IL-2Rα on TSTs or LCMV-specific CD8+ T cells enabled competition with Tregs for limited IL-2, allowing for productive effector T cell responses (84, 85).

In another set of studies, an IL-2/CD25 fusion protein with selectivity towards the high-affinity IL-2R was shown to better amplify cancer-vaccine activated CD4+ and CD8+ T cell responses compared to WT IL-2 treatment. Vaccine specific T cells in IL-2/CD25 fusion protein treated mice expressed higher levels of Granzyme B as well as IFN-γ, showed a less exhausted phenotype and facilitated an improved anti-tumor immune response. In these studies, a single dose of the fusion protein was administered to coincide with the expression of CD25 on vaccine activated T cells. And while the IL-2/CD25 fusion protein also led to a transient increase in Tregs, the TCR stimulation of vaccine responsive T cells led to a higher expression of CD25 in these effector T cells than in Tregs. Therefore, the tumor-specific T cells can likely effectively compete with Treg for the available IL-2/CD25 fusion protein, resulting in improved anti-tumor responses (88, 89). In a follow-up study the same authors showed that the IL-2/CD25 fusion protein also induces anti-tumor immune response in the absence of a vaccine and outperforms an IL-2Rβγc biased IL-2v (IL-2/anti-IL-2 S4B6 complexes). The fusion protein led to the expansion of CD8+ tumor infiltrating lymphocytes (TILs) with a less exhausted phenotype, expressing lower levels of PD-1 and TOX and higher levels of TCF-1. Surprisingly, while Tregs were expanded in peripheral immune tissues, no significant increase in the tumor was observed, leading to a high CD8/Treg ratio. Synergy with PD-1 treatment was observed for the high-affinity IL-2R biased fusion protein, but not for the IL-2Rβγc biased IL-2v (87).

Further data supporting the role of CD25 in the induction of optimal immune responses by IL-2 are discussed in the section “CD25-biased IL-2” as they relate to the individual IL-2v.

Together, these studies support a model wherein delivery of IL-2 to tumor-specific CD8+ T cells is required to achieve optimal IL-2 induced effector T cell responses. This targeted delivery can be achieved by IL-2Rα-biased IL-2v or the fusion of IL-2 to effector T cell targeted molecules, allowing the effector T cells to successfully compete with Treg and bystander T cells for the available IL-2. Importantly, while CD25-biased IL-2v lead to the systemic expansion of Treg and a decrease in CD8/Treg ratio, this is not the case in the tumor due to the high expression of CD25 on the effector T cells.

## 4 Novel approaches for IL-2 based immunotherapies

As discussed above, despite the remarkable anti-tumoral potential of HD IL-2 therapy, most patients do not show clinical benefits but still develop severe side effects. Numerous strategies have been developed to modify IL-2 therapy and overcome its limitations such as its short half-life, side effects and potential to expand Tregs. These approaches include modifications of IL-2 to increase its half-life to allow for reduced dosing and lower systemic Cmax, such as pegylation, fusion to human serum albumin or monoclonal antibodies and Fc domains. Fc-fusion proteins consist of the Fc region of an IgG antibody and a desired linked protein in which the Fc region can bind to the neonatal Fc receptor (FcRn) rescuing it from degradation (90). The FcRn belongs to the extensive and functionally divergent family of MHC molecules which are unable to present antigen. Instead, its capacity to bind IgG and albumin with high affinity at low pH, circumvents lysosomal degradation and prolongs the serum half-lives of proteins (91). Pegylation is a process where inert polyethylene glycol (PEG) moieties are covalently conjugated to proteins, mainly via the amino acids lysine, histidine and cysteine. Pegylation increases the hydrodynamic volume of a protein, leading to a decrease of its rapid renal clearance and increase in its half-life. It further reduces protein aggregation through repulsion between Pegylated surfaces and enhances the thermal stability of proteins (92).

Various approaches to limit systemic IL-2 exposure and selectively deliver IL-2 directly to the tumor are being explored. These include the fusion of IL-2 to tumor targeting antibodies as well as the fusion to inactivation domains with protease cleavable linkers. Furthermore, IL-2 has been encoded in oncolytic viruses (OV) that are delivered intratumorally and preferentially replicate in cancer cells (93). The encapsulation of IL-2 into polymers has also been used for localized delivery of IL-2 to the tumor microenvironment. For this purpose, IL-2 has been incorporated into various carriers such as gelatin and chondroitin-6-sulfate (94), liposomes (95), bundled carbon nanotubes (96), IL-2 encapsulating polymeric micelles (97), chitosan nanoparticles (98), and cyclodextrin (CD) nanoplexes (99) to list a few. However, while these polymer-based approaches for IL-2 delivery have shown promising results in pre-clinical studies, they have not yet undergone clinical evaluation and therefore have not been discussed further. An area that has not been considered widely in the engineering of IL-2 is the impact of the physicochemical properties of the tumor microenvironment on the activity of IL-2. In a recent publication (100) it was shown that the acidic pH in the tumor interferes with the binding of native IL-2 to CD25, resulting in reduced STAT5 phosphorylation, effector differentiation, and antitumor immunity by CD8+ T cells. A pH-sensitive IL-2v (Switch-2) that more potently activated CD8+ T cells in an acidic than neutral environment induced better anti-tumor immune responses with reduced toxicity in normal tissues than native IL-2.

An alternative strategy to avoid activation of the cell type(s) responsible for the toxicities seen with WT IL-2, is to directly target IL-2 to the immune cells of interest. To this end, IL-2 has been fused to immune cell targeting antibodies and various IL-2v have been generated that show preferential binding to certain cell types depending on their expression levels of the three IL-2R chains. Several approaches are used to generate these biased IL-2v. The introduction of amino acid mutations in the different IL-2R interfaces of IL-2 can abolish or increase binding to the three IL-2R chains. Alternatively, fusion to an antibody binding and blocking an IL-2R interface or fusion to an extracellular domain of an IL-2R chain is used to bias IL-2 binding. Similarly, pegylation is used to bias IL-2R selectivity via steric hindrance. The engineering of biased IL-2v to overcome HD IL-2 toxicities is complicated by the fact that the cell type(s) responsible for the observed side effects are not fully understood and that multiple cell types with differential IL-2R expression may contribute to the different toxicities.

**Figure 3:**
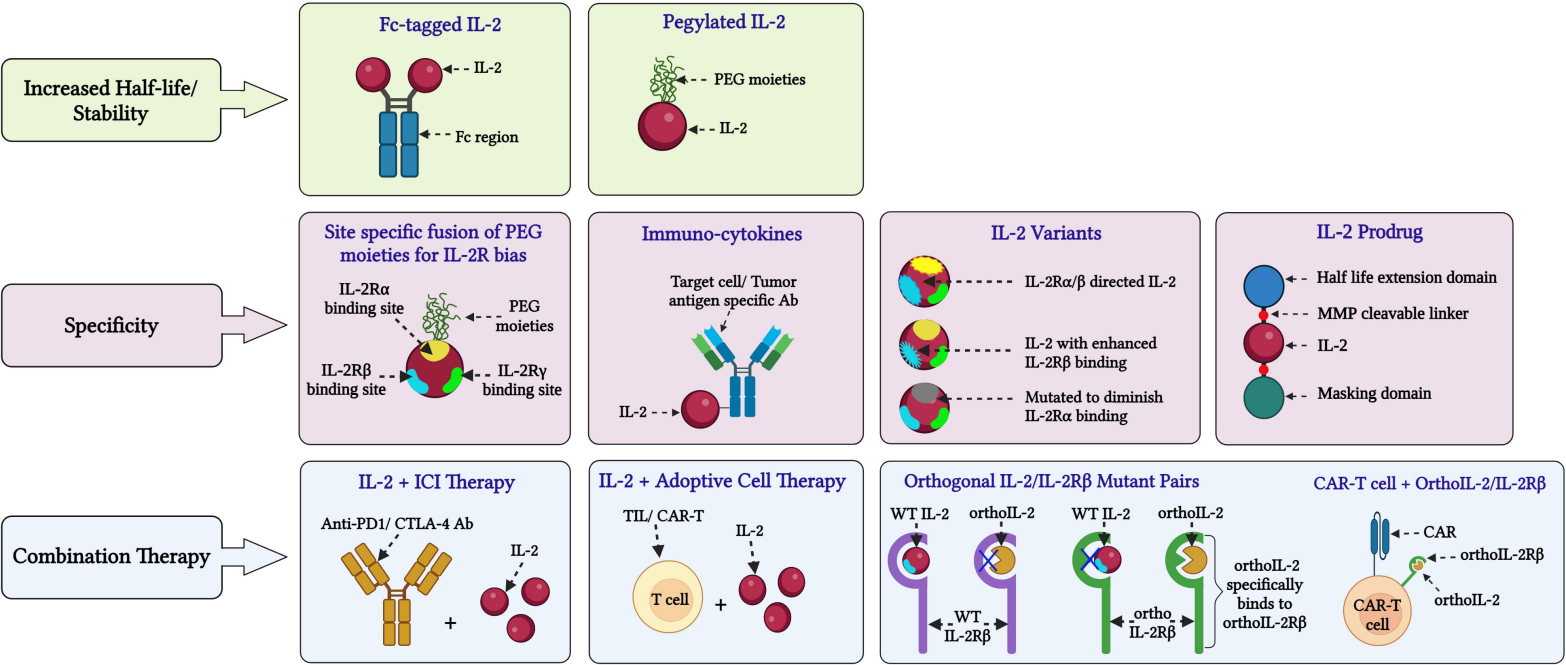
Novel approaches for IL-2 based cancer immunotherapy. Various innovative approaches are being investigated to address limitations and side effects associated with HD IL-2 therapy and enhance its efficacy in cancer treatment: **(A)** *Stability*: Fusing IL-2 with molecules like PEG and Fc to extend its half-life and improve stability by reducing rapid renal clearance. **(B)** *Specificity*: In certain formulations, PEG moieties are attached to the IL-2Rα binding domain of IL-2 to inhibit its interaction with IL-2Rα, as seen in BEMPEG. Another strategy is fusing IL-2 with the Fc region of an IgG antibody, such as in formulations like ProIL-2/sumIL2-Fc and INBRX-120, to prolong its presence in the serum. To improve the targeted binding of IL-2 to tumor-specific effector lymphocytes, different IL-2 variants have been developed. Such variants consist of IL-2 with reduced binding to IL-2Rα or enhanced binding to IL-2Rβ or reduced binding to IL-2Rγ. Examples of these engineered IL-2 formulations include IL-2 superkines, BEMPEG and STK-012, among others. To enhance the delivery of IL-2 to select target cells such as CD8+ T cells or tumor cells, IL-2 has been fused to antibodies with epitopes specific for these target cells. Notable examples of such IL-2 formulations include darleukin, APN-301 or RO7284755 and AB248 among others. **(C)** *Combinational strategies*: These approaches involve combining IL-2 therapy with immune checkpoint inhibitors like anti-PD1 or anti-CTLA-4 antibodies. Additionally, IL-2 is being combined with adoptive transfer of tumor-infiltrating lymphocytes or chimeric antigen receptor (CAR)-T cells, and CAR-T cells with engineered orthoIL-2Rβ domains that specifically bind to ortho IL-2 mutants. These therapies are currently undergoing extensive clinical investigations. (Image created with Biorender.com).

Examples of these different engineering strategies and combinations thereof will be discussed below, with a focus on molecules that have undergone or are currently undergoing clinical evaluation. Molecules are grouped based on the bias of the IL-2 moiety for the different IL-2R chains – WT IL-2, IL-2v that don’t bind CD25 and IL-2v that preferentially bind CD25 expressing cells (**Figure 3**; **Supplementary Table S1**).

### 4.1 Unbiased (WT) IL-2

#### 4.1.1 PEG-IL-2

To overcome the short half-life of native IL-2, a pegylated IL-2 (PEG-IL-2) was developed. In mice, PEG-IL-2 induced increased tumor regression relative to that achieved with HD IL-2 (101, 102). Based on these preclinical studies, PEG-IL-2 was evaluated in clinical trials. A phase I study confirmed the expected increase in half-life, with a distribution half-life of 220 min and a clearance half-life of 942 min, a 10-20-fold increase compared to recombinant IL-2, for bolus i.v. infusion. Weekly administration showed comparable toxicities to HD IL-2, with a maximum tolerated dose (MTD) of approximately 20x10^6^ IU/m^2^. However, one death and 7 grade 4 toxicities were observed at a dose of 16.5x10^6^ IU/m^2^ (41). In another phase I study undertaken in patients with mRCC and MM the MTD for weekly bolus i.v. administration was determined to be 12x10^6^ IU/m^2^. A twice a week schedule was less well tolerated (103). Further, a combination treatment starting with an initial high dose IL-2 cycle followed by weekly bolus infusions of low dose maintenance PEG-IL-2 (3-6x10^6^ IU/m^2^) for 4 weeks (Arm B) was devised and tested for efficacy. This regimen was compared with a regimen consisting of two successive cycles of HD IL-2 administration (Arm A). A total of 124 patients with MM or mRCC were randomized to either standard unconjugated IL-2 therapy or the hybrid regimen. No treatment-related mortality was observed in either group, but the use of PEG-IL-2 resulted in a significant decrease in the need for intensive care unit support (37% of cycles in Arm A versus 21% of cycles in Arm B). However, PEG-IL-2 did not show an increase in antitumor activity. The PR and CR rates for patients with RCC and melanoma were 19% and 15%, respectively, for IL-2 alone and 17% and 11%, respectively, for the IL-2 and PEG-IL-2 combination (103).

#### 4.1.2 L19-IL2 (Darleukin)

**L19-IL2 (Darleukin)** is an immunocytokine in which WT IL-2 is fused to an anti–extra domain B (EDB) mAb (L19). The L19 mAb specifically targets EDB, a tumor angiogenesis marker found in the newly formed blood vessels of most tumors but rarely in healthy tissues. Preclinical studies in rats and monkeys with L19-IL2 demonstrated an acceptable safety profile. In a phase I/II trial in patients with progressive solid tumors the toxicities were manageable and reversible with no treatment-related deaths. At a dose of 30x10^6^ IU IL-2 equivalents, treatment in all three patients had to be stopped due to CLS or associated complications. The terminal half-life of L19-IL2 was determined to be 2-3 hr. Stable disease (SD) in 17/33 patients (51%) and 15/18 with mRCC (83%) was observed after two cycles, but no objective tumor responses were seen. Median progression-free survival (PFS) of RCC patients in the expansion phase of the study was 8 months (1.5-30.5) (42). The safety and clinical activity of L19-IL2 in combination with dacarbazine in patients with MM was evaluated in a phase II study. The ORR was approximately 34%, with a CR rate of about 6%. The safety profile was in line with the known toxicities of L19-IL2 and dacarbazine (104, 105). Further, in a multicenter study, patients with stage IIIB/IIIC melanoma and cutaneous/subcutaneous injectable metastases received weekly intra-tumoral injections of L19-IL2 for 4 consecutive weeks. CR by modified immune-related response criteria (irRC) of all treated metastases was achieved in 6 patients (25%), with long-lasting responses in most cases (5 patients for ≥24 months). Objective responses (OR) were reported in 53.9% of all index lesions, 36.5% of which remained stable, while 9.5% progressed. Toxicity was comparable with that of free IL-2, and no serious adverse events were recorded. A significant temporary increase of peripheral Tregs and NK cells, sustained increase of CD4+ T lymphocytes, and decrease of myeloid-derived suppressor cells (MDSCs) were observed upon treatment (106).

#### 4.1.3 Daromun (Nidlegy)

Daromun (Nidlegy) is the combination of two immunocytokines, L19-IL2 and L19-TNFα (fibromun) where L19 is fused to hTNF-α (L19-TNFα). Through this design, daromun exhibits enhanced accumulation and prolonged presence in injected lesions, resulting in a sustained immunological impact compared to non-targeted cytokines, as confirmed in preclinical models (107). In a phase II study intralesional administration of daromun in 20 patients with unresectable stage IIIC and IVM1a MM showed 55% ORR including 1 patient with CR (5%) and 10 (50%) with PR, among 20 patients evaluable for response. Authors observed CRs in 7/13 (53.8%) non-injected lesions (4 cutaneous, 3 lymph nodes), indicating a systemic activity of the intralesional immunostimulatory treatment. Grade 1 and 2 adverse events were mostly reported except for two grade 3 injection site reactions (108). Currently, neoadjuvant intra-tumoral daromun is being studied in open-label phase III clinical trials in patients with stages IIIB and C melanoma (NCT02938299 and NCT03567889). In a recent press release (109) it was announced that Nidlegy met the primary objective of NCT02938299 demonstrating statistically significant and clinically meaningful improvement in Recurrence-Free Survival (RFS) for patients with locally advanced fully resectable melanoma.

#### 4.1.4 APN-301 (hu14.18-IL2)

**APN-301 (hu14.18-IL2)** is an immunocytokine consisting of anti-GD2 antibody fused to one molecule of WT IL-2 at the C-terminus of each heavy chain. The hu14.18 monoclonal antibody is designed to specifically bind to GD2, a disialoganglioside predominantly expressed in the cell membrane of neuroectodermal tumors, including melanoma, neuroblastoma, and select sarcomas with rare expression on normal tissues (110). This immunocytokine has shown some encouraging anti-tumoral results in several preclinical models of melanoma and neuroblastoma as well as phase I and II clinical trials in adults with melanoma and children with neuroblastoma (43, 111–115) In a phase I clinical trial in melanoma patients hu14.18-IL2 showed immune activation, reversible toxicities, and a MTD of 7.5 mg/m^2^/day (approximately 22.5x10^6^ U IL-2) when administered on day 1-3 of each 28-day cycle. Dose limiting side effects were IL-2–related and included transient, reversible hypoxia and hypotension as well as elevated liver enzyme levels. The terminal half-life was found to be 3.7 hours, which is intermediate between the half-lives of the two components (60-90 min for IL-2 and 3 days for hu14.18) (113). In the phase II study, 14 patients with measurable MM were treated i.v. with 6 mg/m^2^/day hu14.18-IL2. One patient of 14 showed PR (response rate of 7.1%) and 4 patients had SD. These PR and SD responses were short-lived, lasting only 3–4 months. Toxicities observed were largely reminiscent of toxicities associated with aldesleukin, with 2 patients developing reversible grade 3 hypotension and one patient showing grade 2 renal insufficiency with oliguria (115). Phase I testing of hu14.18-IL2 in pediatric patients with recurrent or refractory neuroblastoma demonstrated biologic activity and clinical tolerability when administered at a MTD of 12 mg/m^2^/day on days 1-3 of a 28-day cycle. Dose-limiting toxicities (DLTs) included hypotension, leukopenia and allergic reaction (114). A phase 2 trial showed clinical activity in patients with small tumor burden, with 5/23 (21.7%) responders, all of which were complete. Grade 3 and 4 toxicities included acute CLS (31.6%), hypotension (15.8%) and elevated AST/ALT levels (23.7%/21.1%) (43). As anti-GD2 mAb (ch14.18) in combination with IL-2, GM-CSF, and isotretinoin is approved as minimal residual disease (MRD) therapy for high-risk neuroblastoma patients, hu14.18-IL2 was evaluated in combination with GM-CSF and isotretinoin in recurrent or refractory neuroblastoma patients in a phase II study. A 16.1% response rate was reported (116), which is comparable to the hu14.18-IL2 monotherapy activity as described above.

In summary, targeting IL-2 to well characterized tumor antigens recapitulated IL-2 mediated anti-tumor efficacy but largely failed to avoid serious treatment related toxicities.

#### 4.1.5 WTX-124

**WTX-124** is an inactive cytokine prodrug that contains IL-2 fused to an inactivation domain and a half-life extension domain via protease-cleavable linkers. These linkers are preferentially cleaved by tumor-associated proteases, releasing fully active, WT IL-2 in the tumor from the otherwise inactive prodrug (117). *In vitro*, cleaved WTX-124 had comparable activity to IL-2 to promote the proliferation of phytohemagglutinin (PHA) activated human T cells. In contrast, intact WTX-124 had a more than 20-fold reduced EC50 and plateaued at about 40% of the maximal activity of IL-2. *In vivo*, WTX-124 induced significant tumor growth inhibition of MC38 tumors (colorectal adenocarcinoma) and 100% complete regression at doses of 100 µg or more. The anti-tumor activity of WTX-124 was cleavage dependent as a non-cleavable version of WTX-124 showed limited effect. The activation of immune cells in the tumor was sufficient to promote tumor rejection as blockade of lymphocyte migration with FTY720 did not alter the efficacy of WTX-124. WTX-124 was well tolerated with no body weight loss or CLS observed at tested dose levels. Based on the ratio of the MTD and the minimum efficacious dose of each molecule, the therapeutic window of rhIL-2 was determined to be less than 4, but greater than 20 for WTX-124. WTX-124 treatment resulted in a large increase in tumor-specific CD8+ T cells and to a lesser extent Treg in the tumor, resulting in a significantly increased CD8/Treg ratio. In addition, the frequency of IFN-γ and Granzyme B producing CD8+ T cells in the tumor was increased. Interestingly, WTX-124 also increased the frequency of IFN-γ and TNF-α producing Treg, a phenomenon called Treg fragility that is associated with the loss of suppressive activity (118). WTX-124 is currently evaluated in a Phase I trial (NCT05479812) as monotherapy and in combination with Pembrolizumab. Preliminary data published from the first four dose levels of the monotherapy dose escalation arm - 16 patients treated with 1-12 mg IV Q2W - show a half-life of 1.9-5.8 days for WTX-124 and evidence of CD8+ T cell and NK cell expansion and activation in the tumor. Antitumor activity has been observed with an unconfirmed PR in a MM patient. WTX-124 was generally well tolerated with no cases of CLS, no DLTs and no treatment discontinuations due to adverse events reported so far (44).

#### 4.1.6 TILT-123

**TILT-123** (Igrelimogene litadenorepvec), is an oncolytic adenovirus (Ad5/3-E2F-d24-hTNF-IRES-hIL2); expressing two potent cytokines, TNF-α and IL-2, that has entered clinical development. Its design aims to stimulate T-cell infiltration into the tumor and to specifically promote T cell responses within solid tumors. TILT-123 exhibited safety and anti-tumor efficacy in preclinical animal models as monotherapy and in combination with an anti-PD-1 treatment. The virus was rapidly eliminated from healthy tissues and did not induce damage to vital organs (119, 120).

TUNIMO (NCT04695327), a single-arm, multicenter phase I dose-escalation trial was conducted to evaluate the safety of TILT-123 in advanced solid cancers that were refractory to standard therapies (121, 122). The trial utilized a 3 + 3 dose-escalation regimen, where patients received an i.v. dose of TILT-123 followed by at least five planned intratumoral (i.t.) injections. Among the twenty enrolled patients, with a median age of 58 years, the most common cancer types were sarcomas (35%), melanomas (15%), and ovarian cancers (15%). No DLTs were observed. The most frequent TRAEs included fever (16.7%), chills (13.0%), and fatigue (9.3%). TILT-123 was detected in both injected and non-injected tumors, and the virus was found in the blood following both i.v. and i.t. injections. Treatment resulted in systemic immune activation and T cell infiltration into the tumors. Ten patients were assessed for response on day 78 using RECIST 1.1, iRECIST, or PET-based criteria. The disease control rate assessed by PET was 60% and by RECIST 1.1 and iRECIST was 20%. Tumor size reductions were observed in both injected and non-injected lesions.

Overall, TILT-123 demonstrated safety and induced anti-tumor effects in local and distant lesions in heavily pre-treated patients. TILT-123 is currently further evaluated in several clinical trials (NCT04217473, NCT05271318, NCT05222932, and NCT06125197) as monotherapy or in combination with other cancer immunotherapies such as PD-1/PD-L1 pathway inhibitors (121, 122). Despite the promising safety and efficacy profile of TILT-123 in patients, i.t. injections remain a primary method of OV administration to achieve localized concentrations that can elicit effective immune responses. However, such repeated i.t. injections present significant challenges for clinical development. There is an urgent need to optimize OV delivery methods to enhance patient convenience and induce more potent systemic anti-tumor effects.

### 4.2 Non-alpha IL-2

#### 4.2.1 NKTR-214 (Bempegaldesleukin)

NKTR-214 (Bempegaldesleukin/ BEMPEG) is an engineered PEGylated IL-2 with prolonged half-life, containing an average of six releasable PEG chains. When fully PEGylated, it is an inactive prodrug with a gradual increase of its bioactivity as PEG molecules are slowly released after administration. *In vivo*, the half-life of the active variant is relatively short (~10 hour). It exhibits limited binding to IL-2Rα, thus biasing signaling to favor the dimeric intermediate affinity IL-2Rβγc, predominantly expressed on Teff and NK cells. In a murine melanoma tumor model, BEMPEG reduced tumor growth by enhancing the proliferation, activation, and effector function of CD8+ T and NK cells without intra-tumoral expansion of Tregs. There was a marked elevation of the CD8/Treg ratio (greater than 400) in the tumor by day 7 of treatment (123). BEMPEG in combination with anti-PD-1 and anti-CTLA-4 checkpoint blockade therapy or peptide-based vaccination in a wide variety of murine tumor models showed superior anti-tumor activity over infrequently dosed native IL-2 and systemically expanded anti-tumor CD8+ T cells while reducing Tregs in tumor tissue but not in the periphery. These findings demonstrated that BEMPEG synergizes with T cell-mediated anti-cancer therapies (124). As described above, unbiased PEG-IL-2 has been shown to have improved *in vivo* efficacy over native IL-2 in murine tumor models. Therefore, it would have been helpful to see a direct comparison of the unbiased PEG-IL-2 and BEMPEG to better understand the contribution of the extended exposure versus the bias towards the dimeric IL-2 receptor to the *in vivo* activity of BEMPEG.

In the first in-human multicenter phase I study, BEMPEG was well tolerated with grade 3 treatment related adverse events (TRAEs) experienced by 21.4% of patients and no grade 4 TRAEs or any treatment-related deaths, but included CLS associated TRAEs like hypotension, flu-like symptoms, pyrexia and lymphopenia. While some disease stabilization was seen, overall BEMPEG did not show single-agent activity. Increased immune activation and numbers of immune cells were evident in the periphery across all doses and cycles with no loss of activity upon repeated administration. Results of tumor biopsies demonstrated that BEMPEG led to immune cell increase with limited increase of Tregs. Transcriptional analysis of tumor biopsies revealed engagement of the IL-2R pathway and significantly increased expression of genes associated with an effector phenotype. Based on safety and pharmacodynamic markers, the recommended phase II dose was determined as 0.006 mg/kg every three weeks (45).

In the phase I/II PIVOT-02 trial, the combination of BEMPEG plus nivolumab (NIVO) was well-tolerated and demonstrated exciting clinical activity as first-line therapy in MM. The combination elicited durable responses, showing an ORR of 53% (n = 20/38) and a CR rate of 34% (n = 13/38). 47.4% (18 of 38 patients) experienced complete clearance of target lesions and median PFS was 30.9 months with 24-month OS rate of 77% (46). Encouraging safety and efficacy profiles in combination with immune checkpoint inhibitors (ICI) were also reported in single arm studies for other types of cancers including RCC (125), triple-negative breast cancer (126), urothelial cancer (UC) (127), and non-small cell lung cancer (NSCLC) (125).

Despite these encouraging outcomes from the initial stage clinical trials, the combination of BEMPEG and NIVO missed its primary end-points of ORR, progression free survival (PFS), and overall survival (OS) in two phase III trials. These included the PIVOT-09 study, evaluating BEMPEG in combination with NIVO versus investigator’s choice of sunitinib or cabozantinib [VEGF-targeted tyrosine kinase inhibitor (TKI)] for previously untreated, advanced RCC. The ORR was 23.0% for BEMPEG plus NIVO and 30.6% for the TKI arm. The median OS was 29.0 months for BEMPEG plus NIVO and not reached for the TKI arm at a median duration of follow-up of 15.5 months (128). In the PIVOT IO-001 trial, BEMPEG in combination with NIVO was compared against NIVO monotherapy in metastatic melanoma. Here, the ORR for BEMPEG plus NIVO was 27.7% versus 36.0% with NIVO alone. CRs occurred in 8.1% of the patients in the BEMPEG plus NIVO arm and in 12.5% in the NIVO arm. The median OS was 29.67 months with BEMPEG plus NIVO versus 28.88 months with NIVO alone. The efficacy of the NIVO monotherapy was comparable to that seen in previous clinical trials (129, 130). Similar to the BEMPEG plus NIVO combo, other treatment regimens have shown promising results in early trials, but failed to achieve meaningful efficacy and safety in phase III trials (131). Potential reasons for these different outcomes are the small patient number and non-randomized nature of the early trials, a bias in patient selection and a lower risk tolerance in the larger phase III trials (132).

In addition to missing the efficacy endpoints, the BEMPEG plus NIVO combination also showed a higher incidence of adverse events than NIVO alone. In patients who received the combination versus those who received NIVO, TRAEs occurred in 88.6% of patients that received BEMPEG plus NIVO versus 69.1% of patients treated with NIVO alone. Grade 3/4 TRAEs were 21.7% versus 11.5% and treatment-related serious AEs were 14.0% and 6.8%, respectively (133). These results led to the termination of the clinical development of BEMPEG and had an impact on other βγc-biased IL-2v in development.

#### 4.2.2 SAR444245 (THOR-707)

SAR444245 (THOR-707) is a PEGylated recombinant non-alpha IL-2v, developed by Synthorx, that is irreversibly bound to a PEG chain through a novel unnatural amino acid *via* click chemistry. It lacks IL-2Rα binding while retaining near-native affinity for the IL-2Rβγc subunits (134).

In mice and non-human primates (NHP), SAR444245 demonstrated increased half-life (terminal half-life of 13.3 hour) and an improved pharmaco-kinetics profile. SAR444245 dosing induced sustained pSTAT5 signaling in peripheral CD8+ T cells and NK cells in naïve mice and NHP. In naïve mice, SAR444245 drove stronger activation and proliferation of peripheral effector T and NK cells than WT IL-2 without significant Treg expansion. SAR444245 also induced substantially lower levels of IL-5, which has been proposed to promote CLS. In NHP a dose of 100 µg/kg resulted in maximal expansion of peripheral CD8+ T cells. No indication of CLS was observed at dose levels of up to 1000 µg/kg, but diarrhea developed in some animals (135). In murine models of syngeneic B16-F10 melanoma and CT-26 colon cancer, SAR444245 showed enhanced drug accumulation in tumor tissues, induced Ki67 expression in and expansion of tumor-infiltrating CD8+ T and NK cells, increased intra-tumoral T cell repertoire diversity and led to a dose-dependent reduction of tumor growth. However, no CR or tumor regression were shown in these two mouse models and the activity of SAR444245 was not compared to WT IL-2 or PEG-IL-2. SAR444245 administration showed additive efficacy in combination with PD-1 checkpoint inhibitor therapy in CT-26 tumor-bearing mice with a 36% CR (134, 136).

A phase I/II study of SAR444245 administered via i.v. infusion as monotherapy and in combination with pembrolizumab or cetuximab for patients with advanced/metastatic solid tumors began in 2019. The half-life was 9-12 hour and the recommended phase 2 dose was 24 µg/kg once every 3 weeks (Q3W). No DLTs or anti-drug antibodies (to IL-2 or PEG) were observed in these cohorts. Most common TRAEs were fever (48%), flu-like symptoms (45%), and nausea (35%) following the first dose which was resolved with standard supportive care. Among 58 patients treated with SAR444245 monotherapy, one patient with a head and neck squamous cell carcinoma (HNSCC) had a confirmed PR (47). There was a dose-dependent increase in peripheral CD8+ (effector and memory) T cells, and NK cells up to 32 μg/kg by 1.7 - 6.3 and 3.8 - 29 fold, with a plateau/decline at 40 μg/kg. Treg numbers increased at doses above 24 μg/kg and a modest increase in the CD8/Treg ratio of 1.4 - 2.5 fold was seen. No significant increases in IL-5 levels or eosinophil counts were observed in this study (47, 48). In October 2022, Sanofi announced the decision to halt the ongoing phase II trials with the current dosing schedule of once every 3 weeks as the efficacy observed in the initial analysis of the data was lower than anticipated. Though this decision is not based on any concerns over safety profile. Considering the emerging data from internal and external sources on mechanism of action and therapeutic potential of non-alpha IL-2, a new phase I/II program is planned for SAR444245. This program will primarily concentrate on schedule intensification to solidify the foundation for achieving a superior target profile (137).

#### 4.2.3 NL-201

NL-201 is based on Neoleukin-2/15 (Neo-2/15) with a cysteine mutation at position 62 to allow for site-specific conjugation of a 40 kDa PEG polymer for half-life extension. Neo-2/15 is a synthetic, *de novo* computer-designed IL-2 mimic with an amino acid sequence unrelated to IL-2. It binds to the intermediate IL-2Rβγc receptor with higher affinity than IL-2 but completely lacks binding to IL-2Rα (or IL-15Rα) (138). It has therefore increased potency on CD8+ T cells and NK cells while eliminating the bias of native IL-2 toward IL-2Rα expressing cells such as Tregs, endothelial cells and ILC2. While NL-201 was shown to be less potent than Neo-2/15 in inducing IL-2R signaling *in vitro*, it remained more potent than WT IL-2 (139). Preclinical efficacy was seen in several mouse tumor models, including CT26 (colorectal carcinoma), MC38 (colon adenocarcinoma), EMT6 (breast carcinoma) and LL/2 (lung carcinoma), when dosed i.v. QWx2 at 150-375 µg/kg (140). NL-201 was well tolerated in cynomolgus monkeys dosed QWx5 at 5, 15 and 50 µg/kg, with no detectable hypotension and in the absence of severe capillary leak. As expected, based on the *in vitro* and mouse data, NL-201 stimulated sustained, dose dependent CD8+ T cell and NK cell proliferation. Importantly, as NL-201 is a synthetic protein with an amino acid sequence not found in nature, only infrequent, low-titer Anti-Drug antibodies (ADAs) were observed in NHPs that did not impact tolerability or activity. A phase I clinical trial (NCT04659629) evaluating NL-201 as monotherapy and in combination with pembrolizumab was initiated, with a planned dose range from 0.1-24 µg/kg administered every 21 days or on day 1 and 8 of each 21-day cycle (141). However, based on a review of preliminary data and recent developments in the field of IL-2 therapeutics further clinical development of NL-201 was discontinued (142).

#### 4.2.4 ALKS-4230 (Nemvaleukin alfa)

ALKS-4230 (Nemvaleukin Alfa), developed by Alkermes, is an engineered fusion protein of a circularly-permuted IL-2 fused with the extracellular domain of IL-2Rα, that selectively activates effector lymphocytes bearing the intermediate-affinity IL-2Rβγc and inhibits the interaction with IL-2Rα (143).

*In vitro* studies with primary human cells derived from healthy donors and advanced cancer patients demonstrated that nemvaleukin induced greater activation and expansion of NK cells with limited expansion of Tregs. In mice, compared to rhIL-2, nemvaleukin administration led to greater expansion of NK cells and CD8+ memory T cells at doses that did not expand or activate Tregs and demonstrated superior antitumor efficacy in the mouse B16-F10 lung tumor model. The half-life and mean residence time of nemvaleukin was 4-5-fold longer and 8-fold longer than rhIL-2, respectively (143).

Recent findings from ARTISTRY-1, a phase I/II clinical study of nemvaleukin alone and in combination with pembrolizumab through i.v. administration in patients with advanced solid tumors revealed encouraging findings with preliminary clinical benefits and acceptable tolerability profiles. In ARTISTRY-1, TEAEs included hypotension, liver function test (LFT) elevation and pyrexia and chills in more than half of the patients. The recommended phase 2 dose (RP2D) for nemvaleukin monotherapy of 6 µg/kg i.v. on days 1 to 5 of a 21-day cycle elicited durable and deep responses in patients with advanced melanoma and RCC. At this dose the half-life was about 5 hour. In pharmacodynamic studies, nemvaleukin monotherapy stimulated robust expansion of CD8+ T and NK cells, with minimal effect on Tregs. Patients in the monotherapy group showed durable antitumor activity, including in RCC [ORR, 18.2% (4/22)] and in melanoma [ORR, 8.7% (4/46)], with 2 PRs (1 unconfirmed) in 30 patients with cutaneous melanoma (CM); (ORR, 6.7%) and 2 PRs (1 unconfirmed) in 6 patients with mucosal melanoma (ORR, 33.3%). Combination therapy with pembrolizumab also revealed durable antitumor activity [ORR, 16.1% (22/137)]; disease control rate (DCR), 59.9%], including in platinum-resistant ovarian cancer [ORR, 28.6% (4/14); DCR, 71.4%], with 2 CRs and 2 PRs (1 unconfirmed) in 14 patients. The US FDA granted nemvaleukin a Fast Track designation for treatment of mucosal melanoma and platinum resistant ovarian cancer, and Orphan Drug designation for mucosal melanoma (49). In the ARTISTRY-2 trial, the subcutaneous (s.c.) RP2D of 3 mg every 7 days (q7d) demonstrated pharmacodynamic effects consistent with those of i.v. delivery. These findings support evaluation of nemvaleukin among patients with advanced mucosal melanoma or CM. Additionally, ARTISTRY-3 is evaluating less frequent i.v. nemvaleukin administration in patients with advanced solid tumors. A RP2D of 35 µg/kg was determined for Q3W monotherapy administration with a comparable expansion of NK cells and CD8+ T cells as was seen for the more frequent dosing in ARTISTRY-1. The observed half-life was about 9-10 hour (144). ARTISTRY-6 is an ongoing phase II trial of nemvaleukin, where patients with advanced CM will receive nemvaleukin at the s.c. RP2D of 3 mg q7d, patients with advanced mucosal melanoma will receive nemvaleukin at the i.v. RP2D of 6 µg/kg on days 1-5 per 21-day cycle and patients with advanced CM will receive i.v. nemvaleukin less frequently (1 or 2 doses/cycle). The treatment will continue until progression or intolerable toxicity where the primary objective is to evaluate the antitumor activity of nemvaleukin monotherapy defined by overall response rate (145). ARTISTRY-7, a phase III clinical trial of nemvaleukin as monotherapy and in combination with pembrolizumab against standard chemotherapy in platinum-resistant ovarian, fallopian tube, and primary peritoneal cancer began in October 2021 and is currently under evaluation.

#### 4.2.5 Cergutuzumab amunaleukin (CEA-IL2v, RO6895882/RG7813) and Simlukafusp alfa (FAP-IL2v, RO6874281/RG7461)

Cergutuzumab amunaleukin (CEA-IL2v, RO6895882/RG7813) and Simlukafusp alfa (FAP-IL2v, RO6874281/RG7461) are immunocytokines containing the same IL-2Rβγc biased IL-2v fused to an antibody against carcinoembryonic antigen (CEA) or fibroblast activation protein α (FAP), respectively (146, 147). The IL-2v carries the mutations F42A, Y45A and L72G in the IL-2Rα binding region of IL-2 and has abolished IL-2Rα binding. In both immunocytokines, the antibodies have the P329G, L234A, L235A mutations in the Fc portion abolishing binding to FcγRs and C1q and thus avoid unspecific and systemic FcγR mediated activation of innate immune effector cells. Both antibodies also have the knob-in-hole design and IL-2v is fused to the C-terminus of the knob containing heavy chain. Therefore, both immunocytokines contain only one IL-2v molecule and avoid avidity effects for IL-2R expressing cells. This design, together with the nM affinity of IL-2v for the IL-2Rβγc and the pM affinity of the antibodies for their respective targets, was chosen to avoid sequestration of the immunocytokines in a peripheral high-affinity IL-2Rαβγc sink leading to reduced clearance, and subsequently a relatively higher uptake in CEA- or FAP-positive tumors than CEA- or FAP-IL2wt.

Indeed, CEA-IL2v showed two-fold reduced clearance compared to CEA-IL2wt, but was still cleared faster than the parental CEA antibody. Biodistribution studies in mice showed that CEA-IL2v had a significantly higher tumor uptake and significantly lower spleen uptake than CEA-IL2wt. As expected, CEA-IL2v induced a preferential expansion of CD8+ T cells, NK cells and γδ T cells compared to CEA-IL2wt in the tumor, but also in blood and lymphoid tissues. CEA-IL2v showed monotherapy efficacy and combination efficacy with an anti-PD-L1 antibody as well as antibody-dependent cellular cytotoxicity (ADCC)-competent antibodies in mouse tumor models. Similar results were obtained with FAP-IL2v. CEA-IL2v was evaluated in phase I clinical trials in CEA positive solid tumors as a single agent (NCT02004106) and in combination with atezolizumab (NCT02350673). No results for these studies have been posted and there are currently no active trials for CEA-IL2v. FAP-IL2v was evaluated in several phase I clinical trials in FAP positive solid tumors as a single agent and in combination with trastuzumab or cetuximab (NCT02627274), in combination with pembrolizumab (NCT03875079) and in combination with atezolizumab with/without bevacizumab (NCT03063762). A phase I trial evaluating multiple immunotherapy-based treatment combinations, including Simlukafusp alfa, in metastatic pancreatic cancer is currently still recruiting (NCT03193190). The only phase II trial (NCT03386721) conducted to date was terminated as “The Sponsor discontinued the development of Simlukafusp alfa due to portfolio prioritization, not due to any safety, efficacy, or quality issues” (148).

#### 4.2.6 XTX202

An alternative approach to restrict activity of an IL2βγc biased IL-2v to the tumor microenvironment is used in the design of XTX202. An IL-2v (mutations are not publicly disclosed) is fused to a masking domain as well as to a half-life extension domain via a protease-cleavable linker. After unmasking by matrix metalloproteases that are preferentially active in the tumor microenvironment, XTX202 binds to the IL-2 receptor β and γc subunits and drives immune cell activation in the tumor, but not the periphery. *In vitro*, the IL-2 mutations reduced Treg activation ~ 1200-fold and improved CD8/Treg ratio by ~ 600-fold as measured by pSTAT5 activation in human PBMCs. In syngeneic mouse tumor models XTX202 showed similar efficacy to aldesleukin, but in the absence of peripheral immune activation, weight loss and lung edema. In non-human primates, repeat dosing of XTX202 was well tolerated up to 30 mg/kg (149). A phase I/II clinical trial (NCT05052268) evaluating XTX202 in patients with advanced solid tumors is currently ongoing. In the initial dose escalation phase XTX202 is administered once every 21 days, starting at a dose of 0.27 mg/kg and up to 21 mg/kg (150). Preliminary data published for 62 patients treated with up to 4 mg/kg XTX202 shows an increase in CD8+ T cells in the tumor across all dose levels as well as a dose dependent increase of CD8+ T cell and NK cell proliferation in the periphery. Treg expansion was neither seen in the periphery nor the tumor. Best response reported so far is stable disease in 13 of 42 evaluable patients. XTX202 is generally well tolerated with no cases of CLS reported and no treatment discontinuations due to TRAEs (51).

#### 4.2.7 ProIL-2

ProIL-2 is an IL-2 prodrug containing a CD8+ T cell-preferential IL-2v fused to the N-terminus of one arm of a human WT IgG1 Fc and IL-2Rβ linked to the second Fc arm via a matrix metalloproteinases (MMPs) cleavable linker. ProIL-2 is cleaved and activated by these tumor-associated proteases. ProIL-2 contains the “superkine” IL-2 mutations to increase binding affinity for IL-2Rβ and the F42A mutation to reduce IL-2Rα binding (151, 152). In preclinical mouse models of MC38 (colorectal cancer), B16 (melanoma), CT26 (colorectal cancer), and 4T1 (breast cancer), ProIL-2 has been shown to preferentially activate and expand antigen-specific CD8+ T cells intra-tumorally, significantly reducing IL-2 toxicity and mortality without compromising antitumor efficacy (153).

#### 4.2.8 MDNA11

MDNA11 is an IL-2v with a 30-fold increased affinity for IL-2Rβ over native IL-2 and no binding to IL-2Rα (154). It is fused to human albumin for half-life extension. MDNA11 is based on the H9 IL-2 superkine (L80F, R81D, L85V, I86V and I92F) published by the Garcia lab (151) and has two additional amino acid changes (F42A and E62A) blocking the interaction with IL-2Rα. MDNA11 shows increased activation of naïve CD8+ T cells and NK cells with limited eosinophil stimulation compared to native IL-2. Preclinical efficacy, which was dependent on CD8+ T cells and NK cells, was seen in several mouse tumor models (B16, MC38 and CT26) when dosed weekly at 2-5 mg/kg (154). MDNA11 was well tolerated in cynomolgus monkeys dosed biweekly at 0.15 mg/kg and 0.3 mg/kg, with adverse events observed at 0.6 mg/kg that most frequently included transient diarrhea and lethargy (154). A phase I/II clinical trial (NCT05086692) evaluating MDNA11 as monotherapy and in combination with checkpoint inhibitor recently completed its dose escalation part and has commenced monotherapy dose expansion trial. MDNA11 has a half-life of less than 8 hour in patients and was administered i.v. once every 2 weeks (Q2W) at doses ranging from 3 µg/kg to 120 µg/kg in 6 dose escalation cohorts. No dose limiting toxicities were reported, potentially due to the relatively short residence time of less than 2 days. Based on PD markers such as the strong expansion of total T cells including activated CD25+CD8+ T cells, a dose of 90 µg/kg was selected for the expansion cohort. In 30 patients in the escalation cohorts, two durable PRs [CM, micro-satellite instability (MSI) high pancreatic cancer] and seven patients with SD (two with a duration of more than 6 months in melanoma patients) were reported with best responses seen at doses of 60 µg/kg and above (155).

#### 4.2.9 AU-007

AU-007 is a computationally designed antibody against the IL-2Rα binding domain of IL-2 that blocks the binding of IL-2 to the trimeric, but not dimeric IL-2R. *In vitro*, AU-007 increased the EC50 of IL-2 for STAT5 phosphorylation in human IL-2Rα+ Treg to a level comparable to IL-2Rα- NK cells. AU-007 inhibited the proliferation of Treg in anti-CD3/anti-CD28 activated human PBMCs while effector T cells underwent expansion comparable to control. This data indicates that AU-007 captures endogenous IL-2 and prevents the Treg expansion autoinhibitory loop caused by endogenous IL-2 secreted from activated effector T cells. In preclinical mouse studies, administration of AU-007 in combination with IL-2 induced effector T cell and NK cell proliferation in the absence of Treg expansion and showed anti-tumor efficacy in a B16F10 melanoma model (156). AU-007 is currently evaluated in a phase I study (NCT05267626) as a monotherapy or in combination with low-dose aldesleukin. Escalating doses of AU-007 given i.v. Q2W are evaluated in Arm 1A. Arm 1B evaluates fixed dose AU-007 (Q2W) plus one low-dose aldesleukin s.c. injection, with escalating aldesleukin doses in sequential cohorts. Arm 1C evaluates AU-007 plus escalating doses of concomitant low-dose s.c. aldesleukin, both Q2W. Preliminary data from 24 treated patients across the three arms published recently show that AU-007 is well tolerated with no DLTs at doses up to 9 mg/kg of AU-007 alone, 4.5 mg/kg plus one 45,000 IU/kg aldesleukin dose, and 4.5 mg/kg plus 15,000 IU/kg aldesleukin Q2W. Treg and eosinophil levels decreased in the serum, while NK and CD8 T cell concentrations trended upwards. Of the response evaluable patients, a best response of SD occurred in 4 patients in Arm 1A and 1 patient in Arm 1B (157).

### 4.3 Target cell directed non-alpha IL-2

A number of IL-2Rβγc biased IL-2v fused to immune cell targeted antibodies are in preclinical development or have recently entered the clinic, including eciskafusp alfa, an anti-PD1-IL-2v from Roche (RO7284755), BPT331, an anti-PD1-IL-2v from BrightPeak Therapeutics, AB248, an anti-CD8βb-IL-2v from Asher Bio and INBRX-120, an anti-CD8αa-IL-2v from Inhibrx. These molecules aim to further restrict IL-2 exposure to cell types of interest compared to non-targeted IL-2v and overcome the requirement of WT IL-2 for IL-2Rα binding to effectively activate CD8+ T cell responses. While the IL-2v in each molecule avoids IL-2Rα, they differ in their IL-2Rβ binding. The IL-2v in RO72684755 has WT affinity for IL-2Rβ (146), the IL-2v in BPT331 has enhanced affinity (158), while the IL-2vs in AB248 and INBRX-120 have reduced affinity for IL-2Rβ (159, 160).

#### 4.3.1 RO7284755

The immunocytokine RO7284755 contains the same IL-2Rβγc biased IL-2v as Roche’s first-generation IL-2 immunocytokines, Cergutuzumab amunaleukin and Simlukafusp alfa, fused to a PD-1 blocking antibody. *In vitro*, PD1-IL-2v was approximately 40x more potent in inducing IL-2R signaling as measured by STAT5 phosphorylation in pre-activated PD-1+ human CD4+ T cells than the not-T cell targeted FAP-IL-2v (87). PD1-IL-2v was also about 30x more potent than FAP-IL-2v in inducing GM-CSF and Granzyme B secretion, showing comparable potency to WT IL-2 with intact IL-2Rα binding. Due to the much higher expression of PD-1 on a population of effector memory CD8+ TILs compared to other T cell subsets, PD1-IL-2v preferentially expanded effector memory CD8+ TILs in murine tumor models. Further analysis showed that PD1-IL-2v expanded PD-1+TCF-1+ stem-like CD8+ TILs and significantly increased the frequency of a granzyme B+ TIM-3^-^ population within the more differentiated PD-1+ TCF-1low/- CD8+ TILs. This effector T cell population was less exhausted and had a higher functional effector profile (termed “better effectors’’). In contrast, the combination of the anti-PD-1 blocking antibody with FAP-IL-2v did not expand this unique CD8+ T cell subset, but instead led to the accumulation of terminally differentiated and exhausted granzyme B- TIM-3^+^ T cells. Consequently, RO7284755 showed significantly improved anti-tumor activity in multiple mouse models compared to the combination of the anti-PD-1 antibody and FAP-IL-2v. RO7284755 is currently evaluated in a phase I clinical trial as monotherapy and in combination with atezolizumab in patients with solid tumors (NCT04303858). AB248 is currently being evaluated in a Phase Ia/Ib clinical trial as a monotherapy and in combination with Keytruda (pembrolizumab) in patients with solid tumors (NCT05653882).

#### 4.3.2 INBRX-120

INBRX-120 is a CD8α-targeted Cisleukin™ molecule consisting of a detuned, low-affinity IL-2v connected via an effector-silenced Fc domain to two high affinity CD8α-targeted single domain antibodies (sdAbs). The highly reduced IL-2R affinity of the detuned IL-2v was achieved through mutations in the IL-2Rα and IL-2Rβ binding regions of IL-2. CD8α is part of the heterodimeric CD8αβ co-receptor and a homodimeric CD8αα complex which are expressed on cytotoxic CD8+ T cells and other cytotoxic cell types including NK cells and a small subset of γ/δ T cells (161). INBRX-120 selectively targeted these cells and enhanced the proliferation and cytotoxic potential of CD8+ T cells and NK cells without activation of Tregs. In contrast, a non-targeted detuned IL-2 did not induce STAT5 phosphorylation or proliferation in human CD8+ T cells *in vitro*. INBRX-120 demonstrated robust *in vivo* antitumor efficacy in syngeneic tumor models of MC38 and CT26 and a favorable pharmacokinetic (PK) and safety profile in NHP models and was well tolerated up to a dose of 1 mg/kg (160). No clinical trials have been initiated for INBRX-120.

#### 4.3.3 CUE-101

CUE-101, developed by Cue Biopharma, is a Fc fusion protein composed of a human leukocyte antigen (HLA) complex, a human papillomavirus 16 (HPV16) E7 peptide epitope, reduced affinity human IL-2 molecules, and an effector attenuated human IgG1 Fc domain. The IL-2v used contains the two-point mutations H16A and F42A. Mutation of F42A reduces IL-2 interaction with the IL-2Rα chain, whereas H16A reduces binding to the IL-2Rβ subunit. CUE-101 was tested *in vitro* and *in vivo* to assess the selective expansion and activation of HPV16 E7(11–20)-specific CD8+ T cells as an off-the shelf therapy for the treatment of HPV16-driven tumors, including HNSCC, cervical, and anal cancers. In these studies, CUE-101 demonstrated selective binding, activation, and expansion of HPV16 E7(11–20)-specific CD8+ T cells from peripheral blood mononuclear cells (PBMCs) relative to non-target cells. Administration of CUE-101 via i.v. route induced selective expansion of HPV16 E7(11–20)-specific CD8+ T cells in HLA-A2 (AAD) transgenic mice. In TC-1 tumor-bearing mice treated with mCUE-101, anti-cancer efficacy and immunologic memory was observed. Further, combination therapy with anti-PD-1 checkpoint blockade showed synergistic efficacy (162).

CUE-101-01 is an ongoing first-in-human phase I study of CUE-101 monotherapy and in combination with pembrolizumab in HLA-A*0201 patients with HPV16+ recurrent/metastatic (R/M) HNSCC. As of January 12, 2023, 67 patients received CUE-101 ranging from 0.06 to 8 mg/kg/dose. Frequent adverse events include fatigue (45%), anemia (34%), lymphopenia (22%), chills (27%), and nausea (22%). The study did not identify a maximum tolerated dose (MTD) for CUE-101 monotherapy, but a RP2D of 4 mg/kg/dose was chosen based on PK profile, PD data, and clinical evaluations. The dose escalation of CUE-101 in combination with pembrolizumab is ongoing with no DLTs identified as of the data cut-off. The PK data demonstrated a dose-dependent increase in drug exposure that was sustained with repeated dosing, while the PD data showed selective expansion of HPV-16 E7(11–20)-specific CD8+ T cells, sustained increase in NK cells and transient increase in Treg cells. Among the 19 evaluable patients treated with CUE-101 monotherapy at the RP2D, there was 1 PR and 6 with SD for ≥ 12 weeks. Of the 12 evaluable patients treated with CUE-101 plus pembrolizumab, there were 5 PRs and 2 with SD for ≥ 12 weeks (163).

### 4.4 CD25 biased IL-2

Disappointing results from the late-stage clinical trial of BEMPEG, a non-alpha IL-2, in combination with NIVO for advanced melanoma, has prompted a reassessment of IL-2 biology and engineering strategies. In addition, as discussed above, recent studies have highlighted the critical role of IL-2Rα in effective IL-2 induced anti-viral and anti-tumor responses and emphasize the pivotal importance of IL-2Rα binding in refining IL-2-based cancer immunotherapy (84–86, 88).

In this section we will discuss such IL-2Rα-biased variants that are at various stages of clinical development.

#### 4.4.1 BAY 50-4798

BAY 50-4798, was the first IL-2v tested in the clinic in a phase I trial conducted in the early 2000’s. With the introduction of a mutation in the IL-2Rβ binding face of IL-2 (N88R), BAY 50-4798 has largely abolished binding to IL-2Rβ and reduced binding to the dimeric IL-2Rβγ. The bias towards IL-2Rαβγ was aimed to specifically stimulate T cells without activation of NK cells. *In vitro* BAY 50-4798 has an increased selectivity for activated T cells over NK cells relative to WT IL-2, but retained activity on NK cells (70). BAY 50-4798 does not include a half-life extension domain resulting in a short half-life of around 2 hour and was dosed every 8 hour, days 1-5 and 15-19, which is similar to the clinical dosing regimen of aldesleukin. The induction of total lymphocyte trafficking, efficacy and toxicity of BAY 50-4798 was comparable to aldesleukin, suggesting that the selectivity of N88R was not sufficient to avoid activation of IL-2Rα- T cells and NK cells (70, 71). Alternatively, an IL-2Rα+ cell type can cause the IL-2 associated lymphocyte trafficking. No further clinical trials for BAY 50-4798 have been reported.

#### 4.4.2 NHS-IL2(D20T) (NHS-IL2LT, Selectikine, EMD521873)

To avoid a suggested direct activation of endothelial cells via an amino acid sequence resembling a component of bacterial toxins (73) and to increase IL-2’s bias to activated T cells, an IL-2v carrying a D20T mutation was generated and fused to the C-terminus of a human IgG antibody binding DNA–histone complexes that are present in the necrotic core of tumors. Surprisingly, while the free IL-2(D20T) variant was shown to maintain comparable receptor selectivity and bioactivity as native IL-2, NHS-IL2(D20T) was highly selective for activating the high-affinity IL-2R (164). In proliferation assays NHS-IL2(D20T) showed selectivity for the high-affinity IL-2R over the intermediate IL-2R of more than 1,000-fold. Surprisingly, affinity to the high and intermediate IL-2R was comparable between NHS-IL2(D20T) and NHS-IL2. NHS-IL2(D20T) showed comparable activation of TCR stimulated T cells as native IL-2 but did not induce NK cell proliferation *in vitro*. Preclinical efficacy was seen in mouse tumor models, which was dependent on CD8+ T cells and partially on NK cells, when dosed daily for only 5 doses at 20-80 µg. In NHP studies, NHS-IL2(D20T) was well tolerated using an intermittent dosing scheme of 3 days on, 18 days off, up to the high dose of 10 mg/kg tested and induced cellular responses and moderate toxicity resembling WT-IL-2 responses. NHS-IL2(D20T) was evaluated in two phase I clinical trials as monotherapy and in combination with cyclophosphamide (NCT01032681) or radiotherapy (NCT00879866). NHS-IL2(D20T) was administered on 3 consecutive days every 3 weeks at dose levels from 0.075 to 0.9 mg/kg. The DLT was grade 3 skin rash at the 0.9 mg/kg dose-level; the MTD was 0.6 mg/kg. No severe cardiovascular side-effects (hypotension or capillary leak) were observed, but also no objective tumor responses (52). A phase II study (NCT01973608) was initiated but terminated early with only 12 patients enrolled due to the sponsor’s decision to discontinue the development of NHS-IL2(D20T). The decision to discontinue development was not related to any safety or efficacy concerns.

#### 4.4.3 Ky1043 and IBI363

Ky1043 and IBI363 are fusion proteins of IL-2Rα biased IL-2v with an anti-PD-L1 or PD-1 antibody, respectively. IBI363 contains an IL-2-N88D variant, which has reduced affinity for the IL-2Rβ chain, biasing it towards cells expressing IL-2Rα (165). IL-2-N88D by itself has modest anti-tumor activity as monotherapy (85). IBI363 has entered phase I trials (NCT05290597, NCT05460767, NCT06081920, NCT06081907). Ky1043 is in preclinical development and demonstrated strong anti-tumor activity in MC-38 tumor models. Interestingly, Ky1043 has been shown to preferentially expand TCF1+ PD-1+ precursor exhausted CD8+ T cells (Tpex) in mouse tumor models (166) that have been correlated with successful responses to immunotherapies. In addition, while a large peripheral expansion of Treg was seen, this was not the case for tumor infiltrating Treg, resulting in an increased CD8/Treg ratio in the tumor, but a decreased CD8/Treg ratio in the periphery. These results indicate that peripheral Treg expansion might not limit IL-2 mediated tumor control but might limit peripheral IL-2 mediated toxicities such as CLS.

#### 4.4.4 STK-012

STK-012, an IL-2v biased towards IL-2Rα and IL-2Rβ has recently entered clinical trials (NCT05098132) (53). The mutations in STK-012 result in reduced CD132 binding and therefore STK-012 is strongly biased towards CD25 expressing cells such as tumor antigen activated T cells and away from cells with low CD132 expression such as NK cells. STK-012 also contains a 40kD PEG for half-life extension (72). *In vitro*, STK-012 showed equal activity to WT IL-2 on CD25+ T cells, but was only minimally active on NK cells. In mouse models, STK-012 showed improved anti-tumor efficacy over WT IL-2 or a non-α IL-2 and did not induce CLS. Likewise, no lung pathology was observed in NHP at doses that expanded CD25+CD8+ T cells. Interestingly, like Ky1043, STK-012 has been shown to induce peripheral, but not tumor-infiltrating Treg expansion and to induce expansion of CD8+ Tpex, resulting in an increased CD8/Treg ratio in the tumor, but a decreased CD8 (NK)/Treg ratio in the periphery (72). Patients enrolled in the phase 1 study were heavily pretreated, with 60% having 3 or more prior lines of therapy and 79% progressed on prior immune checkpoint inhibition. STK-012 had a half-life of 4 days leading to continuous high drug exposure for the three weeks dosing cycle. STK-012 was well tolerated, without dose limiting toxicities at doses up to 3 mg every three weeks. Adverse events included rash, fatigue, nausea and diarrhea, but patients had very rarely adverse events associated with capillary leak, such as hypotension, pyrexia, flu-like symptoms and peripheral edema and had no IL-2 related lymphopenia. Three in 31 patients in the dose escalation cohorts had a confirmed PR (NSCLC, RCC, head and neck cancer, one each) with several patients having durable SD. All responses were observed in patients who progressed on prior anti-PD-1 but had less than 3 prior lines of therapy. This may indicate that the STK-012 activatable, tumor reactive T cells could be exceedingly rare in patients progressing on multiple lines of anti-cancer therapies. Increasing doses lead to elevated IFN-γ in the serum and the proliferation of CD38+ CD8+ T cells in patients. Expansion of Tregs was 2-4 fold and comparable to results seen in other IL-2 therapies, while NK cells were not increased significantly (53). The efficacy in immune checkpoint resistant/refractory patients with a favorable safety profile sets STK-012 apart from other IL-2 agonists and allows the exploration of combinations of STK-012 with other successful therapy combinations.

### 4.5 Combinatorial approaches to enhance anti-tumoral potential of IL-2

IL-2 therapy in conjunction with various combinatorial approaches aims to enhance the anti-tumoral effects of IL-2 while minimizing its adverse effect by synergistically modulating various components of the immune system or targeting specific signaling pathways. This section provides an overview of the recent advancements and potential implications of combinatorial approaches in unlocking the full therapeutic potential of IL-2 for cancer treatment.

#### 4.5.1 IL-2 in combination with immune checkpoint inhibitor therapy

Immune checkpoints act as negative feedback regulators, controlling T cell-mediated inflammatory responses under normal conditions. Some of the US-FDA approved ICI therapies for clinical use include, PD-1/PD-L1 inhibitors and CTLA-4 inhibitors (167). However, certain tumor types, like microsatellite stable colorectal cancer, exhibit limited response to anti-PD-1 therapy due to an ‘immune cold’ microenvironment (168). Similarly, ICI therapies for pancreatic cancer and prostate cancer have been less effective (169). In mouse models ICI synergizes with IL-2 therapy to revitalize exhausted T cells (21). Therefore, combination therapy of checkpoint inhibitors with IL-2 is under active clinical investigation.

In a retrospective analysis, HD IL-2 showed durable anti-tumor activity in patients after progression following PD-1 pathway blockade, with a toxicity profile expected from HD IL-2. Metastatic melanoma patients displayed an increased ORR of 22.5% (170) compared to historic data from HD IL-2 therapy alone which showed an ORR of 13% (18). Further, RCC patients treated with HD IL-2 monotherapy exhibited an ORR of 25% (171), however the combination therapy with PD‐1/PD‐L1 blockade did not improve therapeutic outcomes in these patients with an ORR of 24% (170). Prospective trials evaluating the combinations of aldesleukin or various engineered IL-2v with PD-1 pathway blockade are currently underway.

#### 4.5.2 IL-2 in combination with adoptive cell therapy

ACT with TILs is a personalized cancer treatment which consists of *ex vivo* expansion of autologous tumor infiltrating CD4+ and CD8+ T lymphocytes and their reinfusion in the same patient after lymphodepletion. In most cases, these infused T cells are co-administered with HD IL-2 to facilitate engraftment of the cells (172). ACT with TILs plus bolus i.v. HD IL-2 first demonstrated clinical benefits in 86 metastatic melanoma patients with an ORR of 31% (173). Consistent ORRs ranging from 40% to 70% were observed across multiple clinical trials conducted worldwide (174, 175).

Lifileucel (LN-144), a one-time autologous TIL therapy developed by Iovance Biotherapeutics, demonstrated an ORR of 31.4%, with 8 CRs and 40 PRs following a single infusion and up to six doses of HD IL-2 in a phase II C-144-01 trial of 153 patients with heavily pretreated advanced melanoma. The median OS and PFS were 13.9 months and 4.1 months, respectively (176). Further 12 patients with advanced mucosal melanoma receiving lifileucel, at a median study follow-up of 35.7 months, experienced an ORR of 50%, median duration of response (DOR) was not reached (NR), median PFS was NR, and median OS was 19.4 months (177). In view of these findings FDA has granted accelerated approval to lifileucel for unresectable or metastatic melanoma (178).

A phase III randomized trial in advanced melanoma patients demonstrated longer PFS with TIL plus HD IL-2 therapy compared to ipilimumab therapy. In this study, the TIL group showed a median PFS of 7.2 months and 49% ORR, while the ipilimumab group had 3.1 months and 21%, respectively. Median OS was 25.8 months and 18.9 months for TIL and ipilimumab group respectively (179). Based on these findings, MM patients in the Netherlands can undergo TIL therapy under hospital exemption provided the drug is produced in the Netherlands by the National Cancer Institute or Sanquin (180).

Another treatment modality under ACT therapy includes chimeric antigen receptor (CAR) T cells, which are engineered autologous T cells expressing a synthetic CAR that can recognize tumor-associated antigens (TAAs) independently of major histocompatibility complex (MHC) presentation. 5 different CAR-T cell therapies targeting CD19, expressed on the surface of B cells, have been approved for clinical use in lymphoma and acute lymphocytic leukemia (ALL). Numerous clinical trials investigating the co-administration of IL-2 as an adjuvant with CAR-T cell therapy (NCT00924326, NCT00019136, NCT04119024, NCT03098355) have demonstrated improved persistence of CAR-T cells and long-lasting remissions in various tumor types including lymphoma, ovarian cancer, and melanoma (181). However, it should be noted that administration of high dose IL-2 is limited by the severe side effects described above. Contemporary investigations are currently tackling this concern by employing orthogonal IL-2/IL-2Rβ pairs, and a comprehensive examination of this approach will be provided in the subsequent section.

#### 4.5.3 Orthogonal IL-2/IL-2Rβ mutant pairs

The extensive implementation of ACT in cancer treatment is hindered by various limitations, including challenges in producing an adequate quantity of cells for infusion and the inability of infused T cells to persist and maintain functionality *in vivo*. In clinical settings, the concurrent administration of IL-2 has shown improvements in the survival, function, and anti-tumor activity of transplanted T cells (182). However, the use of IL-2 to enhance ACT encounters complexities due to the pleiotropic nature of IL-2, which elicits both immune stimulatory and suppressive responses in T cells as well as its potential to cause severe toxicities (183). The effectiveness of IL-2 as an adjuvant in ACT is influenced by the equilibrium between activating infused T cell subsets and endogenous T cell subsets expressing natural IL-2 receptors. To address this limitation, the Garcia group (184) devised a strategy based on receptor-ligand orthogonalization, where a mutant IL-2 cytokine and mutant IL-2R bind specifically to one another but not to their WT counterparts. In this study they utilized murine IL-2/IL-2Rβ interaction to enable *in vivo* characterization in syngeneic mouse models. The IL-2Rβ chain, which can bind to IL-2 independently and is crucial to signal transduction, was chosen as the mutant receptor. The IL-2Rβ hot-spot residues His^134^ and Tyr^135^ make numerous contacts with IL-2 (20). A double mutant of IL-2Rβ [His^134^ → Asp (H134D) and Tyr^135^ → Phe (Y135F)], referred to as orthoIL-2Rβ, lacked detectable binding to WT IL-2 even in the presence of CD25 (184). Next, using yeast display-based evolution two orthoIL-2 mutants, 1G12 and 3A10, were chosen for further characterization. The binding affinity of these mutants to orthoIL-2Rβ was comparable to that of the WT IL-2/IL-2Rβ interaction and displayed little to no detectable binding to WT IL-2Rβ. OrthoIL-2 1G12 potently activated STAT5 on orthoIL-2Rβ–transduced T cells *in vitro*, with a ~5 fold increased potency relative to WT T cells; whereas orthoIL-2 3A10 induced somewhat weaker, though selective pSTAT5 on orthoIL-2Rβ–expressing but not WT T cells. Adoptively transferred T cells in a host with an intact immune system face competition with host cells for crucial survival signals like IL-2 (185). In contrast to WT IL-2, orthoIL-2 consumption is expected to face minimal competition from endogenous cells. To test this hypothesis, the impact of IL-2 and orthoIL-2 administration on transplanted T cells and the host immune system was quantified by adoptively transferring a mixture of WT and orthoIL-2Rβ CD8+ T cells into WT mice. The results indicated that orthoIL-2 1G12 led to significant expansion of CD8+ T cells transduced with the orthoIL-2Rβ at doses equivalent to or lower than WT IL-2. At high doses and with twice-daily administration, orthoIL-2 3A10 resulted in the substantial expansion of orthoIL-2Rβ T cells in the absence of WT T cell expansion. The orthoIL-2v also resulted in the *in vivo* expansion of orthoIL-2Rβ CD4+ effector T cells and orthoIL-2Rβ CD4+ Treg cells with specificity similar to that in CD8+ T cells. Further to envisage the putative clinical applications of orthogonal IL-2/IL-2R pairs, the efficacy of tumor-specific orthoIL-2Rβ T cells in the B16-F10 mouse model of melanoma was determined. Infusion of orthoIL-2Rβ pmel-1 T cells followed by treatment with orthoIL-2 1G12 produced a significant tumor growth delay and survival advantage similar to the IL-2 treatment group. Similar antitumor responses were also observed in mice treated with orthoIL-2Rβ pmel-1 T cells and murine serum albumin fused (MSA)–orthoIL-2 3A10. The absence of therapeutic advantages from orthoIL-2 in mice receiving WT pmel-1 T cells suggests that the effectiveness of orthoIL-2 relies on the expression of orthoIL-2Rβ in pmel-1 T cells. These findings constitute the strategy to redirect the specificity of IL-2 toward engineered T cells using orthogonal IL-2 cytokine-receptor pairs, enabling the selective expansion of specific T cell subsets during adoptive cell therapy, and limiting the off-target activity and toxicity. In order to translate this approach into humans a human orthogonal IL-2 and orthogonal IL-2Rβ pair was engineered.

In a study by Zhang et al, using CD19-specific CAR-T cells, it was shown that human orthogonal IL-2 (ortho-hIL-2) induced a 1000-fold increase in ortho-hIL-2Rβ+ CAR-T cell expansion in a dose-dependent manner *in vivo*, 2 weeks after adoptive transfer into immunodeficient mice bearing CD19+ Nalm6 leukemia xenografts. In this model, ortho-hIL-2 could rescue the anti-leukemic effect of an otherwise suboptimal CAR-T cell dose. Furthermore, the administration of ortho-hIL-2 at the onset of leukemic relapse, subsequent to CAR-T cell therapy could effectively salvage a failed anti-leukemic response. These findings highlight the potential of integrating an orthogonal cytokine approach with cellular immunotherapies to enhance the antitumor effectiveness of engineered T cells (186).

A parallel study showed that the human orthogonal IL-2R and ligand system allowed for precise *in vivo* regulation of CAR-T cell expansion and activation. In these studies, a pegylated version of the orthogonal human IL-2 (STK-009) with improved pharmacokinetic properties was used. STK-009 was able to expand ortho-hIL-2Rβ (hoRb)-expressing CAR-T cells in the presence and absence of tumor antigen and maintained the presence of stem cell memory T cells and effector T cells (187). In preclinical models of human CAR-refractory lymphoma, STK-009 treatment resulted in systemic and intra-tumoral expansion and activation of hoRb-expressing anti–CD19-CD28ζ CAR-T cells (SYNCAR-001). CRs in large subcutaneous lymphomas were observed at substantially reduced CAR-T cell doses using the orthogonal IL-2 receptor/ligand system which selectively expanded and activated CAR-T cells *in vivo*. Furthermore, withdrawal of STK-009 allowed normal CAR-T cell contraction, thus limiting the CRS induced by tumor antigen–specific T cell activation. These findings indicate that the orthogonal IL-2 receptor/ligand system offers the essential *in vivo* control needed to optimize the effectiveness of CAR-T therapies (187). Recruitment of patients for a phase I clinical trial (NCT05665062) to evaluate the safety and tolerability of a combination of SYNCAR-001 and STK-009 in subjects with relapsed or refractory CD19 expressing hematologic malignancies has been initiated (188).

In a follow-up study, the ability of the STK-009/hoRb system to not only enhance the anti-tumor activity and persistence of CAR T cells in hematological but also in solid tumor models was demonstrated. STK-009 administration enhanced the anti-tumor efficacy of anti-glypican 3 (GPC3) CAR-T cells (SYNCAR-002) in highly aggressive subcutaneous and intraperitoneal hepatocellular carcinoma models. The administration of STK-009 led to a significant expansion of SYNCAR-002 within the peripheral blood, facilitating the infiltration of SYNCAR-002 cells into tumor sites. Furthermore, STK-009 treatment prompted intra-tumoral production of granzyme B and IFN-γ by SYNCAR-002, thereby demonstrating the activation of effector T cell functionality (189).

Importantly, beyond showing the benefits of specific control of CAR-T cells, the ortho-IL-2 system also confirms that the pleiotropic effects of IL-2 are not needed to induce effective anti-tumor responses. ortho-IL-2 does not lead to the activation of NK cells or systemic activation of T cells in mouse models and this data supports the idea that restricting the activity of IL-2 to tumor-antigen specific T cells via other engineering strategies can deliver significant anti-tumor efficacy.

In addition to the IL-2 molecules discussed above, several other IL-2 based treatment modalities that are under clinical trial are listed in **Supplementary Table S2**.

## 5 Concluding remarks and future perspective

IL-2 is a critical cytokine for proliferation of NK cells and effector T cells and recombinant IL-2 was one of the first cytokines approved by the US-FDA for the treatment of cancer patients, however while deep responses are achieved, the efficacy is limited to a small subset of patients and is accompanied with severe toxic side effects. The toxicity of efficacious treatment paradigms for aldesleukin have also largely prevented the successful exploration of combination strategies with other treatment modalities, including chemotherapies or other immune modulators. Since its discovery over 4 decades ago, there has been substantial progress in understanding the molecular mechanism of action of IL-2 in the activation and regulation of immune responses. Based on this information, many companies are actively working on developing novel strategies for utilizing IL-2 as an effective anti-cancer agent with reduced or controllable toxicity. Structure function relationship exploration enabled by the crystal structure of IL-2 with its receptors, have led to the rational design of various IL-2v aiming to specifically increase its anti-tumoral potential. Combinatorial approaches utilizing these novel IL-2 molecules with other treatment modalities such as immune check-point inhibitor therapy, fusion of IL-2 to carrier proteins and tumor antigen targeting antibodies, adoptive cell therapies and CAR-T cell therapies are currently rigorously evaluated in clinical trials. Despite these advancements, it is worth noting that so far none of these newer IL-2 formulations have shown significantly improved performance in advanced clinical studies. Hence aldesleukin remains the only approved IL-2 molecule for cancer immunotherapy.

Based on the findings from recent preclinical data, successful improvements of IL-2 immunotherapy, conditionally active IL-2 versions and combinatorial approaches with targeted IL-2 formulations may provide a promising solution. However, for implementation of such strategies various key factors should be taken into account. First and foremost, such therapies have to have an acceptable side effect profile, avoiding toxicities related to CLS, such as fever and hypotension to enable combination therapies in the first and second line of treatment.

Further, preclinical research indicates that the activation of a tumor specific memory T cell response is essential for anti-tumor efficacy in monotherapy and in combination with immune checkpoint inhibitors. Identification of reliable translational biomarkers for memory T cell responses in preclinical and early clinical studies is essential to identify the most promising clinical candidates, to select the most receptive patient populations and to define the most promising treatment paradigms for the monotherapy and combination therapies.

In addition to selecting the most responsive cancer indication, mutational burden and antigenic properties of the individual patient and the nature, number and duration of prior lines of treatment have to be taken into account as they impact the immune sensitivity of the tumor immune microenvironment.

This should allow the tailoring of novel IL-2 immunotherapy to specific patient populations based on their immune profile, tumor type and tumor immunogenicity and the development of combinatorial treatment regimens with well tolerated IL-2v to achieve synergistic effects which may help in improving the success rates for such therapies in clinical settings.

## Author contributions

SR: Writing – review & editing, Writing – original draft, Data curation, Conceptualization. AD: Writing – review & editing, Writing – original draft, Supervision, Resources. MO: Writing – review & editing, Writing – original draft, Supervision, Resources, Funding acquisition, Conceptualization. JE: Writing – review & editing, Writing – original draft, Data curation, Conceptualization.

## Glossary

**Table:**

ACT	Adoptive cell therapy
ADAM17	A disintegrin and metalloproteinase 17
ADCC	Antibody-dependent cellular cytotoxicity
BBB	Blood-brain barrier
Blimp1	B lymphocyte-induced maturation protein 1
CAR	Chimeric antigen receptor
CEA	Carcinoembryonic antigen
CLS	Capillary leak syndrome
CR	Complete responses
DLT	Dose-limiting toxicities
FcRn	Neonatal Fc receptor
GPC3	Anti-glypican 3
HD	High-dose
HLA	Human leukocyte antigen
HNSCC	Head and neck squamous cell carcinoma
HPV16	Human papillomavirus 16
i.p.	Intraperitoneal
i.v.	Intravenous
ICI	Immune checkpoint inhibitor
IL-2	Interleukin
IL-2R	IL-2 Receptor
IL-2v	IL-2 variants
ILC	Innate lymphoid cells
irRC	Immune-related response criteria
JAK	Janus kinase
MAPK	Mitogen-activated protein kinase
MDSCs	Myeloid-derived suppressor cells
MM	Metastatic melanoma
MMPs	Matrix metalloproteinases
mRCC	Metastatic renal-cell carcinoma
MRD	Minimal residual disease
MTD	Maximum tolerated dose
NHP	Non-human primates
NIVO	Nivolumab
NK cells	Natural killer cells
NSCLC	Non-small cell lung cancer
ORR	Objective response rate
OV	Oncolytic viruses
PD	Pharmacodynamic
PD-1	Programmed death-1
PEG	Polyethylene glycol
PFS	Progression-free survival
PI3K	Phosphoinositide 3- kinase
PK	Pharmacokinetic
PR	Partial responses
PTEN	phosphatase and tensin homolog protein
RFS	Recurrence-free survival
RP2D	Recommended phase 2 dose
s.c.	Subcutaneous
SD	Stable disease
sdAbs	Single domain antibodies
STAT	Signal transducer and activator of transcription
TCR	T cell receptor
Teff	Effector T cell
TIL	Tumor infiltrating lymphocytes
TKI	Tyrosine kinase inhibitor
Tpex	Precursor exhausted T cells
TRAEs	Treatment related adverse events
Tregs	Regulatory T cells
UC	Urothelial cancer
US-FDA	US Food and Drug Administration
WT	Wild type
