# Supplementary material for: IL-2 based cancer immunotherapies: an evolving paradigm
Source: Front Immunol. 2024 Jul 24;15:1433989. doi: 10.3389/fimmu.2024.1433989 (PMC11303236; doi:10.3389/fimmu.2024.1433989)
Supplement: Supplementary file 1 [file DataSheet_1.pdf]

PROLEUKIN® (aldesleukin)

for injection, for intravenous infusion

Rx Only

WARNINGS

Therapy with Proleukin® (aldesleukin) should be restricted to patients with normal cardiac and pulmonary functions as defined by thallium stress testing and formal pulmonary function testing. Extreme caution should be used in patients with a normal thallium stress test and a normal pulmonary function test who have a history of cardiac or pulmonary disease.

Proleukin should be administered in a hospital setting under the supervision of a qualified physician experienced in the use of anticancer agents. An intensive care facility and specialists skilled in cardiopulmonary or intensive care medicine must be available.

Proleukin administration has been associated with capillary leak syndrome (CLS) which is characterized by a loss of vascular tone and extravasation of plasma proteins and fluid into the extravascular space. CLS results in hypotension and reduced organ perfusion which may be severe and can result in death. CLS may be associated with cardiac arrhythmias (supraventricular and ventricular), angina, myocardial infarction, respiratory insufficiency requiring intubation, gastrointestinal bleeding or infarction, renal insufficiency, edema, and mental status changes.

Proleukin treatment is associated with impaired neutrophil function (reduced chemotaxis) and with an increased risk of disseminated infection, including sepsis and bacterial endocarditis. Consequently, preexisting bacterial infections should be adequately treated prior to initiation of Proleukin therapy. Patients with indwelling central lines are particularly at risk for infection with gram positive microorganisms. Antibiotic prophylaxis with oxacillin, nafcillin, ciprofloxacin, or vancomycin has been associated with a reduced incidence of staphylococcal infections.

Proleukin administration should be withheld in patients developing moderate to severe lethargy or somnolence; continued administration may result in coma.

DESCRIPTION

Proleukin® (aldesleukin), a human recombinant interleukin-2 product, is a highly purified protein with a molecular weight of approximately 15,300 daltons. The chemical name is des-alanyl-1, serine-125 human interleukin-2. Proleukin, a lymphokine, is produced by recombinant DNA technology using a genetically engineered *E. coli* strain containing an analog of the human interleukin-2 gene. Genetic engineering techniques were used to modify the human IL-2 gene, and the resulting expression clone encodes a modified human interleukin-2. This recombinant form differs from native interleukin-2 in the following ways: a) Proleukin is not glycosylated because it is derived from *E. coli* ; b) the molecule has no N-terminal alanine; the codon for this amino acid was deleted during the genetic engineering procedure; c) the molecule has serine substituted for cysteine at amino acid position 125; this was accomplished by site specific manipulation during the genetic engineering procedure; and d) the aggregation state of Proleukin is likely to be different from that of native interleukin-2.

The *in vitro* biological activities of the native nonrecombinant molecule have been reproduced with Proleukin.[1,2]

Proleukin is supplied as a sterile, white to off-white, lyophilized cake in single-use vials intended for intravenous administration. When reconstituted with 1.2 mL Sterile Water for Injection, USP, each mL contains 18 million International Units (1.1 mg) Proleukin, 50 mg mannitol, and 0.18 mg sodium dodecyl sulfate, buffered with approximately 0.17 mg monobasic and 0.89 mg dibasic sodium phosphate to a pH of 7.5 (range 7.2 to 7.8). The manufacturing process for Proleukin involves fermentation in a defined medium containing tetracycline hydrochloride. The presence of the antibiotic is not detectable in the final product. Proleukin contains no preservatives in the final product.

Proleukin biological potency is determined by a lymphocyte proliferation bioassay and is expressed in International Units as established by the World Health Organization 1st International Standard for Interleukin-2 (human). The relationship between potency and protein mass is as follows:

18 million International Units Proleukin = 1.1 mg protein

CLINICAL PHARMACOLOGY

Proleukin® (aldesleukin) has been shown to possess the biological activities of human native interleukin-2.[1,2] *In vitro* studies performed on human cell lines demonstrate the immunoregulatory properties of Proleukin, including: a) enhancement of lymphocyte mitogenesis and stimulation of long-term growth of human interleukin-2 dependent cell lines; b) enhancement of lymphocyte cytotoxicity; c) induction of killer cell (lymphokine-activated (LAK) and natural (NK)) activity; and d) induction of interferon-gamma production.

The *in vivo* administration of Proleukin in animals and humans produces multiple immunological effects in a dose dependent manner. These effects include activation of cellular immunity with profound lymphocytosis, eosinophilia, and thrombocytopenia, and the production of cytokines including tumor necrosis factor, IL-1 and gamma interferon.[3] *In vivo* experiments in murine tumor models have shown inhibition of tumor growth.[4] The exact mechanism by which Proleukin mediates its antitumor activity in animals and humans is unknown.

Pharmacokinetics

Proleukin exists as biologically active, non-covalently bound microaggregates with an average size of 27 recombinant interleukin-2 molecules. The solubilizing agent, sodium dodecyl sulfate, may have an effect on the kinetic properties of this product.

The pharmacokinetic profile of Proleukin is characterized by high plasma concentrations following a short intravenous infusion, rapid distribution into the extravascular space and elimination from the body by metabolism in the kidneys with little or no bioactive protein excreted in the urine. Studies of intravenous Proleukin in sheep and humans indicate that upon completion of infusion, approximately 30% of the administered dose is detectable in plasma. This finding is consistent with studies in rats using radiolabeled Proleukin, which demonstrate a rapid (<1 min) uptake of the majority of the label into the lungs, liver, kidney, and spleen.

The serum half-life (T 1/2) curves of Proleukin remaining in the plasma are derived from studies done in 52 cancer patients following a 5-minute intravenous infusion. These patients were shown to have a distribution and elimination T 1/2 of 13 and 85 minutes, respectively.

Following the initial rapid organ distribution, the primary route of clearance of circulating Proleukin is the kidney. In humans and animals, Proleukin is cleared from the circulation by both glomerular filtration and peritubular extraction in the kidney.[5-8] This dual mechanism for delivery of Proleukin to the proximal tubule may account for the preservation of clearance in patients with rising serum creatinine values. Greater than 80% of the amount of Proleukin distributed to plasma, cleared from the circulation and presented to the kidney is metabolized to amino acids in the cells lining the proximal convoluted tubules. In humans, the mean clearance rate in cancer patients is 268 mL/min.

The relatively rapid clearance of Proleukin has led to dosage schedules characterized by frequent, short infusions. Observed serum levels are proportional to the dose of Proleukin.

CLINICAL STUDIES

Safety and efficacy were studied in a series of single and multicenter, historically controlled studies enrolling a total of 525 patients with metastatic renal cell carcinoma or melanoma. Eligible patients had an Eastern Cooperative Oncology Group (ECOG) Performance Status (PS) of 0 or 1 and normal organ function as determined by cardiac stress test, pulmonary function tests, and creatinine ≤1.5 mg/dL. Studies excluded patients with brain metastases, active infections, organ allografts and diseases requiring steroid treatment.

The same treatment dose and schedule was employed in all studies demonstrating efficacy. Proleukin was given by 15 min intravenous infusion every 8 hours for up to 5 days (maximum of 14 doses). No treatment was given on days 6 to 14 and then dosing was repeated for up to 5 days on days 15 to 19 (maximum of 14 doses). These 2 cycles constituted 1 course of therapy. Patients could receive a maximum of 28 doses during a course of therapy. In practice >90% of patients had doses withheld. Doses were withheld for specific toxicities (See “**DOSAGE AND ADMINISTRATION**” section, “**Dose Modifications**” subsection and “**ADVERSE REACTIONS**” section).

Metastatic Renal Cell Cancer

Two hundred fifty-five patients with metastatic renal cell cancer (metastatic RCC) were treated with single agent Proleukin in 7 clinical studies conducted at 21 institutions. Metastatic RCC patients received a median of 20 of 28 scheduled doses of Proleukin.

In the renal cell cancer studies (n=255), objective response was seen in 37 (15%) patients, with 17 (7%) complete and 20 (8%) partial responders (See Table I). The 95% confidence interval for objective response was 11% to 20%. Onset of tumor regression was observed as early as 4 weeks after completion of the first course of treatment, and in some cases, tumor regression continued for up to 12 months after the start of treatment. Responses were observed in both lung and non-lung sites (e.g., liver, lymph node, renal bed occurrences, soft tissue). Responses were also observed in patients with individual bulky lesions and high tumor burden.

TABLE 1: Proleukin Clinical Response Data

	Number of Responding Patients (response rate)	Median Response Duration in Months (range)
**Metastatic RCC**		
CR's	17 (7%)	80+* (7 to 131+)
PR's	20 (8%)	20 (3 to 126+)
PR's + CR's	37 (15%)	54 (3 to 131+)

(+) sign means ongoing

* Median duration not yet observed; a conservative value is presented which represents the minimum median duration of response.

<u>Lack of efficacy with low dose Proleukin regimens</u>

Sixty-five patients with metastatic renal cell cancer were enrolled in a single center, open label, non-randomized trial that sequentially evaluated the safety and anti-tumor activity of two low dose Proleukin regimens. The regimens administered 18 million International Units Proleukin as a single subcutaneous injection, daily for 5 days during week 1; Proleukin was then administered at 9 $x10^6$ International Units days 1-2 and 18 $x10^6$ International Units days 3-5, weekly for an additional 3 weeks (n=40) followed by a 2 week rest or 5 weeks (n=25) followed by a 3 week rest, for a maximum of 3 or 2 treatment cycles, respectively.

These low dose regimens yielded substantially lower and less durable responses than those observed with the approved regimen. Based on the level of activity, these low dose regimens are not effective.

Metastatic Melanoma

Two hundred seventy patients with metastatic melanoma were treated with single agent Proleukin in 8 clinical studies conducted at 22 institutions. Metastatic melanoma patients received a median of 18 of 28 scheduled doses of Proleukin during the first course of therapy. In the metastatic melanoma studies (n=270), objective response was seen in 43 (16%) patients, with 17 (6%) complete and 26 (10%) partial responders (See Table II). The 95% confidence interval for objective response was 12% to 21%. Responses in metastatic melanoma patients were observed in both visceral and non-visceral sites (e.g., lung, liver, lymph node, soft tissue, adrenal, subcutaneous). Responses were also observed in patients with individual bulky lesions and large cumulative tumor burden.

TABLE 2: Proleukin CLINICAL RESPONSE DATA

	**Number of Responding Patients (response rate)**	**Median Response Duration in Months (range)**
**Metastatic Melanoma**		
CR's	17 (6%)	59+* (3 to 122+)
PR's	26 (10%)	6 (1 to 111+)
PR's + CR's	43 (16%)	9 (1 to 122+)

(+) sign means ongoing

* Median duration not yet observed; a conservative value is presented which represents the minimum median duration of response.

INDICATIONS AND USAGE

Proleukin® (aldesleukin) is indicated for the treatment of adults with metastatic renal cell carcinoma (metastatic RCC).

Proleukin is indicated for the treatment of adults with metastatic melanoma.

Careful patient selection is mandatory prior to the administration of Proleukin. See "**CONTRAINDICATIONS**", "**WARNINGS**" and "**PRECAUTIONS**" sections regarding patient screening, including recommended cardiac and pulmonary function tests and laboratory tests.

Evaluation of clinical studies to date reveals that patients with more favorable ECOG performance status (ECOG PS 0) at treatment initiation respond better to Proleukin, with a higher response rate and lower toxicity (See "**CLINICAL PHARMACOLOGY**" section, "**CLINICAL STUDIES**" section and "**ADVERSE REACTIONS**" section). Therefore, selection of patients for treatment should include assessment of performance status.

Experience in patients with ECOG PS >1 is extremely limited.

CONTRAINDICATIONS

Proleukin® (aldesleukin) is contraindicated in patients with a known history of hypersensitivity to interleukin-2 or any component of the Proleukin formulation.

Proleukin is contraindicated in patients with an abnormal thallium stress test or abnormal pulmonary function tests and those with organ allografts. Retreatment with Proleukin is contraindicated in patients who have experienced the following drug-related toxicities while receiving an earlier course of therapy:

- Sustained ventricular tachycardia (≥5 beats)
- Cardiac arrhythmias not controlled or unresponsive to management
- Chest pain with ECG changes, consistent with angina or myocardial infarction
- Cardiac tamponade
- Intubation for >72 hours

- Renal failure requiring dialysis >72 hours
- Coma or toxic psychosis lasting >48 hours
- Repetitive or difficult to control seizures
- Bowel ischemia/perforation
- GI bleeding requiring surgery

WARNINGS

See boxed "**WARNINGS**"

Because of the severe adverse events which generally accompany Proleukin® (aldesleukin) therapy at the recommended dosages, thorough clinical evaluation should be performed to identify patients with significant cardiac, pulmonary, renal, hepatic, or CNS impairment in whom Proleukin is contraindicated. Patients with normal cardiovascular, pulmonary, hepatic, and CNS function may experience serious, life threatening or fatal adverse events. Adverse events are frequent, often serious, and sometimes fatal.

Should adverse events, which require dose modification occur, dosage should be withheld rather than reduced (See "**DOSAGE AND ADMINISTRATION**" section, "**Dose Modifications**" subsection).

Proleukin has been associated with exacerbation of pre-existing or initial presentation of autoimmune disease and inflammatory disorders. Exacerbation of Crohn's disease, scleroderma, thyroiditis, inflammatory arthritis, diabetes mellitus, oculo-bulbar myasthenia gravis, crescentic IgA glomerulonephritis, cholecystitis, cerebral vasculitis, Stevens-Johnson syndrome and bullous pemphigoid, has been reported following treatment with IL-2.

All patients should have thorough evaluation and treatment of CNS metastases and have a negative scan prior to receiving Proleukin therapy. New neurologic signs, symptoms, and anatomic lesions following Proleukin therapy have been reported in patients without evidence of CNS metastases. Clinical manifestations included changes in mental status, speech difficulties, cortical blindness, limb or gait ataxia, hallucinations, agitation, obtundation, and coma. Radiological findings included multiple and, less commonly, single cortical lesions on MRI and evidence of demyelination. Neurologic signs and symptoms associated with Proleukin therapy usually improve after discontinuation of Proleukin therapy; however, there are reports of permanent neurologic defects. One case of possible cerebral vasculitis, responsive to dexamethasone, has been reported. In patients with known seizure disorders, extreme caution should be exercised as Proleukin may cause seizures.

PRECAUTIONS

General

Patients should have normal cardiac, pulmonary, hepatic, and CNS function at the start of therapy. (See "**PRECAUTIONS**" section, "**Laboratory Tests**" subsection). Capillary leak syndrome (CLS) begins immediately after Proleukin® (aldesleukin) treatment starts and is marked by increased capillary permeability to protein and fluids and reduced vascular tone. In most patients, this results in a concomitant drop in mean arterial blood pressure within 2 to 12 hours after the start of treatment. With continued therapy, clinically significant hypotension (defined as systolic blood pressure below 90 mm Hg or a 20 mm Hg drop from baseline systolic pressure) and hypoperfusion will occur. In addition, extravasation of protein and fluids

into the extravascular space will lead to the formation of edema and creation of new effusions.

Medical management of CLS begins with careful monitoring of the patient's fluid and organ perfusion status. This is achieved by frequent determination of blood pressure and pulse, and by monitoring organ function, which includes assessment of mental status and urine output. Hypovolemia is assessed by catheterization and central pressure monitoring.

Flexibility in fluid and pressor management is essential for maintaining organ perfusion and blood pressure. Consequently, extreme caution should be used in treating patients with fixed requirements for large volumes of fluid (e.g., patients with hypercalcemia). Administration of IV fluids, either colloids or crystalloids is recommended for treatment of hypovolemia. Correction of hypovolemia may require large volumes of IV fluids but caution is required because unrestrained fluid administration may exacerbate problems associated with edema formation or effusions. With extravascular fluid accumulation, edema is common and ascites, pleural or pericardial effusions may develop. Management of these events depends on a careful balancing of the effects of fluid shifts so that neither the consequences of hypovolemia (e.g., impaired organ perfusion) nor the consequences of fluid accumulations (e.g., pulmonary edema) exceed the patient's tolerance.

Clinical experience has shown that early administration of dopamine (1 to 5 mcg/kg/min) to patients manifesting capillary leak syndrome, before the onset of hypotension, can help to maintain organ perfusion particularly to the kidney and thus preserve urine output. Weight and urine output should be carefully monitored. If organ perfusion and blood pressure are not sustained by dopamine therapy, clinical investigators have increased the dose of dopamine to 6 to 10 mcg/kg/min or have added phenylephrine hydrochloride (1 to 5 mcg/kg/min) to low dose dopamine (See "**ADVERSE REACTIONS**" section). Prolonged use of pressors, either in combination or as individual agents, at relatively high doses, may be associated with cardiac rhythm disturbances. If there has been excessive weight gain or edema formation, particularly if associated with shortness of breath from pulmonary congestion, use of diuretics, once blood pressure has normalized, has been shown to hasten recovery. **NOTE: Prior to the use of any product mentioned, the physician should refer to the package insert for the respective product.**

Proleukin® (aldesleukin) treatment should be withheld for failure to maintain organ perfusion as demonstrated by altered mental status, reduced urine output, a fall in the systolic blood pressure below 90 mm Hg or onset of cardiac arrhythmias (See "**DOSAGE AND ADMINISTRATION**" section, "**Dose Modifications**" subsection). Recovery from CLS begins soon after cessation of Proleukin therapy. Usually, within a few hours, the blood pressure rises, organ perfusion is restored and reabsorption of extravasated fluid and protein begins.

Kidney and liver function are impaired during Proleukin treatment. Use of concomitant nephrotoxic or hepatotoxic medications may further increase toxicity to the kidney or liver.

Mental status changes including irritability, confusion, or depression which occur while receiving Proleukin may be indicators of bacteremia or early bacterial sepsis, hypoperfusion, occult CNS malignancy, or direct Proleukin-induced CNS toxicity. Alterations in mental status due solely to Proleukin therapy may progress for several days before recovery begins. Rarely, patients have sustained permanent neurologic deficits (See "**PRECAUTIONS**" section "**Drug Interactions**" subsection).

Exacerbation of pre-existing autoimmune disease or initial presentation of autoimmune and inflammatory disorders has been reported following Proleukin alone or in combination with

interferon (See “**PRECAUTIONS**” section “**Drug Interactions**” subsection and “**ADVERSE REACTIONS**” section). Hypothyroidism, sometimes preceded by hyperthyroidism, has been reported following Proleukin treatment. Some of these patients required thyroid replacement therapy. Changes in thyroid function may be a manifestation of autoimmunity. Onset of symptomatic hyperglycemia and/or diabetes mellitus has been reported during Proleukin therapy.

Proleukin enhancement of cellular immune function may increase the risk of allograft rejection in transplant patients.

Serious Manifestations of Eosinophilia

Serious manifestations of eosinophilia involving eosinophilic infiltration of cardiac and pulmonary tissues can occur following Proleukin.

Laboratory Tests

The following clinical evaluations are recommended for all patients, prior to beginning treatment and then daily during drug administration.

- Standard hematologic tests-including CBC, differential and platelet counts
- Blood chemistries-including electrolytes, renal and hepatic function tests
- Chest x-rays

Serum creatinine should be ≤1.5 mg/dL prior to initiation of Proleukin treatment.

All patients should have baseline pulmonary function tests with arterial blood gases. Adequate pulmonary function should be documented (FEV_1 >2 liters or ≥75% of predicted for height and age) prior to initiating therapy.

All patients should be screened with a stress thallium study. Normal ejection fraction and unimpaired wall motion should be documented. If a thallium stress test suggests minor wall motion abnormalities further testing is suggested to exclude significant coronary artery disease.

Daily monitoring during therapy with Proleukin should include vital signs (temperature, pulse, blood pressure, and respiration rate), weight, and fluid intake and output. In a patient with a decreased systolic blood pressure, especially less than 90 mm Hg, constant cardiac rhythm monitoring should be conducted. If an abnormal complex or rhythm is seen, an ECG should be performed. Vital signs in these hypotensive patients should be taken hourly.

During treatment, pulmonary function should be monitored on a regular basis by clinical examination, assessment of vital signs and pulse oximetry. Patients with dyspnea or clinical signs of respiratory impairment (tachypnea or rales) should be further assessed with arterial blood gas determination. These tests are to be repeated as often as clinically indicated.

Cardiac function should be assessed daily by clinical examination and assessment of vital signs. Patients with signs or symptoms of chest pain, murmurs, gallops, irregular rhythm or palpitations should be further assessed with an ECG examination and cardiac enzyme evaluation. Evidence of myocardial injury, including findings compatible with myocardial infarction or myocarditis, has been reported. Ventricular hypokinesia due to myocarditis may be persistent for several months. If there is evidence of cardiac ischemia or congestive heart failure, Proleukin therapy should be held, and a repeat thallium study should be done.

Drug Interactions

Proleukin may affect central nervous function. Therefore, interactions could occur following concomitant administration of psychotropic drugs (e.g., narcotics, analgesics, antiemetics, sedatives, tranquilizers).

Concurrent administration of drugs possessing nephrotoxic (e.g., aminoglycosides, indomethacin), myelotoxic (e.g., cytotoxic chemotherapy), cardiotoxic (e.g., doxorubicin) or hepatotoxic (e.g., methotrexate, asparaginase) effects with Proleukin may increase toxicity in these organ systems. The safety and efficacy of Proleukin in combination with any antineoplastic agents have not been established.

In addition, reduced kidney and liver function secondary to Proleukin treatment may delay elimination of concomitant medications and increase the risk of adverse events from those drugs.

Hypersensitivity reactions have been reported in patients receiving combination regimens containing sequential high dose Proleukin and antineoplastic agents, specifically, dacarbazine, cis-platinum, tamoxifen and interferon-alfa. These reactions consisted of erythema, pruritus, and hypotension and occurred within hours of administration of chemotherapy. These events required medical intervention in some patients.

Myocardial injury, including myocardial infarction, myocarditis, ventricular hypokinesia, and severe rhabdomyolysis appear to be increased in patients receiving Proleukin and interferon-alfa concurrently.

Exacerbation or the initial presentation of a number of autoimmune and inflammatory disorders has been observed following concurrent use of interferon-alfa and Proleukin, including crescentic IgA glomerulonephritis, oculo-bulbar myasthenia gravis, inflammatory arthritis, thyroiditis, bullous pemphigoid, and Stevens-Johnson syndrome.

Although glucocorticoids have been shown to reduce Proleukin-induced side effects including fever, renal insufficiency, hyperbilirubinemia, confusion, and dyspnea, concomitant administration of these agents with Proleukin may reduce the antitumor effectiveness of Proleukin and thus should be avoided.[12]

Beta-blockers and other antihypertensives may potentiate the hypotension seen with Proleukin.

Delayed Adverse Reactions to Iodinated Contrast Media

A review of the literature revealed that 12.6% (range 11-28%) of 501 patients treated with various interleukin-2 containing regimens who were subsequently administered radiographic iodinated contrast media experienced acute, atypical adverse reactions. The onset of symptoms usually occurred within hours (most commonly 1 to 4 hours) following the administration of contrast media. These reactions include fever, chills, nausea, vomiting, pruritus, rash, diarrhea, hypotension, edema, and oliguria. Some clinicians have noted that these reactions resemble the immediate side effects caused by interleukin-2 administration, however the cause of contrast reactions after interleukin-2 therapy is unknown. Most events were reported to occur when contrast media was given within 4 weeks after the last dose of interleukin-2. These events were also reported to occur when contrast media was given several months after interleukin-2 treatment.[13]

Carcinogenesis, Mutagenesis, Impairment of Fertility

There have been no studies conducted assessing the carcinogenic or mutagenic potential of Proleukin.

There have been no studies conducted assessing the effect of Proleukin on fertility. It is recommended that this drug not be administered to fertile persons of either gender not practicing effective contraception.

Pregnancy

Pregnancy Category C.

Proleukin has been shown to have embryolethal effects in rats when given in doses at 27 to 36 times the human dose (scaled by body weight). Significant maternal toxicities were observed in pregnant rats administered Proleukin by IV injection at doses 2.1 to 36 times higher than the human dose during critical period of organogenesis. No evidence of teratogenicity was observed other than that attributed to maternal toxicity. There are no adequate well-controlled studies of Proleukin in pregnant women. Proleukin should be used during pregnancy only if the potential benefit justifies the potential risk to the fetus.

Nursing Mothers

It is not known whether this drug is excreted in human milk. Because many drugs are excreted in human milk and because of the potential for serious adverse reactions in nursing infants from Proleukin, a decision should be made whether to discontinue nursing or to discontinue the drug, taking into account the importance of the drug to the mother.

Pediatric Use

Safety and effectiveness in children under 18 years of age have not been established.

Geriatric Use

There were a small number of patients aged 65 and over in clinical trials of Proleukin; experience is limited to 27 patients, eight with metastatic melanoma and nineteen with metastatic renal cell carcinoma. The response rates were similar in patients 65 years and over as compared to those less than 65 years of age. The median number of courses and the median number of doses per course were similar between older and younger patients.

Proleukin is known to be substantially excreted by the kidney, and the risk of toxic reactions to this drug may be greater in patients with impaired renal function. The pattern of organ system toxicity and the proportion of patients with severe toxicities by organ system were generally similar in patients 65 and older and younger patients. There was a trend, however, towards an increased incidence of severe urogenital toxicities and dyspnea in the older patients.

ADVERSE REACTIONS

The rate of drug-related deaths in the 255 metastatic RCC patients who received single-agent Proleukin® (aldesleukin) was 4% (11/255); the rate of drug-related deaths in the 270 metastatic melanoma patients who received single-agent Proleukin was 2% (6/270).

The following data on common adverse events (reported in greater than 10% of patients, any grade), presented by body system, decreasing frequency and by preferred term (COSTART)

are based on 525 patients (255 with renal cell cancer and 270 with metastatic melanoma) treated with the recommended infusion dosing regimen.

TABLE 3: ADVERSE EVENTS OCCURRING IN ≥10% OF PATIENTS (n=525)

Body System	% Patients	Body System	% Patients
**Body as a Whole**		**Metabolic and Nutritional Disorders**	
Chills	52	Bilirubinemia	40
Fever	29	Creatinine increase	33
Malaise	27	Peripheral edema	28
Asthenia	23	SGOT increase	23
Infection	13	Weight gain	16
Pain	12	Edema	15
Abdominal pain	11	Acidosis	12
Abdomen enlarged	10	Hypomagnesemia	12
**Cardiovascular**		Hypocalcemia	11
Hypotension	71	Alkaline phosphatase increase	10
Tachycardia	23	**Nervous**	
Vasodilation	13	Confusion	34
Supraventricular tachycardia	12	Somnolence	22
Cardiovascular disorder[a]	11	Anxiety	12
Arrhythmia	10	Dizziness	11
**Digestive**		**Respiratory**	
Diarrhea	67	Dyspnea	43
Vomiting	50	Lung disorder[b]	24
Nausea	35	Respiratory disorder[c]	11
Stomatitis	22	Cough increase	11
Anorexia	20	Rhinitis	10
Nausea and vomiting	19	**Skin and Appendages**	
**Hemic and Lymphatic**		Rash	42
Thrombocytopenia	37	Pruritus	24
Anemia	29	Exfoliative dermatitis	18
Leukopenia	16	**Urogenital**	
		Oliguria	63

[a] Cardiovascular disorder: fluctuations in blood pressure, asymptomatic ECG changes, CHF.

[b] Lung disorder: physical findings associated with pulmonary congestion, rales, rhonchi.

[c] Respiratory disorder: ARDS, CXR infiltrates, unspecified pulmonary changes.

The following data on life-threatening adverse events (reported in greater than 1% of patients, grade 4), presented by body system, and by preferred term (COSTART) are based on 525 patients (255 with renal cell cancer and 270 with metastatic melanoma) treated with the recommended infusion dosing regimen.

TABLE 4: LIFE-THREATENING (GRADE 4) ADVERSE EVENTS (n= 525)

Body System	# (%) Patients	Body System	# (%) Patients
**Body as a Whole**		**Metabolic and Nutritional Disorders**	
Fever	5 (1%)	Bilirubinemia	13 (2%)
Infection	7 (1%)	Creatinine increase	5 (1%)
Sepsis	6 (1%)	SGOT increase	3 (1%)
**Cardiovascular**		Acidosis	4 (1%)
Hypotension	15 (3%)	**Nervous**	
Supraventricular tachycardia	3 (1%)	Confusion	5 (1%)
Cardiovascular disorder[a]	7 (1%)	Stupor	3 (1%)
Myocardial infarct	7 (1%)	Coma	8 (2%)
Ventricular tachycardia	5 (1%)	Psychosis	7 (1%)
Cardiac arrest	4 (1%)	**Respiratory**	
**Digestive**		Dyspnea	5 (1%)
Diarrhea	10 (2%)	Respiratory disorder[c]	14 (3%)
Vomiting	7 (1%)	Apnea	5 (1%)
**Hemic and Lymphatic**		**Urogenital**	
Thrombocytopenia	5 (1%)	Oliguria	33 (6%)
Coagulation disorder[b]	4 (1%)	Anuria	25 (5%)
		Acute kidney failure	3 (1%)

[a] Cardiovascular disorder: fluctuations in blood pressure.

[b] Coagulation disorder: intravascular coagulopathy.

[c] Respiratory disorder: ARDS, respiratory failure, intubation.

The following life-threatening (grade 4) events were reported by <1% of the 525 patients: hypothermia; shock; bradycardia; ventricular extrasystoles; myocardial ischemia; syncope; hemorrhage; atrial arrhythmia; phlebitis; AV block second degree; endocarditis; pericardial

effusion; peripheral gangrene; thrombosis; coronary artery disorder; stomatitis; nausea and vomiting; liver function tests abnormal; gastrointestinal hemorrhage; hematemesis; bloody diarrhea; gastrointestinal disorder; intestinal perforation; pancreatitis; anemia; leukopenia; leukocytosis; hypocalcemia; alkaline phosphatase increase; BUN increase; hyperuricemia; NPN increase; respiratory acidosis; somnolence; agitation; neuropathy; paranoid reaction; convulsion; grand mal convulsion; delirium; asthma, lung edema; hyperventilation; hypoxia; hemoptysis; hypoventilation; pneumothorax; mydriasis; pupillary disorder; kidney function abnormal; kidney failure; acute tubular necrosis.

In an additional population of greater than 1,800 patients treated with Proleukin-based regimens using a variety of doses and schedules (e.g., subcutaneous, continuous infusion, administration with LAK cells) the following serious adverse events were reported: duodenal ulceration; bowel necrosis; myocarditis; supraventricular tachycardia; permanent or transient blindness secondary to optic neuritis; transient ischemic attacks; meningitis; cerebral edema; pericarditis; allergic interstitial nephritis; tracheo-esophageal fistula.

In the same clinical population, the following fatal events each occurred with a frequency of <1%: malignant hyperthermia; cardiac arrest; myocardial infarction; pulmonary emboli; stroke; intestinal perforation; liver or renal failure; severe depression leading to suicide; pulmonary edema; respiratory arrest; respiratory failure. In patients with both metastatic RCC and metastatic melanoma, those with ECOG PS of 1 or higher had a higher treatment-related mortality and serious adverse events.

Most adverse reactions are self-limiting and, usually, but not invariably, reverse or improve within 2 or 3 days of discontinuation of therapy. Examples of adverse reactions with permanent sequelae include: myocardial infarction, bowel perforation/infarction, and gangrene.

Immunogenicity

Serum samples from patients in the clinical studies were tested by enzyme-linked immunosorbent assay (ELISA) for anti-aldesleukin antibodies. Low titers of anti-aldesleukin antibodies were detected in 57 of 77 (74%) patients with metastatic renal cell carcinoma treated with an every 8-hour PROLEUKIN regimen and in 33 of 50 (66%) patients with metastatic melanoma treated with a variety of intravenous regimens. In a separate study, the effect of immunogenicity on the pharmacokinetics of aldesleukin was evaluated in 13 patients. Following the first cycle of therapy, comparing the geometric mean aldesleukin exposure (AUC) Day 15 to Day 1, there was an average 68% increase in 11 patients who developed anti-aldesleukin antibodies and no change was observed in the antibody-negative patients (n=2). Overall, neutralizing antibodies were detected in 1 patient. The impact of antialdesleukin antibody formation on clinical efficacy and safety of PROLEUKIN is unknown.

Immunogenicity assay results are highly dependent on several factors including assay sensitivity and specificity, assay methodology, sample handling, timing of sample collection, concomitant medications, and underlying disease. For these reasons, comparison of incidence of antibodies to PROLEUKIN with the incidence of antibodies to other products may be misleading.

Post Marketing Experience

The following adverse reactions have been identified during post-approval use of Proleukin. Because these reactions are reported voluntarily from a population of uncertain size, it is not

always possible to reliably estimate their frequency or establish a causal relationship to drug exposure.

- Blood and lymphatic system: neutropenia, febrile neutropenia, eosinophilia, lymphocytopenia
- Cardiac: cardiomyopathy, cardiac tamponade
- Endocrine: hyperthyroidism
- Gastrointestinal: gastritis, intestinal obstruction, colitis
- General and administration site conditions: injection site necrosis
- Hepatobiliary: hepatitis, hepatosplenomegaly, cholecystitis
- Immune system: anaphylaxis, angioedema, urticaria
- Infections and infestations: pneumonia (bacterial, fungal, viral), fatal endocarditis, cellulitis
- Musculoskeletal and connective tissue: myopathy, myositis, rhabdomyolysis
- Nervous system: cerebral lesions, encephalopathy, extrapyramidal syndrome, neuralgia, neuritis, demyelinating neuropathy
- Psychiatric: insomnia
- Vascular: hypertension, fatal subdural and subarachnoid hemorrhage, cerebral hemorrhage, retroperitoneal hemorrhage

Exacerbation or initial presentation of a number of autoimmune and inflammatory disorders have been reported (See “**WARNINGS**” section, “**PRECAUTIONS**” section, “**Drug Interactions**” subsection). Persistent but nonprogressive vitiligo has been observed in malignant melanoma patients treated with interleukin-2. Synergistic, additive and novel toxicities have been reported with Proleukin used in combination with other drugs. Novel toxicities include delayed adverse reactions to iodinated contrast media and hypersensitivity reactions to antineoplastic agents (See “**PRECAUTIONS**” section, “**Drug Interactions**” subsection).

Experience has shown the following concomitant medications to be useful in the management of patients on Proleukin therapy: a) standard antipyretic therapy, including nonsteroidal anti-inflammatories (NSAIDs), started immediately prior to Proleukin to reduce fever. Renal function should be monitored as some NSAIDs may cause synergistic nephrotoxicity; b) meperidine used to control the rigors associated with fever; c) H_2 antagonists given for prophylaxis of gastrointestinal irritation and bleeding; d) antiemetics and antidiarrheals used as needed to treat other gastrointestinal side effects. Generally these medications were discontinued 12 hours after the last dose of Proleukin.

Patients with indwelling central lines have a higher risk of infection with gram positive organisms.[9-11] A reduced incidence of staphylococcal infections in Proleukin studies has been associated with the use of antibiotic prophylaxis which includes the use of oxacillin, nafcillin, ciprofloxacin, or vancomycin. Hydroxyzine or diphenhydramine has been used to control symptoms from pruritic rashes and continued until resolution of pruritus. Topical creams and ointments should be applied as needed for skin manifestations. Preparations containing a

steroid (e.g., hydrocortisone) should be avoided. **NOTE: Prior to the use of any product mentioned, the physician should refer to the package insert for the respective product.**

OVERDOSAGE

Side effects following the use of Proleukin® (aldesleukin) appear to be dose-related. Exceeding the recommended dose has been associated with a more rapid onset of expected dose-limiting toxicities. Symptoms which persist after cessation of Proleukin should be monitored and treated supportively. Life-threatening toxicities may be ameliorated by the intravenous administration of dexamethasone, which may also result in loss of the therapeutic effects of Proleukin.[12] **NOTE: Prior to the use of dexamethasone, the physician should refer to the package insert for this product.**

DOSAGE AND ADMINISTRATION

The recommended Proleukin® (aldesleukin) treatment regimen is administered by a 15-minute intravenous infusion every 8 hours. Before initiating treatment, carefully review the "**INDICATIONS AND USAGE**", "**CONTRAINDICATIONS**", "**WARNINGS**", "**PRECAUTIONS**", and "**ADVERSE REACTIONS**" sections, particularly regarding patient selection, possible serious adverse events, patient monitoring and withholding dosage. The following schedule has been used to treat adult patients with metastatic renal cell carcinoma (metastatic RCC) or metastatic melanoma. Each course of treatment consists of two 5-day treatment cycles separated by a rest period.

> 600,000 International Units/kg (0.037 mg/kg) dose administered every 8 hours by a 15-minute intravenous infusion for a maximum of 14 doses. Following 9 days of rest, the schedule is repeated for another 14 doses, for a maximum of 28 doses per course, as tolerated. During clinical trials, doses were frequently withheld for toxicity (See "**CLINICAL STUDIES**" section and "**Dose Modifications**" subsection). Metastatic RCC patients treated with this schedule received a median of 20 of the 28 doses during the first course of therapy. Metastatic melanoma patients received a median of 18 doses during the first course of therapy.

Retreatment

Patients should be evaluated for response approximately 4 weeks after completion of a course of therapy and again immediately prior to the scheduled start of the next treatment course. Additional courses of treatment should be given to patients only if there is some tumor shrinkage following the last course and retreatment is not contraindicated (See "**CONTRAINDICATIONS**" section). Each treatment course should be separated by a rest period of at least 7 weeks from the date of hospital discharge.

Dose Modifications

Dose modification for toxicity should be accomplished by withholding or interrupting a dose rather than reducing the dose to be given. Decisions to stop, hold, or restart Proleukin therapy must be made after a global assessment of the patient. With this in mind, the following guidelines should be used:

Retreatment with Proleukin is contraindicated in patients who have experienced the following toxicities:

Body System

Cardiovascular	Sustained ventricular tachycardia (≥5 beats)
	Cardiac rhythm disturbances not controlled or unresponsive to management
	Chest pain with ECG changes, consistent with angina or myocardial infarction
	Cardiac tamponade
Respiratory	Intubation for >72 hours
Urogenital	Renal failure requiring dialysis >72 hours
Nervous	Coma or toxic psychosis lasting >48 hours
	Repetitive or difficult to control seizures
Digestive	Bowel ischemia/perforation
	GI bleeding requiring surgery

Doses should be held and restarted according to the following:

Body System	Hold dose for	Subsequent doses may be given if
Cardiovascular	Atrial fibrillation, supraventricular tachycardia or bradycardia that requires treatment or is recurrent or persistent	Patient is asymptomatic with full recovery to normal sinus rhythm
	Systolic bp <90 mm Hg with increasing requirements for pressors	Systolic bp ≥90 mm Hg and stable or improving requirements for pressors
	Any ECG change consistent with MI, ischemia or myocarditis with or without chest pain; suspicion of cardiac ischemia	Patient is asymptomatic, MI and myocarditis have been ruled out, clinical suspicion of angina is low; there is no evidence of ventricular hypokinesia
Respiratory	O_2 saturation <90%	O_2 saturation >90%
Nervous	Mental status changes, including moderate confusion or agitation	Mental status changes completely resolved
Body as a Whole	Sepsis syndrome, patient is clinically unstable	Sepsis syndrome has resolved, patient is clinically stable, infection is under treatment
Urogenital	Serum creatinine >4.5 mg/dL or a serum creatinine of ≥4 mg/dL in the presence of severe volume overload, acidosis, or hyperkalemia	Serum creatinine <4 mg/dL and fluid and electrolyte status is stable
	Persistent oliguria, urine output of <10 mL/hour for 16 to 24 hours with rising serum creatinine	Urine output >10 mL/hour with a decrease of serum creatinine >1.5 mg/dL or normalization of serum creatinine
Digestive	Signs of hepatic failure including encephalopathy, increasing ascites, liver pain, hypoglycemia	All signs of hepatic failure have resolved*
	Stool guaiac repeatedly >3-4+	Stool guaiac negative
Skin	Bullous dermatitis or marked worsening of pre-existing skin condition, avoid	Resolution of all signs of bullous dermatitis

	topical steroid therapy	

* Discontinue all further treatment for that course. A new course of treatment, if warranted, should be initiated no sooner than 7 weeks after cessation of adverse event and hospital discharge.

Reconstitution and Dilution Directions: Reconstitution and dilution procedures other than those recommended may alter the delivery and/or pharmacology of Proleukin and thus should be avoided.

1. Proleukin® (aldesleukin) is a sterile, white to off-white, preservative-free, lyophilized powder suitable for IV infusion upon reconstitution and dilution. **EACH VIAL CONTAINS 22 MILLION International Units (1.3 mg) OF PROLEUKIN AND SHOULD BE RECONSTITUTED ASEPTICALLY WITH 1.2 mL OF STERILE WATER FOR INJECTION, USP. WHEN RECONSTITUTED AS DIRECTED, EACH mL CONTAINS 18 MILLION International Units (1.1 mg) OF PROLEUKIN.** The resulting solution should be a clear, colorless to slightly yellow liquid. The vial is for single-use only and any unused portion should be discarded.
2. During reconstitution, the Sterile Water for Injection, USP should be directed at the side of the vial and the contents gently swirled to avoid excess foaming. DO NOT SHAKE.
3. The dose of Proleukin, reconstituted with Sterile Water for Injection, USP (without preservative) should be diluted aseptically in 50 mL of 5% Dextrose Injection, USP (D5W) and infused over a 15-minute period.

   In cases where the total dose of Proleukin is 1.5 mg or less (e.g., a patient with a body weight of less than 40 kilograms), the dose of Proleukin should be diluted in a smaller volume of D5W. Concentrations of Proleukin below 0.03 mg/mL and above 0.07 mg/mL have shown increased variability in drug delivery. Dilution and delivery of Proleukin outside of this concentration range should be avoided.
4. Glass bottles and plastic (polyvinyl chloride) bags have been used in clinical trials with comparable results. It is recommended that plastic bags be used as the dilution container since experimental studies suggest that use of plastic containers results in more consistent drug delivery. **In-line filters should not be used when administering Proleukin.**
5. Before and after reconstitution and dilution, store in a refrigerator at 2° to 8°C (36° to 46°F). Do not freeze. Administer Proleukin within 48 hours of reconstitution. The solution should be brought to room temperature prior to infusion in the patient.
6. Reconstitution or dilution with Bacteriostatic Water for Injection, USP, or 0.9% Sodium Chloride Injection, USP should be avoided because of increased aggregation. Proleukin should not be coadministered with other drugs in the same container.
7. Parenteral drug products should be inspected visually for particulate matter and discoloration prior to administration, whenever solution and container permit.

HOW SUPPLIED

Proleukin® (aldesleukin) is supplied in individually boxed single-use vials. Each vial contains 22 million International Units of Proleukin. Discard unused portion.

NDC 65483-116-07   Individually boxed single-use vial

Store vials of lyophilized Proleukin in a refrigerator at 2° to 8°C (36° to 46°F). PROTECT FROM LIGHT. Store in carton until time of use.

Reconstituted or diluted Proleukin is stable for up to 48 hours at refrigerated and room temperatures, 2° to 25°C (36° to 77°F). However, since this product contains no preservative, the reconstituted and diluted solutions should be stored in the refrigerator.

Do not use beyond the expiration date printed on the vial. **NOTE:** This product contains no preservative.

Rx Only

REFERENCES

1. Doyle MV, Lee MT, Fong S. Comparison of the biological activities of human recombinant interleukin-2^{125} and native interleukin-2. J *Biol Response Mod* 1985; **4**:96-109.
2. Ralph P, Nakoinz I, Doyle M, et al. Human B and T lymphocyte stimulating properties of interleukin-2 (IL-2) muteins. In: *Immune Regulation By Characterized Polypeptides*. Alan R. Liss, Inc. 1987; 453-62.
3. Winkelhake JL and Gauny SS. Human recombinant interleukin-2 as an experimental therapeutic. *Pharmacol Rev* 1990; **42**:1-28.
4. Rosenberg SA, Mule JJ, Spiess PJ, et al. Regression of established pulmonary metastases and subcutaneous tumor mediated by the systemic administration of high-dose recombinant interleukin-2. *J Exp Med* 1985; **161**:1169-88.
5. Konrad MW, Hemstreet G, Hersh EM, et al. Pharmacokinetics of recombinant interleukin-2 in humans. *Cancer Res* 1990; **50**:2009-17.
6. Donohue JH and Rosenberg SA. The fate of interleukin-2 after *in vivo* administration. *J Immunol* 1983; **130**:2203-8.
7. Koths K, Halenbeck R. Pharmacokinetic studies on ^{35}S-labeled recombinant interleukin-2 in mice. In: Sorg C and Schimpl A, eds. *Cellular and Molecular Biology of Lymphokines*. Academic Press: Orlando, FL, 1985;779.
8. Gibbons JA, Luo ZP, Hansen ER, et al. Quantitation of the renal clearance of interleukin-2 using nephrectomized and ureter ligated rats. *J Pharmacol Exp Ther* 1995; **272**: 119-125.
9. Bock SN, Lee RE, Fisher B, et al. A prospective randomized trial evaluating prophylactic antibiotics to prevent triple-lumen catheter-related sepsis in patients treated with immunotherapy. *J Clin Oncol* 1990; 8:161-69.
10. Hartman LC, Urba WJ, Steis RG, et al. Use of prophylactic antibiotics for prevention of intravascular catheter-related infections in interleukin-2-treated patients. *J Natl Cancer Inst* 1989; 81:1190-93.
11. Snydman DR, Sullivan B, Gill M, et al. Nosocomial sepsis associated with interleukin-2. *Ann Intern Med* 1990; 112:102-07.
12. Mier JW, Vachino G, Klempner MS, et al. Inhibition of interleukin-2-induced tumor necrosis factor release by dexamethasone: Prevention of an acquired neutrophil chemotaxis defect and differential suppression of interleukin-2 associated side effects. *Blood* 1990; 76:1933-40.

13. Choyke PL, Miller DL, Lotze MT, et al. Delayed reactions to contrast media after interleukin-2 immunotherapy. *Radiology* 1992; 183:111-114.

Manufactured by:

Prometheus Laboratories Inc.

San Diego, CA 92121

U.S. License No. 1848

At

Bayer HealthCare Pharmaceuticals

Emeryville, CA 94608

For additional information, contact Prometheus Laboratories Inc. 1-877-PROLEUKIN (1-877-776-5385)

PROLEUKIN is a registered trademark of Novartis Vaccines and Diagnostics, Inc.

© 2011 Prometheus Laboratories Inc.

PROMETHEUS®
Therapeutics & Diagnostics

REV: July 2012 PR001E
