# Supplementary material for: IL-2 based cancer immunotherapies: an evolving paradigm
Source: Front Immunol. 2024 Jul 24;15:1433989. doi: 10.3389/fimmu.2024.1433989 (PMC11303236; doi:10.3389/fimmu.2024.1433989)
Supplement: Supplementary file 2 [file Table_1.docx]

| **Drug Formulation**  **Table S1: Novel IL-2 formulations in development for cancer treatment** | **Design/Structure** | **Preclinical models/ ClinicalTrials.gov Identifier/ Phase/ Status/ Beginning of Patient Enrollment** | **Outcome** |
| --- | --- | --- | --- |
| ***Unbiased IL-2*** | | | |
| **L19-IL2 (Darleukin)** | Anti–extra domain B (EDB) mAb (L19) specifically targeting EDB, a tumor angiogenesis marker found in the newly formed blood vessels of most tumors is fused to WT IL-2 | NCT01058538, Phase I/II, completed (Nov 2009) | Manageable and reversible toxicities;  SD in 17/33 patients (51%) and 15/18 with mRCC (83%) after two cycles, Median PFS of RCC patients in the expansion phase of the study was 8 months (1.5-30.5) [1] |
|  |  | NCT01253096, Phase II, completed (Sept 2013) | No severe adverse events;  25% CR, 54% ORR in index lesions [2] |
| **Daromun** | Combination of L19-IL2 (darleukin) and L19-TNFα (fibromun) where L19 is fused to hTNF-α (L19-TNFα) | NCT02076633, Phase II, completed (May 2015) | In 20 patients with unresectable stage IIIC and IVM1a metastatic melanoma: Grade 1 and 2 adverse events;  55% ORR [1 patient with CR (5%), 10 (50%) with PR]; CR in 7/13 (53.8%) non-injected lesions [3] |
|  |  | NCT02938299 (recruiting, July 2016) and NCT03567889 (recruiting, Sept 2018): open label Phase III trials in patients with stage IIIB and IIIC melanoma, Recruiting | For NCT02938299 trial, Benign and manageable TRAEs with Grade 3 adverse events in 24.8% patients;  Study met the primary end point of recurrence free survival (RFS) with statistically significant and clinically meaningful improvement in RFS in Daromun + surgery treated group versus surgery alone group [4] |
| **APN-301 (hu14.18-IL2)** | Anti-GD2 (disialoganglioside-a cell membrane molecule predominantly expressed in neuroectodermal tumors) antibody fused to WT IL-2 | NCT00109863, Phase II, Completed (May 2005) | Reversible toxicities - 2 patients developing grade 3 hypotension and one patient showing grade 2 renal insufficiency with oliguria;  One of 14 MM patients showed PR (response rate of 7.1%) and 4 patients had SD, PR and SD responses lasted 3–4 months [5] |
| **WTX-124** | Conditionally activated IL-2 prodrug, containing an inactivation domain and a half-life extension domain fused to a fully active IL-2 by protease-cleavable linkers | NCT05479812, Phase I (July 2022), Recruiting | In the monotherapy dose escalation arm, 16 patients WTX-124 was generally well tolerated with no cases of CLS, no DLTs and no treatment discontinuations due to adverse events reported so far;  Antitumor activity observed with an unconfirmed PR in a MM patient;  Half-life of 1.9-5.8 days, expansion and activation of CD8+ T cell and NK cell expansion and activation in the tumor [6] |
| **TILT-123** | Oncolytic adenovirus (Ad5/3-E2F-d24-hTNF-IRES-hIL2;) expressing two potent cytokines, TNF-α and IL-2 to stimulate T-cell infiltration into the tumor | TUNIMO (NCT04695327), a single-arm, multicenter phase I dose-escalation trial | Most frequent TRAEs included fever (16.7%), chills (13.0%), and fatigue (9.3%)  Systemic immune activation and T cell infiltration into the tumors  Tumor size reductions were observed in both injected and non-injected lesions [7, 8] |
| ***Non-alpha/ IL-2Rβ/ intermediate-affinity IL-2R biased IL-2v*** | | | |
| **ProIL-2/sumIL2-Fc** | IL-2v with IL-2Rβ linked to a Fc protein via a matrix metalloproteinases (MMPs) cleavable linker which is cleaved and activated by these tumor-associated proteases. This cleaved free form has enhanced binding to IL2Rβ and reduced IL2Rα binding | Mouse models of MC38, B16, CT26, and 4T1 | Reduced IL-2 toxicity and mortality without compromising antitumor efficacy;  Intra-tumoral activation and expansion of antigen-specific CD8+ T cells [9] |
| **INBRX-120** | Low-affinity IL-2 connected *via* an effector-silenced Fc domain to two high affinity CD8α-targeted single domain antibodies (sdAbs) | Syngeneic tumor models of MC38 and CT26 | Favorable PK and safety profile in NHP models;  Robust *in vivo* anti-tumor efficacy in murine models [10] |
| **ALKS-4230 (Nemvaleukin Alfa)** | Engineered fusion protein of IL-2 fused with the extracellular domain of IL-2Rα, selectively activating effector lymphocytes expressing IL-2Rβγc and inhibits the interaction with IL-2Rα | ARTISTRY-1 trial: NCT02799095, Phase I/II, Administered i.v. as monotherapy and in combination with pembrolizumab, Not Recruiting | TEAEs included hypotension, LFT elevation, pyrexia and chills in more than half of the patients.  Monotherapy group: durable antitumor activity, in RCC [ORR, 18.2% (4/22)] and in melanoma [ORR, 8.7% (4/46)], with 2 PRs (1 unconfirmed) in 30 patients with cutaneous melanoma (ORR, 6.7%) and 2 PRs (1 unconfirmed) in 6 patients with mucosal melanoma (ORR, 33.3%) [11]  Combination with pembrolizumab group: durable antitumor activity [ORR, 16.1% (22/137)]; disease control rate (DCR), 59.9%], in platinum-resistant ovarian cancer [PROC; ORR, 28.6% (4/14); DCR, 71.4%], with 2 complete responses and 2 PRs (1 unconfirmed) in 14 patients [11] |
|  |  | ARTISTRY-3 trial: NCT04592653, Phase I/II, less frequent i.v. dosing as monotherapy and in combination with Pembrolizumab, Recruiting (Sept 2020) | _ |
|  |  | ARTISTRY-6 trial: NCT04830124, Phase II, Monotherapy in patients with advanced cutaneous melanoma or advanced mucosal melanoma who have previously received anti-PD-(L)-1 therapy, Recruiting (May 2021) | _ |
| **MDNA11** | IL-2v with a 30-fold increased affinity for IL-2Rβ over native IL-2 and no binding to IL-2Rα. Fused to human albumin for half-life extension. | NCT05086692, Phase I/II trial as monotherapy and in combination with checkpoint inhibitor, Recruiting (Aug 2021) | No DLTs, half-life of less than 8 hr;  Two durable PRs and seven SD;  Strong expansion of total T cells including activated CD25+CD8+ T cells [12] |
| **NKTR-214 (BEMPEG)** | PEGylated IL-2 with limited IL-2Rα binding and biased towards intermediate affinity IL-2Rβγc, expressed on Teff and NK cells | First in-human multicenter Phase I study of BEMPEG monotherapy | Well tolerated, grade 3 TRAEs in 21.4% patients, no grade 4 TRAEs, CLS associated TRAEs like hypotension, flu-like symptoms, pyrexia and lymphopenia;  Some disease stabilization observed, but overall, no single-agent activity;  Increased number and activation status of immune cells in periphery, limited Treg expansion in tumor biopsies, engagement of IL-2R pathway [13] |
|  |  | NCT02983045, phase I/II PIVOT-02 trial, Combination of BEMPEG plus NIVO, Completed (Apr 2022) | Durable responses in MM patients undergoing combination treatment.  ORR of 53% (n = 20/38), CR of 34% (n = 13/38), 47.4% (18 of 38 patients) complete clearance of target lesions, median PFS was 30.9 months. with a 24-month OS rate. of 77% [12]  Similar safety and encouraging efficacy in other cancer types including RCC, TNBC, UC, and NSCLC [14, 15, 16, 17] |
|  |  | Phase III study, Not recruiting  NCT03635983 (PIVOT IO 001) -  BEMPEG plus NIVO versus NIVO monotherapy in MM patients | Failed its primary endpoints of ORR, PFS and OS.  ORR for BEMPEG plus NIVO was 27.7% versus 36.0% with NIVO alone. CRs occurred in 8.1% of the patients in the BEMPEG plus NIVO arm and in 12.5% in the NIVO arm.  The median OS was 29.67 months with BEMPEG plus NIVO versus 28.88 months with NIVO alone.  Higher incidence of adverse events in BEMPEG plus NIVO group versus NIVO alone group [18, 19] |
|  |  | Phase III study, Not recruiting  NCT03729245 (PIVOT-09) - BEMPEG plus NIVO versus investigator’s choice of sunitinib or cabozantinib (TKI) for advanced RCC patients | Failed its primary endpoints of ORR, PFS, and OS  The ORR was 23.0% for BEMPEG plus NIVO and 30.6% for the TKI arm.  The median OS was 29.0 months for BEMPEG plus NIVO and not reached for the TKI arm at a median duration of follow-up of 15.5 months [20] |
| **THOR-707 (SAR444245)** | PEGylated recombinant non-alpha IL-2v | NCT04009681 ((HAMMER), Phase I/II (June 2019), as monotherapy, or combined with pembrolizumab | No dose-limiting toxicities, TRAEs included pyrexia, chills, lymphocyte decrease  Monotherapy:  1 PR (HNSCC) [21]  No anti-drug antibodies reported, increase in CD8+ T cells, and NK cells in Cycle 1 by a median (range) respectively of 3.1 (1.04 - 5.91) and 7.93 (1.71 - 26.85) maintained until the next cycle, no significant increases in IL-5, CD4+ Tregs or eosinophil counts. [21, 22] |
| **Simlukafusp alfa (FAP-IL2v)** | IL-2Rβγc biased IL-2v fused to an antibody against fibroblast activation protein α (FAP) | Phase I clinical trials in FAP positive solid tumors as a single agent and in combination trastuzumab or cetuximab (NCT02627274),  Combination with Pembrolizumab (NCT03875079),  Combination with atezolizumab with/without bevacizumab (NCT03063762),  All trials completed | _ |
| **XTX202** | IL-2v fused to a masking domain as well as to a half-life extension domain via a protease-cleavable linker, after unmasking by matrix metalloproteases active in the tumor microenvironment, XTX202 binds to the IL-2 receptor β and γc subunits | Syngeneic mouse tumor models | Similar efficacy to aldesleukin without peripheral immune activation, weight loss and lung edema [23] |
|  |  | NCT05052268, Phase I/II trial, Recruiting (Jan 2022) | _ |
| **NL-201** | PEGylated IL-2 mimic that binds to the intermediate IL-2Rβγc receptor with higher affinity than IL-2 but completely lacks binding to IL-2Rα | Several murine tumor models including CT26, MC38, EMT6 and LL/2 | Preclinical efficacy, stimulated sustained, dose dependent CD8+ T and NK cell proliferation [24] |
|  |  | NCT04659629, Phase I, as monotherapy and in combination with Pembrolizumab, Not recruiting/ discontinued | _ |
| **RO7284755, Eciskafusp alfa** | IL-2Rβγc biased IL-2v used to antiPD1-IL2v with WT affinity for IL-2Rβ | NCT04303858, Phase I trial as monotherapy and in combination with atezolizumab, Recruiting (May 2020) | _ |
| **BPT331** | IL-2Rβγc biased IL-2v fused to antiPD1-IL2v with enhanced affinity for IL-2Rβ | _ | _ |
| **AB248** | IL-2Rβγc biased IL-2v fused to antiCD8-IL2v with reduced affinity for IL-2Rβ | NCT05653882, Phase Ia/Ib trial, as a monotherapy and in combination with Keytruda, Recruiting (Jan 2023) | _ |
| **CUE-101** | Fc fusion protein of a HLA complex, a human papillomavirus 16 (HPV16) E7 peptide epitope, reduced affinity human IL-2 molecules, and an effector attenuated human IgG1 Fc domain | NCT04852328, Phase II trial for the treatment of naïve, HLA-A*0201 positive patients with newly diagnosed, locally advanced HPV16+ oropharyngeal squamous-cell carcinoma (OPSCC), Recruiting (Dec 2021) | _ |
| **Cergutuzumab amunaleukin (CEA-IL2v)** | IL-2Rβγc biased IL-2v fused to an antibody against CEA | Murine tumor model | Preferential expansion of CD8+ T cells, NK cells and γδ-T cells in the tumor, blood and lymphoid tissues in murine tumor models [25, 26] |
|  |  | NCT02004106, Phase I trial as monotherapy and NCT02350673, in combination with atezolizumab; completed (Aug 2016 and Dec 2019 respectively) | _ |
| ***IL-2Rα biased IL-2v*** | | | |
| **BAY 50-4798** | N88R mutation resulting in reduced IL-2Rβ binding, bias towards IL-2Rα, no half-life extension domain leading to short-half life of 2 hr and was dosed every 8 hr | Phase I trial, conducted in early 2000's | Efficacy and toxicity of BAY 50-4798 was comparable to Aldesleukin [27, 28] |
| **NHS-IL2LT (Selectikine)** | IL-2v carrying a D20T mutation fused to the c-terminus of a human IgG antibody binding DNA–histone complexes that are present in the necrotic core of tumors | Evaluated in two Phase I clinical trials as monotherapy and in combination with cyclophosphamide (NCT01032681) and radiotherapy (NCT00879866); both trials now completed | No severe cardiovascular side-effects, dose-limiting toxicity was grade 3 skin rash, no objective tumor responses [29] |
|  |  | Phase II study (NCT01973608) terminated early |  |
| **IBI363** | Fusion protein of IL-2Rα biased IL-2s and anti-PD-1 antibody | NCT05290597, Phase I trial, Recruiting (Aug 2022) | _ |
| **Ky1043** | Fusion proteins of IL-2Rα biased IL-2s and an anti-PD-L1 antibody | Tested in mouse tumor models | Preferentially expansion of TCF1+ PD-1+ precursor exhausted CD8+ T cells (Tpex), increased CD8/Treg ratio in the tumor, decreased CD8/Treg ratio in the periphery [30] |
| **STK-012** | PEGylated IL-2v with reduced IL-2Rγc binding and bias towards IL-2Rα expressing cells such as tumor antigen activated T cells | NCT05098132, Phase Ia/Ib trial as monotherapy and in combination with pembrolizumab, Recruiting (Jan 2022) | Half-life of 4 days;  Well tolerated, without DLTs at doses up to 3 mg every three weeks. Adverse events included rash, fatigue, nausea and diarrhea, but patients very rarely had adverse events associated with CLS, and had no IL-2 related lymphopenia;  Three in 31 patients had a confirmed PR (NSCLC, RCC, head and neck cancer, one each) with several patients having durable SD [31] |
| ***IL-2 in combination with ACT*** | | | |
| **Lifileucel (LN-144)** | ACT with autologous TILs in combination with IL-2 | NCT02360579, Phase II trial for treatment of melanoma patients, Active not recruiting | Thrombocytopenia (76.9%), anemia (50.0%), febrile neutropenia 41.7%) were the most common grade 3/4 TRAEs;  ORR (31.4%), with 8 CRs and 40 PRs, 41.7% of responses maintained for 18 months or more; median OS and PFS were 13.9 months and 4.1 months [32] |
|  |  | NCT05607095, Phase I trial for metastatic uveal melanoma, Recruiting (Nov 2022) | _ |
|  |  | NCT05176470, Phase I trial with Lifileucel and Pembrolizumab for treatment of patients with locally advanced (Stage IIB-D)/Metastatic (Stage IV) melanoma | _ |
| **IL-2 + CAR-T cell therapy** | CD19 targeted CAR-T cells | NCT00924326, Phase I/II trial with CD19 targeted CAR-T cells for B cell lymphoma, Completed (Nov 2021) | Complete remissions of a variety of B-cell malignancies lasting ≥ 3 years, remissions of up to 9 years are ongoing, rare late adverse events [33] |
|  | IL-13Rα2 CAR-T Cells | NCT04119024, Phase I trial for the treatment of stage IIIC or IV melanoma, Recruiting (Nov 2019) | _ |
| ***Orthogonal IL-2/IL-2Rβ mutant pairs*** | | | |
| **Orthogonal IL-2/IL-2Rβ mutant pairs** | CD19-specific CAR-T cells in combination with ortho-hIL-2Rβ | Immunodeficient mice bearing CD19+ Nalm6 leukemia xenografts | Antileukemic effect [34] |
| **SYNCAR-001/STK-009** | CD19 specific CAR-T cells (SYNCAR-001) in combination with ortho-hIL-2Rβ (STK-009) | Preclinical models of human CAR-refractory lymphoma | Systemic and intra-tumoral expansion and activation of SYNCAR-001, Complete responses in large subcutaneous lymphomas [35] |
|  |  | Phase I trial in subjects with relapsed or refractory CD19 expressing hematologic malignancies, Recruiting (Feb 2023) | _ |
| **SYNCAR-002/STK-009** | GPC3 specific CAR-T cells (SYNCAR-001) in combination with ortho-hIL-2Rβ (STK-009) | Highly aggressive s.c. and i.p. hepatocellular carcinoma models | Significant expansion of SYNCAR-002 within the peripheral blood, facilitating the infiltration of SYNCAR-002 cells into tumor sites, intra-tumoral production of granzyme B and IFN-γ by SYNCAR-002 [36] |

(i.v. – intravenous; i.p. – intraperitoneal; s.c. – subcutaneous)

**Bibliography (for Table S1)**

1. Johannsen M, Spitaleri G, Curigliano G, Roigas J, Weikert S, Kempkensteffen C, et al. The tumour-targeting human L19-IL2 immunocytokine: Preclinical safety studies, phase I clinical trial in patients with solid tumours and expansion into patients with advanced renal cell carcinoma. European Journal of Cancer. 2010;46(16):2926-35. doi: 10.1016/j.ejca.2010.07.033
2. Weide B, Eigentler TK, Pflugfelder A, Zelba H, Martens A, Pawelec G, et al. Intralesional Treatment of Stage III Metastatic Melanoma Patients with L19–IL2 Results in Sustained Clinical and Systemic Immunologic Responses. Cancer Immunology Research. 2014;2(7):668-78. doi: 10.1158/2326-6066.cir-13-0206
3. Danielli R, Patuzzo R, Di Giacomo AM, Gallino G, Maurichi A, Di Florio A, et al. Intralesional administration of L19-IL2/L19-TNF in stage III or stage IVM1a melanoma patients: results of a phase II study. Cancer Immunology, Immunotherapy. 2015;64(8):999-1009. doi: 10.1007/s00262-015-1704-6
4. Nidlegy™ Phase III PIVOTAL trial meets the study’s primary objective demonstrating statistically significant and clinically meaningful improvement in Recurrence-Free Survival for patients with locally advanced fully resectable melanoma. OCT 16, 2023. Available from: <https://www.nasdaq.com/press-release/nidlegytm-phase-iii-pivotal-trial-meets-the-studys-primary-objective-demonstrating>
5. Albertini MR, Hank JA, Gadbaw B, Kostlevy J, Haldeman J, Schalch H, et al. Phase II trial of hu14.18-IL2 for patients with metastatic melanoma. Cancer Immunol Immunother. 2012;61(12):2261-71. doi: 10.1007/s00262-012-1286-5
6. Moser JC, Opyrchal M, Rodriguez-Rivera II, Bilen MA, Curti B, Sosman JA, Puzanov I, Pili R, Morris K, Nirschl CJ, Park S, Bruno M, Windt P, Subramanian KK, Chopra SS, Isaacs R. A Phase 1/1b Study of the Tumor-Activated IL-2 Prodrug WTX-124 Alone or in Combination with Pembrolizumab in Patients with Immunotherapy-Sensitive Locally Advanced or Metastatic Solid Tumors. Abstract 737. SITC 2023.
7. Santos J, Peltola K, Alanko T, et al1518 T-cell inducing oncolytic virus (igrelimogene litadenorepvec; TILT-123) shows safety, anti-tumor activity and induction of immune responses in advanced solid tumor patients (full report on TUNIMO)Journal for ImmunoTherapy of Cancer 2023;11:doi: 10.1136/jitc-2023-SITC2023.1518.
8. Pakola SA, Peltola KJ, Clubb JH, Jirovec E, Haybout L, Kudling TV, Alanko T, Korpisaari R, Juteau S, Jaakkola M, Sormunen J. Safety, Efficacy, and Biological Data of T-Cell–Enabling Oncolytic Adenovirus TILT-123 in Advanced Solid Cancers from the TUNIMO Monotherapy Phase I Trial. Clinical Cancer Research. 2024 Apr 25:OF1-1.
9. Merchant R, Galligan C, Munegowda MA, Pearce LB, Lloyd P, Smith P, et al. Fine-tuned long-acting interleukin-2 superkine potentiates durable immune responses in mice and non-human primate. Journal for Immunotherapy of Cancer. 2022;10(1):e003155. doi: 10.1136/jitc-2021-003155
10. Sulzmaier FJ, Kern N, Ahn SJ, et alINBRX-120, a CD8α-targeted detuned IL-2 that selectively expands and activates tumoricidal effector cells for safe and durable in vivo responsesJournal for ImmunoTherapy of Cancer 2023;11:e006116. doi: 10.1136/jitc-2022-006116
11. Vaishampayan UN, Tomczak P, Muzaffar J, Winer IS, Rosen SD, Hoimes CJ, et al. Nemvaleukin alfa monotherapy and in combination with pembrolizumab in patients (pts) with advanced solid tumors: ARTISTRY-1. Journal of Clinical Oncology. 2022;40(16_suppl):2500-. doi: 10.1200/jco.2022.40.16_suppl.2500
12. Atkinson VG, Antras JF, Bedard P, Brenner W, Brown J, Lemech CR, Lloyd P, Margolin K, Mathew M, Park JJ, Thomas S, Twardowski P, Gardner H, Prawira A, Coello M, Ebrahimimizadeh W, To MD, Merchant R, Karanam SM, Yavari A, Siu LL, Tawbi HA, Ascierto PA. Results from monotherapy dose escalation of MDNA11, a long-acting IL-2 superkine, in a phase 1/2 trial show evidence of single-agent activity in advanced solid tumors [abstract]. In: Proceedings of the American Association for Cancer Research Annual Meeting 2024; Part 2 (Late-Breaking, Clinical Trial, and Invited Abstracts); 2024 Apr 5-10; San Diego, CA. Philadelphia (PA): AACR; Cancer Res 2024;84(7_Suppl):Abstract nr CT259.
13. Bentebibel S-E, Hurwitz ME, Bernatchez C, Haymaker C, Hudgens CW, Kluger HM, et al. A First-in-Human Study and Biomarker Analysis of NKTR-214, a Novel IL2Rβγ-Biased Cytokine, in Patients with Advanced or Metastatic Solid Tumors. Cancer Discovery. 2019;9(6):711-21. doi: 10.1158/2159-8290.cd-18-1495
14. Diab A, Tykodi SS, Daniels GA, Maio M, Curti BD, Lewis KD, et al. Bempegaldesleukin Plus Nivolumab in First-Line Metastatic Melanoma. J Clin Oncol. 2021;39(26):2914-25. doi: 10.1200/JCO.21.00675
15. Diab A, Hurwitz ME, Cho DC, Papadimitrakopoulou V, Curti BD, Tykodi SS, et al. NKTR-214 (CD122-biased agonist) plus nivolumab in patients with advanced solid tumors: Preliminary phase 1/2 results of PIVOT. Journal of Clinical Oncology. 2018;36(15_suppl):3006-. doi: 10.1200/jco.2018.36.15_suppl.3006
16. Tolaney S, Baldini C, Spira A, Cho D, Grignani G, Sawka D, et al. Clinical activity of BEMPEG plus NIVO observed in metastatic TNBC: preliminary results from the TNBC cohort of the Ph1/2 PIVOT-02 study. Poster A001 presented at the Fifth CRI-CIMT-EATI-AACR International Cancer Immunotherapy Conference 2019, Sep. 23–26, Paris.
17. Siefker-Radtke AO, Fishman MN, Balar AV, Grignani G, Diab A, Gao J, et al. NKTR-214 + nivolumab in first-line advanced/metastatic urothelial carcinoma (mUC): Updated results from PIVOT-02. Journal of Clinical Oncology. 2019;37(7_suppl):388-. doi: 10.1200/jco.2019.37.7_suppl.388
18. Robert C, Long GV, Brady B, Dutriaux C, Maio M, Mortier L, Hassel JC, Rutkowski P, McNeil C, Kalinka-Warzocha E, Savage KJ, Hernberg MM, Lebbé C, Charles J, Mihalcioiu C, Chiarion-Sileni V, Mauch C, Cognetti F, Arance A, Schmidt H, Schadendorf D, Gogas H, Lundgren-Eriksson L, Horak C, Sharkey B, Waxman IM, Atkinson V, Ascierto PA. Nivolumab in previously untreated melanoma without BRAF mutation. N Engl J Med. 2015 Jan 22;372(4):320-30. doi: 10.1056/NEJMoa1412082. Epub 2014 Nov 16.
19. Larkin J, Chiarion-Sileni V, Gonzalez R, Grob JJ, Cowey CL, Lao CD, Schadendorf D, Dummer R, Smylie M, Rutkowski P, Ferrucci PF, Hill A, Wagstaff J, Carlino MS, Haanen JB, Maio M, Marquez-Rodas I, McArthur GA, Ascierto PA, Long GV, Callahan MK, Postow MA, Grossmann K, Sznol M, Dreno B, Bastholt L, Yang A, Rollin LM, Horak C, Hodi FS, Wolchok JD. Combined nivolumab and ipilimumab or monotherapy in untreated melanoma. N Engl J Med. 2015 Jul 2;373(1):23-34. doi: 10.1056/NEJMoa1504030. Epub 2015 May 31. Erratum in: N Engl J Med. 2018 Nov 29;379(22):2185.
20. Tannir N, Formiga MN, Agarwal N, Pal SK, Cho D, George DJ, et al. LBA68 Bempegaldesleukin (BEMPEG) plus nivolumab (NIVO) compared to the investigator’s choice of sunitinib or cabozantinib in previously untreated advanced renal cell carcinoma (RCC): Results from a phase III randomized study (PIVOT-09). Annals of Oncology. 2022;33:S1433. doi: 10.1016/j.annonc.2022.08.073
21. Falchook GS, Fu S, Lemech C, McKean MA, Azad A, Gan H, Sommerhalder D, Wang JS, Tan TJY, Chee CE, Barve M, Moser J, Mooney J, Acuff N, Wang R, Marina N, Abbadessa G, Streit M, Ramusovic S, Meniawy T. Phase I study of SAR444245 (SAR’245) as monotherapy (mono) and combined with pembrolizumab (pembro) or cetuximab (cetux) in patients (pts) with advanced solid tumors. Abstract| Volume 33, Supplement 7, S885-S886, September 2022. doi: 10.1016/j.annonc.2022.07.873
22. Janku F, Abdul-Karim R, Azad A, Bendell J, Falchook G, Gan HK, et al. Abstract LB041: THOR-707 (SAR444245), a novel not-alpha IL-2 as monotherapy and in combination with pembrolizumab in advanced/metastatic solid tumors: Interim results from HAMMER, an open-label, multicenter phase 1/2 Study. Cancer Research. 2021;81(13_Supplement):LB041-LB. doi: 10.1158/1538-7445.am2021-lb041
23. O'Neil J, Guzman W, Yerov O, Johnson P, Fanny M, Greene J, et al. Tumor-selective activity of XTX202, a protein-engineered IL-2, in mice without peripheral toxicities in nonhuman primates. Journal of Clinical Oncology. 2021;39(15_suppl):2563-. doi: 10.1200/jco.2021.39.15_suppl.2563
24. Walkey CD, Hara P, Tatalick L, Ulge U, Drachman J, Silva D-A. Abstract 4518: Pre-clinical development of NL-201: A *de novo* α-independent IL-2/IL-15 agonist. Cancer Research. 2020;80(16_Supplement):4518. doi: 10.1158/1538-7445.am2020-4518
25. Klein C, Waldhauer I, Nicolini VG, Freimoser-Grundschober A, Nayak T, Vugts DJ, et al. Cergutuzumab amunaleukin (CEA-IL2v), a CEA-targeted IL-2 variant-based immunocytokine for combination cancer immunotherapy: Overcoming limitations of aldesleukin and conventional IL-2-based immunocytokines. Oncoimmunology. 2017;6(3):e1277306-e. doi: 10.1080/2162402X.2016.1277306
26. Waldhauer I, Gonzalez-Nicolini V, Freimoser-Grundschober A, Nayak TK, Fahrni L, Hosse RJ, et al. Simlukafusp alfa (FAP-IL2v) immunocytokine is a versatile combination partner for cancer immunotherapy. MAbs. 2021;13(1):1913791-. doi: 10.1080/19420862.2021.1913791
27. Shanafelt AB, Lin Y, Shanafelt MC, Forte CP, Dubois-Stringfellow N, Carter C, et al. A T-cell-selective interleukin 2 mutein exhibits potent antitumor activity and is well tolerated in vivo. Nature Biotechnology. 2000;18(11):1197-202. doi: 10.1038/81199
28. Margolin K, Atkins MB, Dutcher JP, Ernstoff MS, Smith JW 2nd, Clark JI, Baar J, Sosman J, Weber J, Lathia C, Brunetti J, Cihon F, Schwartz B. Phase I trial of BAY 50-4798, an interleukin-2-specific agonist in advanced melanoma and renal cancer. Clin Cancer Res. 2007 Jun 1;13(11):3312-9. doi: 10.1158/1078-0432.CCR-06-1341
29. Gillessen S, Gnad-Vogt US, Gallerani E, Beck J, Sessa C, Omlin A, et al. A phase I dose-escalation study of the immunocytokine EMD 521873 (Selectikine) in patients with advanced solid tumours. European Journal of Cancer. 2013;49(1):35-44. doi: 10.1016/j.ejca.2012.07.015
30. Kosmac M, Malcolm TI, Shelton J, Craig HL, Zahn D, Wicher KB, Gillies SD, Van Krinks CH, McCourt MM. Kymab Ltd., Cambridgeshire, United Kingdom, Provenance Biopharmaceuticals Corp., Waltham, MA. / 10 - KY1043, a novel CD25-directed PD-L1 IL-2 immunocytokine, delivers potent anti-tumor activity in vivo via an expansion of a Tcf1hi PD-1+ CD8+ T cell population. In: Proceedings of the 111th Annual Meeting of the American Association for Cancer Research; 2020 June 22-24. Philadelphia (PA): AACR; 2020
31. Izar B, Zamarin D, Spigel DR, Hoimes CJ, McDermott DF, Sehgal K, Najjar YG, Schoenfeld AJ, Garon EB, Sullivan RJ, Henick BS, Leal TA, Hurwitz ME, McKay RR, Busby N, Mehta-Damani A, Azrilevich A, Tran T, Rizvi N, Oft M, Spira AI. Initial results from a phase 1a/1b study of STK-012, a first-in-class α/β IL-2 receptor biased partial agonist in advanced solid tumors (NCT05098132) [abstract]. In: Proceedings of the American Association for Cancer Research Annual Meeting 2024; Part 2 (Late-Breaking, Clinical Trial, and Invited Abstracts); 2024 Apr 5-10; San Diego, CA. Philadelphia (PA): AACR; Cancer Res 2024;84(7_Suppl):Abstract nr CT183.
32. Chesney J, Lewis KD, Kluger H, Hamid O, Whitman E, Thomas S, et al. Efficacy and safety of lifileucel, a one-time autologous tumor-infiltrating lymphocyte (TIL) cell therapy, in patients with advanced melanoma after progression on immune checkpoint inhibitors and targeted therapies: pooled analysis of consecutive cohorts of the C-144-01 study. Journal for immunotherapy of cancer. 2022;10(12):e005755. doi: 10.1136/jitc-2022-005755
33. Haslauer T, Greil R, Zaborsky N, Geisberger R. CAR T-Cell Therapy in Hematological Malignancies. Int J Mol Sci. 2021;22(16):8996. doi: 10.3390/ijms22168996
34. Zhang Q, Hresko ME, Picton LK, Su L, Hollander MJ, Nunez-Cruz S, et al. A human orthogonal IL-2 and IL-2Rβ system enhances CAR T cell expansion and antitumor activity in a murine model of leukemia. Science translational medicine. 2021;13(625):eabg6986-eabg. doi: 10.1126/scitranslmed.abg6986
35. Aspuria PJ, Vivona S, Bauer M, Semana M, Ratti N, McCauley S, et al. An orthogonal IL-2 and IL-2Rβ system drives persistence and activation of CAR T cells and clearance of bulky lymphoma. Science Translational Medicine. 2021;13(625). doi: 10.1126/scitranslmed.abg7565
36. Aspuria PJ, Semana M, Cheng I, Ratti N, Ramadass M, Rokkam D, Bauer M, Burgess R, Rosas H, Vivona S, Zheng G, Lupardus P, Oft M. Engineered human IL-2/IL-2Rß orthogonal pairs selectively enhance anti-GPC3 CAR T cells in vivo to drive complete responses in solid epithelial tumor models. Journal for ImmunoTherapy of Cancer. 2023;11:260-260. doi: 10.1136/jitc-2023-SITC2023.0228.
