# Supplementary material for: IL-2 based cancer immunotherapies: an evolving paradigm
Source: Front Immunol. 2024 Jul 24;15:1433989. doi: 10.3389/fimmu.2024.1433989 (PMC11303236; doi:10.3389/fimmu.2024.1433989)
Supplement: Supplementary file 3 [file Table_2.docx]

**Table S2: List of IL-2 based Clinical Trials for Cancer Registered on ClinicalTrials.gov**

| **Sr No** | **Trial Identifier** | **Drug** | **Combo** | **Sponsor/Owner** | **Condition** | **Phase** | **RoA** | **Recruitment Status/ Results** |
| --- | --- | --- | --- | --- | --- | --- | --- | --- |
| 1 | NCT05821686 | Proleukin |  | Gregory Knapp, Nova Scotia Health Authority | Triple Negative Breast Cancer | I, II | intralesional, peri-tumoral IL-2 | Not yet recruiting |
| 2 | NCT02964078 | Proleukin | Pembro | H. Lee Moffitt Cancer Center and Research Institute; Merck Sharp & Dohme LLC; Prometheus Laboratories | Metastatic Kidney Cancer | II | i.v. | ORR of 69%, with ORR 90%-lower confidence bound of 55%. Study showed feasibility of a combination of HD IL-2 and pembrolizumab, with a high response rate.^1^ |
| 3 | NCT05493566 | Proleukin | Pembro | Emory University, National Cancer Institute (NCI) | NSCLC | I | low dose | Recruiting |
| 4 | NCT01883297 | Proleukin | Restimulated TILs, Cyclophosphamide | University Health Network, Toronto | Recurrent, Platinum Resistant  High Grade Serous Ovarian, Fallopian Tube, or Primary Peritoneal Cancer | I | s.c. low dose IL-2 | Active, not recruiting |
| 5 | NCT05475847 | IL-2 | Autologous TIL (C-TIL052A); lymphodepletion | Fudan University; Cellular Biomedicine Group Ltd. | Advanced Cervical Cancer | I |  | Recruiting |
| 6 | NCT05307874 | IL-2 | ICT01 - anti-BTN3A mAb | ImCheck Therapeutics; ILife Consulting; Exystat | Advanced Solid Tumors | I, II | s.c. low dose IL-2 | Expansion of γ9δ2 T cells in 11/11 evaluable patients with no dose limiting toxicities; broad immune activation, mobilization and proliferation of CD8+ T cells, NKs and granulocytes; increase in PD-1 and PD-L1 expression on various immune cell populations.^2^ |
| 7 | NCT05141474 | IL-2 | NEXTGEN-TIL; Non-myeloablative Lymphodepletion | Vall d'Hebron Institute of Oncology; Banc de Sang i Teixit | Advanced Epithelial Tumors | I | i.v. | Recruiting |
| 8 | NCT04862767 | IL-2 | TASO-001 - TGF-β2 targeting anti-sense oligonucleotide | Autotelicbio | Advanced or Metastatic Solid Tumor | I | s.c. | Recruiting |
| 9 | NCT04892498 | IL-2 | Hypofractionated Radiotherapy; PD-1 Inhibitor; GM-CSF | Second Affiliated Hospital of Soochow University | Advanced Refractory Solid Tumors | II |  | Median PFS of 5.8 months, median OS of 13.5 months; ORR of 21.4 %, and the disease control rate (DCR) of 61.9 %; Lower plasma levels of IL-6 and IL-17 at baseline to be associated with improved PFS. TRAE experienced 78.6% patients, with 9.5% patients experiencing Grade ≥ 3 TRAEs^3^. |
| 10 | NCT05115500 | IL-2 | RC48-ADC (Her2); Hypofractionated Radiotherapy; PD-1 Inhibitor; GM-CSF | Second Affiliated Hospital of Soochow University | HER2-expressing Advanced Refractory Solid Tumors | II |  | ORR of 38.5%, and DCR of 69.2%; ORR of 66.7% in gynecological cancer, 25.0% in pancreatic cancer, and 31.3% in other cancers; Median PFS of 7.2 months; Occurrence of grade ≥3 TRAEs in two patients (6.3%)^4^. |
| 11 | NCT05122221 | IL-2 | CRTE7A2-01 TCR-T Cell; Fludarabine + Cyclophosphamide | Corregene Biotechnology Co., Ltd | HPV-16 Positive Advanced Cervical, Anal,  or Head and Neck Cancers | I |  | Recruiting |
| 12 | NCT02306954 | IL-2 | Stereotactic Body Radiation (SBRT) | Providence Health & Services; Prometheus Laboratories;  Cytokine Working Group | Metastatic Renal Cancer | II | HD IL-2: 600,000 IU/kg by i.v. bolus (IVB) | Active, not recruiting |
| 13 | NCT02919644 | IL-2 | autologous dendritic cells loaded with autologous tumour homogenate | Istituto Scientifico Romagnolo per lo Studio e la cura dei Tumori | Stage IV Colorectal Cancer | II |  | Recruiting |
| 14 | NCT03991741 | IL-2 | Autologous Tumor Infiltrating Lymphocytes | Gregory Daniels; Immunotherapy Foundation; UCSD | Select Solid Tumors | I | HD IL-2 | Recruiting |
| 15 | NCT04643574 | IL-2 | NeoTIL; Cyclophosphamide; Fludarabine; Radiotherapy | Centre Hospitalier Universitaire Vaudois | Solid Tumor | I | HD IL-2 | Recruiting |
| 16 | NCT03891706 | IL-2 | tumor-specific TCR-T cell (FIT-001) | Guangzhou FineImmune Biotechnology Co; Sun Yat-sen University | Solid Tumor | I | 3,000,000 IU, i.v. QD for 5 days | Recruiting |
| 17 | NCT04166006 | IL-2 | Autologous DC vaccine | Istituto Scientifico Romagnolo per lo Studio e la cura dei Tumori | Resected Stage IV Rare Cancers. | II | 3 MU, s.c., daily for 5 days | Recruiting |
| 18 | NCT05530200 | IL-2 | Hypofractionated Radiotherapy; PD-L1 Inhibitor; GM-CSF | Second Affiliated Hospital of Soochow University | Advanced Refractory Solid Tumors | II | 2 million IU, day 8 - day 14 | Not yet recruiting |
| 19 | NCT05676749 | IL-2 | C-TIL051; lymphodepletion; Pembro | Cellular Biomedicine Group, Inc. | Anti-PD1 Resistant Metastatic NSCLC | I |  | Not yet recruiting |
| 20 | NCT01319565 | IL-2 | TIL; Cycloph-osphamide; Fludarabi-ne; Radiotherapy | NCI; National Institutes of Health Clinical Center | Metastatic Melanoma | II | HD IL-2 | Durable complete regressions in 24% of patients with metastatic melanoma and a median survival greater than 3 years^5^. |
| 21 | NCT05400122 | IL-2 | non-HLA-matched donor NK cells; TGFbR1 Inhibitor Vactosertib;  Cyclophosphamide; Fludarabine | Jennifer Eva Selfridge; Case Comprehensive Cancer Center | colorectal cancer and blood cancers | I | 6 MU s.c., 3x/week or 2x/week for up to 4 weeks total | Recruiting |
| 22 | NCT05385705 | IL-2 | allogeneic NK cells; Cyclophosphamide; Trastuzumab; Pertuzumab | Vall d'Hebron Institute of Oncology | Refractory Metastatic Her2 Pos Breast Cancer | I | Day 2 (within 24h after NK infusion), 4 and 6; s.c.; dose of 5x105 UI/m2 | Recruiting |
| 23 | NCT05603013 | IL-2 | Vinorelbine; Radiotherapy; PD-1/L1 Inhibitor; GM-CSF | Second Affiliated Hospital of Soochow University | Non-Small-Cell Lung & breast cancer | II | s.c. | Not yet recruiting |
| 24 | NCT05798546 | IL-2 | Neoantigen Targeting T Cells (GI-NeoT-02)  ; Fludarabine + Cyclophosphamide | BGI, China; Fudan University | Solid Tumor | I | 500,000 IU/m^2 s.c., after each cell infusion, IL-2 will start within 24 hr and every 8-12 hr for up to 6 doses | Recruiting |
| 25 | NCT05798533 | IL-2 | Neoantigen Targeting T Cells (GI-NeoT-03)  ; Fludarabine + Cyclophosphamide | BGI, China; Fudan University; Shanghai 10th People's Hospital | Solid Tumor | I | 500,000 IU/m^2 s.c. after each cell infusion, IL-2 will start within 24 hr and every 8-12 hr for up to 6 doses | Recruiting |
| 26 | NCT04426669 | IL-2 | CISH KO TIL; Fludarabine + Cyclophosphamide | Intima Bioscience, Inc.; Masonic Cancer Center, University of Minnesota | Gastrointestinal Epithelial Cancer | I, II | Days 1-4: Aldesleukin at 720,000 U/kg as an i.v. infusion, every 8 -12 hr but, no more than 24 hr apart as tolerated for up to 6 doses. | Recruiting |
| 27 | NCT01697527 | IL-2 | NY-ESO-1 reactive TCR retroviral vector transduced autologous PBL; NY-ESO-1-157-165 peptide pulsed dendritic cell vaccine; Fludarabine + Cyclophosphamide | Jonsson Comprehensive Cancer Center | Malignant Neoplasm | II | s.c. BID on days 1-14. | Two out of four patients (50%) displayed evidence of tumor regression; rapid *in vivo* expansion of NY-ESO-1-specific T cells in peripheral blood; shifting of transgenic cells from memory phenotypes to more terminally differentiated effector phenotypes over time^6^. |
| 28 | NCT05194735 | IL-2 | Autologous Neoantigen specific TCR-T cell drug product | Alaunos Therapeutics | relapsed/refractory solid tumors | I, II | tumor mutation and HLA typing combination that matches to at least one of the following TCRs in the Alaunos' library | Recruiting |
| 29 | NCT05505812 | IL-2 | HS-IT101 - autologous TILs; fludarabine and cyclophosphamide | Affiliated Hospital of Qingdao University; Qingdao Sino-Cell Biomedicine Co | Breast Cancer | I |  | Not yet recruiting |
| 30 | NCT04729543 | IL-2 | MAGE-C2/HLA-A2 TCR T cells (MC2 TCR T cells); 5' Azacytidine (AZA) + Valproic acid (VP) | Erasmus Medical Center | Melanom & HNSCC | I, II |  | Recruiting |
| 31 | NCT03658785 | IL-2 | autologous TIL specific to personalized Neo-antigens; fludarabine and cyclophosphamide | Tongji Hospital | Solid Tumors | I, II | 720,000 IU/kg i.v.; approximately every 8 hr (+/- 1hr) beginning  within 24 hr of cell infusion and continuing for up to 5 days (maximum of 15 doses.) | Not yet recruiting |
| 32 | NCT03240861 | IL-2 | NY-ESO-1 TCR Engineered (PBMC) and Peripheral Blood Stem Cells (PBSC); busulfan & fludarabine | Jonsson Comprehensive Cancer Center | NY-ESO-1 Positive advanced cancer | I | aldesleukin s.c. BID for up to 7 days | Recruiting |
| 33 | NCT05681780 | IL-2 | TIL stimulated with CD40; Nivo; fludarabine and cyclophosphamide | H. Lee Moffitt Cancer Center and Research Institute | EGFR, ALK, ROS1 or HER2-Driven NSCLC | I, II |  | Recruiting |
| 34 | NCT03215810 | IL-2 | TIL; Nivo; fludarabine and cyclophosphamide | H. Lee Moffitt Cancer Center and Research Institute | Advanced Non-Small Cell Lung Cancer | I |  | 3 of 13 patients had confirmed responses and 11 had reduction in tumor burden, with a median best change of 35%; complete responses in 2 patients that were ongoing 1.5 years later; enrichment of T cells recognizing multiple types of cancer mutations after TIL treatment in responding patients; increase of neoantigen-reactive T cell clonotypes and persistence in peripheral blood after treatment^7^. |
| 35 | NCT05336409 | IL-2 | CNTY-101 - allogeneic iPSC-derived anti-CD19-CAR/IL-15-expressing NK cells;  Lymphodepleting Chemotherapy | Century Therapeutics | CD19-Positive B-Cell Malignancies | I | IL-2 s.c. | Recruiting |
| 36 | NCT05802056 | IL-2 | nivolumab and standard chemotherapy | Mayo Clinic | Gastric Cancer with Peritoneal Metastasis | I | IL-2 i.p. | Not yet recruiting |
| 37 | NCT05831033 | IL-2 | BEN101 - Autologous TIL | Shanghai General Hospital, Shanghai Jiao Tong University School of Medicine | Advanced Solid Tumors | I | Administer 8-16 hr after TIL infusion. 600,000 IU/kg i.v. over 15-20 min every 12 hr. It is recommended to start with high dose; and de-escalate based on tolerability, up to 5 days | Not yet recruiting |
| 38 | NCT05539768 | IL-2 | HS-IT101 - autologous TILs; fludarabine and cyclophosphamide | The Affiliated Hospital of Qingdao University | Advanced Solid Tumors | I |  | Not yet recruiting |
| 39 | NCT01038778 | IL-2 | entinostat (HDAC inhibitor) | National Cancer Institute (NCI) | Metastatic Kidney Cancer | I, II | HD aldesleukin i.v. every 8 hr on days 1-5 and 15-19 | 39% of OR with 9 PR and 3 CR; 81 days of median time to response; median PFS not reached with a median follow-up of 437 days; decreased Tregs and monocytic MDSCs following treatment; an association of increased CD14+/CD86+ and CD14+HLADR+ cells with objective responses^8^. |
| 40 | NCT04165967 | IL-2 | Autologous TIL, Nivo; cyclophosphamide and fludarabine | University Hospital, Basel, Switzerland | Advanced Melanoma | I | low dose IL-2: 125.000 IU/kg/day s.c. for maximum 12 days as inpatients with a 2 days break after the first 4-5 doses | Active, not recruiting |
| 41 | NCT03260504 | IL-2 | Pembro | University of Washington | Advanced or Metastatic Kidney Cancer | I | aldesleukin s.c.5 days per week for 6 weeks; or aldesleukin i.v. on days 2-6 of pembrolizumab cycles 1 and 2 | Recruiting |
| 42 | NCT05152797 | IL-2 | Autologous TIL; non-myeloablative (NMA) lymphodepletion | Women's Hospital School Of Medicine Zhejiang University | Advanced or Metastatic Refractory Gynecological Cancer | I, II |  | Not yet recruiting |
| 43 | NCT02621021 | IL-2 | young tumor infiltrating lymphocytes (Young TIL); pedmbro;  cyclophosphamide and fludarabine | National Cancer Institute (NCI) | Melanoma | II | 720,000 IU/kg i.v. (based on total body weight) over 15 min approximately every 8 hr (+/- 1 hr) beginning within 24 hr of cell infusion and  continuing for up to 4 days (maximum 12 doses). | ORR of 56% (108/192) and median melanoma-specific survival of 28.5 months in patients naïve to anti–programmed cell death-1 (PD-1) therapy compared with 24% (8/34) and 11.6 months in patients refractory to anti–PD-1 (aPD-1); prior treatment with targeted molecular therapy associated with a decreased response rate (21% vs. 60%) and decreased survival (9.3 vs. 50.7 months) in patients with BRAF V600E/K–mutated disease, than patients naïve to targeted therapy; with a median follow-up of 89 months, 46 of 48 complete responders in the aPD-1–naïve cohort have ongoing responses after a single treatment and 10-year melanoma-specific survival of 96%^9^. |
| 44 | NCT04629729 | IL-2 | FT819 - an off-the-shelf CAR T-cell; Bendamustine, cyclophosphamide and fludarabine | Fate Therapeutics | B-cell Malignancies | I |  | Recruiting |
| 45 | NCT04111510 | IL-2 | autologous TIL LN-145; nonmyeloablative lymphodepletion | Yale University; Iovance Biotherapeutics | Metastatic Triple Negative Breast Cancer | II | i.v.; up to six doses maximum | Active, not recruiting |
| 46 | NCT03691376 | IL-2 | Autologous NY-ESO-1-specific CD8-positive T Lymphocytes (PBMC, PBSC) Melphalan | Roswell Park Cancer Institute | Recurrent or Refractory Ovarian, Fallopian Tube, or Primary Peritoneal Cancer | I |  | Active, not recruiting |
| 47 | NCT04812470 | IL-2 | Autologous Tumor Infiltrating Lymphocytes; Melphalan | Vastra Gotaland Region | Melanoma and Liver Metastases | I | IL-2 will be administered s.c. once daily for up to 14 days | Recruiting |
| 48 | NCT05296564 | IL-2 | Anti-NY-ESO-1 TCR-Gene Engineered Lymphocyte (HBI 0201-ESO TCRT); cyclophosphamide and fludarabine | Hadassah Medical Organization | NY-ESO-1 -Expressing Metastatic Cancers | I, II | Continuous infusion of aldesleukin 18x10E6 IU/24 hr will be given 24 hr post HBI 0201-ESO TCRT infusion, for four days or until a dose limiting toxicity | Recruiting |
| 49 | NCT05724732 | IL-2 | Autologous TIL (GT201); cyclophosphamide + fludarabine | RenJi Hospital | Advanced Gynecologic Tumors | I |  | Recruiting |
| 50 | NCT03610490 | IL-2 | Autologous TIL MDA-TIL; cyclophosphamide + fludarabine | M.D. Anderson Cancer Center | Recurrent or Refractory Ovarian Cancer, Colorectal Cancer, or Pancreatic Ductal Adenocarcinoma | II | IL-2 i.v. over 30 min on days 1-4 for up to 6 doses | Prolonged SD in a patient with PDAC lasting until 18 months; Grade 3 or higher toxicities were seen in 14 of 17 subjects; presence of effector memory cells with high expression of CD39^10^. |
| 51 | NCT05451784 | IL-2 | PD1+ TILs (NUMARZU-001); NMA-LD chemotherapy | Fundacio Clinic Barcelona | Metastatic Triple-Negative Breast Carcinoma | I, II |  | Not yet recruiting |
| 52 | NCT04674488 | IL-2 | autologous TIL; non-myeloablative (NMA) lymphodepletion | Shanghai OriginCell Therapeutics Co., Ltd. | Metastatic or Recurrent Cervical Cancer | I |  | Recruiting |
| 53 | NCT05430360 | IL-2 | Autologous TIL (GT201); cyclophosphamide + fludarabine | Grit Biotechnology | Solid Tumor | I |  | Administration of GT201 associated with robust T cell expansion and prolonged persistence of infused TILs; good tolerability profile and anti-tumor activity^11^. |
| 54 | NCT05729399 | IL-2 | Autologous TIL (GT201); cyclophosphamide + fludarabine | Grit Biotechnology | Solid Tumor | I, II |  | Recruiting |
| 55 | NCT05430373 | IL-2 | autologous TIL GT101; cyclophosphamide + fludarabine | Grit Biotechnology | Solid Tumors | I |  | Recruiting |
| 56 | NCT05438667 | IL-2 | autologus TCR-T Cell Therapy; cyclophosphamide + fludarabine | Sun Yat-Sen Memorial Hospital of Sun Yat-Sen University | Pancreatic Cancer | I | IL-2 (injection unit: 500000 units /m², once every 12 hr, s.c. injection) will be injected i.v. for 14 days (24 times in total). | Recruiting |
| 57 | NCT04614103 | IL-2 | Autologous TIL LN-145; lymphodepleting chemotherapy | Iovance Biotherapeutics | Metastatic Non Small Cell Lung Cancer | II |  | Recruiting |
| 58 | NCT05497739 | IL-2 | intraperitoneal chemotherapy followed by adjuvant systemic chemotherapy | Cancer Institute and Hospital, Chinese Academy of Medical Sciences | Gastric Cancer | II | 2 million units/time on Days 4, 11, 18, and 25. | Recruiting |
| 59 | NCT05649618 | IL-2 | Autologous TIL; cyclophosphamide + fludarabine | Fujian Cancer Hospital | Solid Tumor | I | HD bolus IL-2 | Not yet recruiting |
| 60 | NCT02360579 | IL-2 | Lifileucel (LN-144) - Autologous TIL; non-myeloablative lymphodepletion | Iovance Biotherapeutics | Metastatic Melanoma | II |  | 36% ORR, 2 CR, 22 PR, DCR of 80% among 66 recruited patients; median duration of response did not reach after 18.7-month median study follow-up; consistent safety profile to that associated with lymphodepletion and IL-2^12^. |
| 61 | NCT00101101 | IL-2 | Autologous Tumor Cell-Based Vaccine and GM.CD40L; chemotherapy | H. Lee Moffitt Cancer Center and Research Institute | Lymphoma | II | low-dose IL-2 s.c. twice daily on days 1-14 | With a median follow-up of 67 months, the median overall survival (OS) has not yet been reached^13^. |
| 62 | NCT05605197 | IL-2 | U87 CAR-T; cyclophosphamide | Shanghai Unicar-Therapy Bio-medicine Technology Co | Pancreatic Cancer | I | i.v. IL-2 | Recruiting |
| 63 | NCT05361174 | IL-2 | IOV-4001 - PD-1 KO autologous TIL; lymphodepleting chemotherapy | Iovance Biotherapeutics | Melanoma, NSCLC | I, II |  | Recruiting |
| 64 | NCT03247309 | IL-2 | TCR-engineered T Cells - ACTengine IMA201-101; Fludarabine and Cyclophosphamide | Immatics US | Solid Tumors | I | low-dose IL-2 | Anti-tumor activity in 9 out of 10 evaluable patients showing disease control; tumor shrinkage in 8 out of 10 patients, one PR; robust engraftment, persistence and tumor infiltration of infused ACTengine® T cells; manageable safety and tolerability profile^14^. |
| 65 | NCT05438797 | IL-2 | Adoptive TIL-TCM transfer therapy; Abraxane + Cyclophosphamide | Sizhen Wang | Advanced Pancreatic Cancer | I | After cell infusion, IL-2 was administered at 720000 IU/kg (based on whole body weight) by i.v. every 8 hr for up to 4 days | Recruiting |
| 66 | NCT03108495 | IL-2 | Autologous TIL LN-145; Pembro; lymphodepleting chemotherapy | Iovance Biotherapeutics | Cervical Carcinoma | II |  | ORR of 44% (1 CR, 9 PR, 2 uPR); DCR of 89% at 3.5-month median study follow-up with 11/12 patients; acceptable safety and efficacy profile^15^. |
| 67 | NCT03474497 | IL-2 | Pembro; Radiotherapy | Megan Daly, MD | Refractory to Checkpoint Blockade | I, II | intralesional injection biweekly | Recruiting |
| 68 | NCT04562129 | IL-2 | Ipilimumab; Nivo | H. Lee Moffitt Cancer Center and Research Institute | Melanoma Stage III & IV | II | HD IL2 (600,000 units/kg/dose i.v.) will be given during week 1 of the 2 initial cycles or each course. | Recruiting |
| 69 | NCT01590069 | IL-2 |  | M.D. Anderson Cancer Center | Patients With Lung Metastases | I | Aerosolized Aldesleukin QD on days 1-21. Courses repeat every 28 days | Active, not recruiting |
| 70 | NCT02278887 | IL-2 | TIL; Cyclophosphamide + Fludarabine | The Netherlands Cancer Institute | Metastatic Melanoma | III | HD bolus IL-2 | Patients with advanced melanoma who received TIL therapy had significantly longer PFS than those who received ipilimumab^16^. |
| 71 | NCT05648994 | IL-2 | NY-ESO-1 TCR T cells; lymphodepleting chemotherapy | Fujian Cancer Hospital | Solid Tumors | I | HD IL-2 | Not yet recruiting |
| 72 | NCT05628883 | IL-2 | TBio-4101 autologous TIL; Cyclophosphamide + Fludarabine | H. Lee Moffitt Cancer Center and Research Institute | Relapsed/Refractory Melanoma | I | 600 000 IU/kg i.v. every 8 to 12 hr beginning within 24 hr after T-cell infusion | Recruiting |
| 73 | NCT05035407 | IL-2 | KK-LC-1 TCR (Kita-kyushu Lung Cancer Antigen);  Cyclophosphamide + Fludarabine | National Cancer Institute (NCI) | KK-LC-1 Positive Epithelial Cancers | I | dose of 720,000 IU/kg (based on total body weight) as an i.v. bolus over a 15 min period beginning within 24 hr of cell infusion and continuing for up to four days | Recruiting |
| 74 | NCT03475134 | IL-2 | TIL; Nivo; Cyclophosphamide + Fludarabine | Centre Hospitalier Universitaire Vaudois | Metastatic Melanoma | I | bolus administration every 8 hr, for a maximum of eight doses | ORR and DCR of 41.7% and 50% at 3 months; Two patients have an ongoing near-complete response at 3 years; median PFS of 4.8 months^17^. |
| 75 | NCT01701674 | IL-2 | ipilimumab followed by lymphodepletion + chemotherapy, autologous TIL | H. Lee Moffitt Cancer Center and Research Institute | Metastatic Melanoma | Pilot Study | HD IL-2 about 3-5 days as an i.v. bolus | Of the 13 enrolled patients, 6 responders (46%) at 12 weeks following infusion; Median PFS of 7.4 months^18^. |
| 76 | NCT03546426 | IL-2 | Autologous DC loaded with autologous tumor homogenate; Pembro | Istituto Scientifico Romagnolo per lo Studio e la cura dei Tumori | Mesothelioma | I | After each vaccine administration patients will receive IL-2 3 MU s.c. for 5 days, from day +2 to day +6 | Recruiting |
| 77 | NCT05435768 | IL-2 | RANKL inhibitor Denosumab; radiotherapy, aPD1, GMCSF; | Second Affiliated Hospital of Soochow University | Metastatic Solid Tumor | I, II | IL-2 (2 million IU/d) for 7 days | Not yet recruiting |
| 78 | NCT05395052 | IL-2 | FT536 - MICA/B-targeted allogeneic NK cell; Monoclonal Antibodies;  Cyclophosphamide + Fludarabine | Fate Therapeutics | Solid Tumors | I |  | Active, not recruiting |
| 79 | NCT02748564 | IL-2 | Pembro | Rutgers, The State University of New Jersey | Stage III-IV Melanoma | II | aldesleukin i.v. every 8 hr for up to 14 doses at weeks 4, 7, 16, 19, 28, and 31 | More frequent adverse events with increasing doses of IL-2; no dose limiting toxicities; the MTD of IL-2 was not reached; one partial response in 9 patients (11%)^19^. |
| 80 | NCT05566223 | IL-2 | CISH Inactivated TILs; Pembro; Cyclophosphamide + Fludarabine | Intima Bioscience | NSCLC | I, II | Days 1-4: Aldesleukin at 720,000 U/kg as an i.v. infusion, every 8 -12 hr but, no more than 24 hr apart as tolerated for up to 6 doses | Not yet recruiting |
| 81 | NCT04310592 | IL-2 | CYNK-001 - allogeneic NK cells derived from human placental CD34+ cells; Cyclophosphamide + Fludarabine | Celularity Incorporated | AML | I |  | Recruiting |
| 82 | NCT03017131 | IL-2 | NY-ESO-1-specific TCR T cells; Cyclophosphamide, Fludarabine, Decitabine | Roswell Park Cancer Institute | ovarian, primary peritoneal, fallopian tube cancer | I | low-dose IL-2 for 2 weeks from Day 1 to Day 14 | Active, not recruiting |
| 83 | NCT04551885 | IL-2 | FT516 - Off-the-Shelf iPSC-Derived NK Cell Therapy; Avelumab; Cyclophosphamide + Fludarabine | Fate Therapeutics | Solid Tumor | I |  | Active, not recruiting |
| 84 | NCT05366478 | IL-2 | LM103 - autologous TIL; cyclophosphamide + fludarabine | Suzhou BlueHorse Therapeutics Co | Solid Tumors | I |  | Recruiting |
| 85 | NCT05238818 | IL-2 | Autologous TIL expressing membrane-bound IL-12 (GT202); lymphodepletion | XinWu, Obstetrics & Gynecology Hospital of Fudan University | Gynecological Tumors | I |  | Recruiting |
| 86 | NCT04119024 | IL-2 | IL13Ralpha2 CAR T cells; cyclophosphamide + fludarabine | Jonsson Comprehensive Cancer Center | Stage IIIC or IV Melanoma | I | IL-2 s.c. BID on days 1-7 | Recruiting |
| 87 | NCT05576077 | IL-2 | TBio-4101 - autologous, neoantigen-selected TIL; Pembro;  cyclophosphamide + fludarabine | Turnstone Biologics | Solid Tumors | I |  | Recruiting |
| 88 | NCT05607095 | IL-2 | Lifileucel (LN-144) - Autologous TIL; non-myeloablative lymphodepletion | Memorial Sloan Kettering Cancer Center | Metastatic Uveal Melanoma | I |  | Recruiting |
| 89 | NCT01955460 | IL-2 | NGFR-transduced Autologous TILs, TGFbDNRII-transduced Autologous TILs; cyclophosphamide + fludarabine | M.D. Anderson Cancer Center | Stage IIIC or IV Melanoma | I | HD aldesleukin i.v. over 15 min every 8-16 hr on days 1-5 (up to 15 doses) and 22-26 (up to 15 doses) | Initial DCR of 86% in 7 heavily pretreated patients; 2 patients (29%) showed persistent response lasting longer than 6 months; 90% drop in tumor burden in 1 patient at 17 months; no increase in toxicity^20^. |
| 90 | NCT05640193 | IL-2 | Lifileucel (LN-144) - Autologous TIL; non-myeloablative lymphodepletion | Memorial Sloan Kettering Cancer Center | Asymptomatic Melanoma Brain Metastases | I | short course of HD IL-2 | Recruiting |
| 91 | NCT04347616 |  | allogeneic UCB-NK cells | Radboud University Medical Center | relapsed or refractory acute myeloid leukemia | I, II | IL-2 will be administered in a fixed dose of 3.0 x 10^6 or 6.0 x 10^6 units starting 4 hr after NK cell infusion and given every other day for 6 doses in total. | Recruiting |
| 92 | NCT03068624 | IL-2 | autologous CD8 positive (+) SLC45A2-specific T cells; Ipilimumab; cyclophosphamide | M.D. Anderson Cancer Center | Metastatic Uveal Melanoma | I | aldesleukin BID s.c. for 14 days | SD in 4 (36%) patients with the median duration of SD of 5.7 months, PD in 6 (55%); Median OS of 8.9 weeks; OS was 91% at 4 weeks, 55% at 8 weeks and 46% at 13 weeks; median PFS of 5.9 weeks^21^. |
| 93 | NCT05207722 | IL-2 | CYNK-101 - NK cells derived from human placental CD34+; Pembro; Trastuzumab; cyclophosphamide + fludarabine + Mesna | Celularity Incorporated | Metastatic HER2 Positive Gastroesophageal Junction Cancer | I, II | 6 million (M) IU of rhIL-2 administered s.c. on each CYNK-101 infusion day. | Active, not recruiting |
| 94 | NCT04052334 | IL-2 | TIL; cyclophosphamide + fludarabine | H. Lee Moffitt Cancer Center and Research Institute | Sarcoma | I | IL-2 600 000 IU/kg i.v. bolus (about 15 min) every 8 to 16 hr for up to 15 doses, beginning approximately 8 to 16 hr after T-cell infusion | Active, not recruiting |
| 95 | NCT03645928 | IL-2 | autologous TIL LN-144 (Lifileucel)/LN-145/LN-145-S1; Pembro; Nivo; Ipi; NMA lymphodepletion | Iovance Biotherapeutics | Solid Tumors | II |  | Recruiting |
| 96 | NCT01005745 | IL-2 | TIL; cyclophosphamide + fludarabine | H. Lee Moffitt Cancer Center and Research Institute | Metastatic Melanoma | Not Applicable | HD IL-2, 720,000 IU/kg i.v. bolus (about 15 min) every 8-16 hr for up to 15 doses, beginning approximately 12-16 hr after T cell infusion. | Evidence of CD4+ TIL involvement in complete clinical responses after ACT^22^. |
| 97 | NCT04024761 | IL-2 | cytokine induced memory-like natural killer (CIML NK); cyclophosphamide + fludarabine | Dana-Farber Cancer Institute | Myeloid Disease | I |  | Rapid expansion and long-term persistence of CIML NK cells^23^. |
| 98 | NCT03449108 | IL-2 | autologous TIL LN-145 or LN-145-S1; Nivo; Ipi cyclophosphamide + fludarabine | M.D. Anderson Cancer Center | solid tumor, sarcoma | II | aldesleukin i.v. over 30 min on days 1-4 for up to 6 doses | Recruiting |
| 99 | NCT05727904 | IL-2 | Lifileucel plus Pembrolizumab | Iovance Biotherapeutics | Advanced Melanoma | III |  | Recruiting |
| 100 | NCT04673617 | IL-2 | AB-101 - off-the shelf, allogeneic NK cell; Rituximab; cyclophosphamide + fludarabine | Artiva Biotherapeutics | Non Hodgkin Lymphoma | I, II |  | Recruiting |
| 101 | NCT04625205 | IL-2 | NEO-PTC-01 - autologous personalized T cell; αPD-1 | BioNTech US Inc | Melanoma | I |  | Recruiting |
| 102 | NCT04318964 | IL-2 | TAEST16001 cells - NY-ESO-1 TCR T cells; lymphodepletion | Sun Yat-sen University | Soft Tissue Sarcoma - NY-ESO-1 (HLA-A * 02:01) | I | IL-2 s.c. (study day 1 to day 14), 500000 U / time | Acceptable tolerability profile; of 12 patients, 5 showed PR, 5 had SD, and 2 had PD; ORR was 41.7%; median duration of response was 14.1 months^24^. |
| 103 | NCT05080790 | IL-2 | Dinutuximab Beta (binds GD2 antigen), Zoledronic Acid | Institut für Klinische Krebsforschung IKF GmbH | Leiomyosarcoma | II | IL-2, 5.4x10^6; Q5W | Recruiting |
| 104 | NCT03450122 | IL-2 | Autologous NY-ESO-1-specific CD8-positive T cells; Dendritic Cell-targeting Lentiviral Vector ID-LV305; cyclophosphamide | M.D. Anderson Cancer Center | NY-ESO-1 Positive Sarcoma | I | aldesleukin s.c., starting 6 hr after cell transfer and twice a day for 14 days, receive | Active, not recruiting |
| 105 | NCT04383067 | IL-2 | autologous TIL | Sheba Medical Cente | Metastatic Urothelial Carcinoma | II | HD (720,000 IU/kg) IL-2 administered every 8 hr, to tolerance. A maximum of 10 doses. | Recruitment status unknown |
| 106 | NCT01946373 | IL-2 | TIL; DC pulsed with autologous tumor lysate and NY-ESO-1 peptide; cyclophosphamide + fludarabine | Karolinska University Hospital | Melanoma | I | IL-2 90 min after T cell infusion at a dose of 100,000 IU/kg as i.v. bolus over 15 min period every 8-hr for up to 14 doses | Clinical responses reported by TIL therapy combined with DC vaccination in 4 out of 4 treated MM patients who previously failed ICI^25^. |
| 107 | NCT00338377 | IL-2 | TIL; DC vaccine; cyclophosphamide + fludarabine + Mesna | M.D. Anderson Cancer Center | Metastatic Melanoma | II | IL-2: 12-16 hr after T cell infusion at standard dose of 720,000 IU/kg as i.v. bolus over 15 min period every 8-16 hr for up to 15 doses on Days 1-5 and 22-26 | The ORR was 30% (3/10) in the TIL arm and 50% (4/8) in the TIL+DC arm; All treatments were well tolerated^26^. |
| 108 | NCT05357027 | IL-2 | HPV16 E6 TCR T Cells (TC-E202); cyclophosphamide + fludarabine | TCRCure Biopharma | Cervical Carcinoma | I, II | Following cell infusion, HD bolus IL-2, which is dosed to individual patient tolerance | Recruiting |
| 109 | NCT03991130 | IL-2 | Nivolumab | Gregory Daniels | Metastatic Melanoma and Renal Cell Carcinoma | II | standard HD IL-2 administered every 8 hr for up to 14 doses days 8-12 and day 22-28 | Tolerance and disease control observed among the three MM patients^27^. |
| 110 | NCT03501381 | IL-2 | Entinostat | Roberto Pili | RCC | II | up to 3 courses of HD IL-2 600,000 units/kg administered i.v. every 8 hr on Days 1-5 and Days 15-19 (maximum 28 doses) | Preliminary results from this study confirm that the combination of entinostat and HD-IL2 may be more active than HD-IL2 alone in patients with clear cell RCC^28^. |
| 111 | NCT01416831 | IL-2 | Radiation therapy | Providence Health & Services | Metastatic Melanoma | II | IL-2 given at a dose of 600,000 IU/kg i.v. every 8 hr for up to 14 doses each cycle; 2nd cycle planned 16 days after cycle 1 but may be delayed up to one week to allow toxicity to resolve | SBRT + IL-2 induced more OR with a higher DCR compared to IL-2 monotherapy in MM; IL-2 monotherapy resulted in a significantly higher ORR than anticipated^29^. |
| 112 | NCT00553618 | IL-2 | Dacarbazine | University of Louisville | Metastatic Melanoma | II | s.c. dose of 12 million units on days 1-4 for each of the six months of therapy | Active, not recruiting |
| 113 | NCT01659151 | IL-2 | TIL; Vemurafenib; Fludarabine and cyclophosphamide | H. Lee Moffitt Cancer Center and Research Institute | Metastatic Melanoma | II | HD IL-2 i.v. | BRAF-targeted therapy sensitized resistant melanoma cells to cytotoxic T lymphocytes; significant increase of mannose-6-phosphate receptor (M6PR) expression on tumors during vemurafenib treatment^30^. |
| 114 | NCT03374839 | IL-2 | TIL + Nivolumab | Nantes University Hospital | Melanoma | I, II | after TIL, s.c. injections of Proleukin® (IL-2) at a concentration of 6 million IU/day for 5 days | Recruiting |
| 115 | NCT04887259 | IL-2 | LAVA-051 - Bispecific Gamma-Delta T-Cell Engager (Gammabody)  (CD1d and the Vδ2-TCR) | Lava Therapeutics | CLL, MM, or AML | I, II | low dose IL-2 s.c. | LAVA-051 has been well tolerated early in dose escalation; a dose-dependent increase in LAVA-051 receptor occupancy of the Vγ9Vδ2-T cell receptor^30^. |
| 116 | NCT04687657 | IL-2 | umbilical cord blood | First Affiliated Hospital Xi'an Jiaotong University | Acute Myeloid Leukemia | I | If less than 20%, the NK cells are activated by s.c. injection of rhIL-2 on the second day after umbilical blood infusion | Recruiting |
| 117 | NCT04155710 | IL-2 | IOV-2001 - Autologous PBL; Fludarabine and cyclophosphamide | Iovance Biotherapeutics | CLL, SLL | I, II | Low dose IL-2: 6 doses of s.c. low dose IL-2 (9 MIU every 8-12 hr), or HD IL-2: 6 doses of i.v. HD-IL-2 (600,000 IU/kg Q8-12H, will follow the infusion of IOV-2001 | Recruiting |
| 118 | NCT05754684 | IL-2 | NK cells, anti-GD2 antibody Dinutuximab beta, GM-CSF and  retinoid X receptor gamma (RXRg) agonist spironolactone | Hong Kong Children's Hospital | Neuroblastoma | II | IL-2 s.c. alternate day for 6 doses | Recruiting |
| 119 | NCT04023071 | IL-2 | FT516 - allogeneic, off-the-shelf NK cell; Rituximab; Obinutuzumab; Fludarabine + cyclophosphamide, Bendamustine | Fate Therapeutics | acute myeloid leukemia (AML), B cell lymphoma | I |  | Administration of up to 6 doses of FT516 cells, including up to 300 million cells/dose, appeared to be safe and tolerable; three of 4 patients achieved OR (2 complete responses [CRs] and 1 partial response)^31^. |
| 120 | NCT03850691 | IL-2 | Nivo; Ipi | Masonic Cancer Center, University of Minnesota | Metastatic Melanoma | II | IL-2: 600,000 U/kg/dose given as a bolus infusion once every 8 to 12 hr over 5 days or until no longer tolerated (to a maximum of 10 doses). Days 1-5 and Days 15-19 (all patients) | Active, not recruiting |
| 121 | NCT01857934 | IL-2 | anti-disialoganglioside (anti-GD2) mAb hu14.18K322A with induction chemotherapy; allogeneic NK cells, G(M)-CSF | St. Jude Children's Research Hospital | Advanced Stage Neuroblastoma | II | IL-2: Given by continuous infusion during MRD maintenance, and SQ during induction | Adding hu14.18K322A to induction chemotherapy improved early OR, significantly reduced tumor volumes in most patients, improved end-of-induction response rates, and yielded an encouraging 3-year EFS^32^. |
| 122 | NCT03166397 | IL-2 | TIL; Nivo; Ipi; Fludarabine + cyclophosphamide | Sheba Medical Center | Metastatic Melanoma | II | IL-2: Bolus HD (720,000 IU/kg) IL-2 will be administered to each patient every 8 hr, to tolerance | Bone marrow depletion and recovery were equally affected by 120Cy/125Flu and 60Cy/125Flu preconditioning; however, toxicity and consequently duration of hospitalization were significantly lower in the 60Cy/125Flu cohort^33^. |
| 123 | NCT01586403 | IL-2 | autologous T cell receptor transduced T cells | Loyola University | Metastatic Melanoma | I | low dose IL-2 | In two of the three patients, adoptive transfer of tyrosinase-reactive TCR-transduced T cells into MM patients had clinical and/or biological activity without serious adverse events^34^. |
| 124 | NCT01704716 | IL-2 | Chemotherapy, surgery, radiation, immunotherapy | St. Anna Kinderkrebsforschung | High Risk Neuroblastoma | III |  | Genetic alterations of ALK (clonal mutations and amplifications) in HR-NB are independent predictors of poorer survival^35^. |
| 125 | NCT05155033 | IL-2 | Pembro | National Cancer Institute (NCI) | Metastatic Melanoma and Renal Cell Carcinoma | II | IL-2: 600,000 IU/kg i.v. bolus every 8 hr continuing for up to 4 days (maximum 10 doses)] starting on Day 1 of cycles 1 and 2 during Course 1 | Recruiting |
| 126 | NCT02870244 | IL-2 | Autologous bulk TIL 13831 TCR transduced T cells | Loyola University | Melanoma | I | low dose IL-2 | Culturing gene-modified T cells in the presence of histone deacetylase (HDAC) inhibitors maintained transgene expression and functional TCR-transduced T cell responses to tumor^36^. |
| 127 | NCT02027935 | IL-2 | Autologous CD8+ Melanoma Specific T Cells; Ipi; cyclophosphamide | M.D. Anderson Cancer Center | Metastatic Melanoma | II | aldesleukin s.c. twice daily (BID) on days 0-13 | Active, not recruiting |
| 128 | NCT04139057 | IL-2 | EBV-specific TCR-T cell with anti-PD1 auto-secreted element | Xinqiao Hospital of Chongqing | EBV-Positive NHSCC | I, II |  | Acceptable tolerance profile; 2 patients had PR (33.3%); 3 patients (50%) achieved stable disease; levels of TCR-T cells in peripheral blood peak between 3- and 14-days post-infusion, with a maximum duration of 180 days^37^. |
| 129 | NCT03997474 | IL-2 | ATL001: autologous clonal neoantigen reactive T cells; Nivo; lymphodepletion | Achilles Therapeutics UK Limited | Melanoma | I, II | low dose IL-2 | The early safety, tolerability, and durable clinical benefit in heavily pre-treated patients^38^. |
| 130 | NCT00026312 | IL-2 | isotretinoin with dinutuximab, and sargramostim; stem cell transplant | National Cancer Institute (NCI) | neuroblastoma | III | aldesleukin i.v. continuously on days 0-3 and 7-10 during courses 2 and 4. Immunotherapy repeats every 28 days for 5 courses | Immunotherapy with ch14.18, GM-CSF, and IL-2 was associated with a significantly improved outcome as compared with standard therapy in patients with high-risk neuroblastoma^39^. |

(i.v. – intravenous; i.p. – intraperitoneal; s.c. – subcutaneous; MTD – maximum tolerated dose; RoA - route of administration)

**Bibliography for Table S2**

1. Chatzkel JA, Swank J, Ludlow S, Lombardi K, Croft C, Artigas Y, Rodriguez Y, Terraciano T, Hart S, Rembisz J, Johnson E, Schell MJ, Yao J, Zhang J, Fishman MN. Overall responses with coordinated pembrolizumab and high dose IL-2 (5-in-a-row schedule) for therapy of metastatic clear cell renal cancer: A single center, single arm trial. J Clin Oncol 2019;37(7_suppl):657-657. doi: 10.1200/JCO.2019.37.7_suppl.657
2. de Bono J, Champiat S, Danlos F-X, Wermke M, Kunzmann V, De Gassart A, Valentin E, Iche M, Mairesse M, Brune P, Lemmens K, Marabelle A, Olive D, Frohna P. Abstract CT179: First-in-human study of ICT01, an anti-BTN3A activating monoclonal antibody in combination with low dose IL-2 in patients with advanced solid tumors (EVICTION-2 study). Cancer Res 2023;83(8_Supplement):CT179. doi: 10.1158/1538-7445.AM2023-CT179
3. Xing P, Yang J, Xu M, Wang J, Han D, Kong Y, Zhang J, Zhang L. PD-1 inhibitors combined with radiotherapy and GM-CSF, sequentially followed by IL-2 (PRaG 2.0) regimen in advanced refractory solid tumors: A prospective, multicenter, single-arm clinical trial. J Clin Oncol 2023;41(16_suppl):2603-2603. doi: 10.1200/JCO.2023.41.16_suppl.2603
4. Xu M, Chen R, Xing P, Kong Y, Zhao X, Zhang J, Cai S, Zhang L. A multicenter, phase II trial of RC48-ADC combined with radiotherapy, PD-1/PD-L1 inhibitor, GM-CSF, and sequential IL-2 (PRaG3.0 regimen) for salvage therapy in patients with HER2-expressing advanced solid tumors. J Clin Oncol. 2023;41(16_suppl):e14614. doi: 10.1200/JCO.2023.41.16_suppl.e1461
5. Goff SL, Dudley M, Citrin DE, Somerville R, Wunderlich JR, Danforth DN, Zlott DA, Yang JC, Sherry RM, Kammula US, Klebanoff C, Hughes MS, Restifo NP, Kwong ML, Ilyas S, Klemen N, Payabyab E, Steinberg SM, White DE, Rosenberg SA. A randomized, prospective evaluation comparing the intensity of lymphodepletion prior to adoptive transfer of tumor-infiltrating lymphocytes for patients with metastatic melanoma. J Clin Oncol. 2016;34(15_suppl):3006. doi: 10.1200/JCO.2016.66.7220
6. Nowicki TS, Berent-Maoz B, Cheung-Lau G, Huang RR, Wang X, Tsoi J, Kaplan-Lefko P, Cabrera P, Tran J, Pang J, Macabali M, Garcilazo IP, Carretero IB, Kalbasi A, Cochran AJ, Grasso CS, Hu-Lieskovan S, Chmielowski B, Comin-Anduix B, Singh A, Ribas A. A Pilot Trial of the Combination of Transgenic NY-ESO-1-reactive Adoptive Cellular Therapy with Dendritic Cell Vaccination with or without Ipilimumab. Clin Cancer Res. 2019 Apr 1;25(7):2096-2108. doi: 10.1158/1078-0432.CCR-18-3496
7. Creelan BC, Wang C, Teer JK, Toloza EM, Yao J, Kim S, Landin AM, Mullinax JE, Saller JJ, Saltos AN, Noyes DR, Montoya LB, Curry W, Pilon-Thomas SA, Chiappori AA, Tanvetyanon T, Kaye FJ, Thompson ZJ, Yoder SJ, Fang B, Koomen JM, Sarnaik AA, Chen DT, Conejo-Garcia JR, Haura EB, Antonia SJ. Tumor-infiltrating lymphocyte treatment for anti-PD-1-resistant metastatic lung cancer: a phase 1 trial. Nat Med. 2021 Aug;27(8):1410-1418. doi: 10.1038/s41591-021-01462-y
8. Pili R, Quinn DI, Hammers HJ, Monk P, George S, Dorff TB, Olencki T, Shen L, Hutson A, Piekarz R, Carducci MA. Immunomodulation by HDAC inhibition: Results from a phase II study with entinostat and high-dose Interleukin 2 in renal cell carcinoma patients (CTEP#7870). J Clin Oncol. 2016;34(2_suppl):500-500. doi: 10.1200/JCO.2016.34.15_suppl.4560
9. Seitter SJ, Sherry RM, Yang JC, Robbins PF, Shindorf ML, Copeland AR, McGowan CT, Epstein M, Shelton TE, Langhan MM, Franco Z, Danforth DN, White DE, Rosenberg SA, Goff SL. Impact of Prior Treatment on the Efficacy of Adoptive Transfer of Tumor-Infiltrating Lymphocytes in Patients with Metastatic Melanoma. Clin Cancer Res. 2021;27(19):5289–5298. doi: 10.1158/1078-0432.CCR-21-1171
10. Amaria RN, Vining DJ, Kopetz S, Overman MJ, Javle MM, Antonoff M, Tzeng CD, Wolff RA, Pant S, Lito K, Rangel KM, Wilson L, Fellman BM, Haymaker CL, Yuan Y, Forget M-A, Hwu P, Bernatchez C, Jazaeri AA. Efficacy and safety of autologous expanded tumor infiltrating lymphocytes (TILs) in multiple solid tumors. J Clin Oncol. 2022;40(16_suppl):2536-2536. doi: 10.1200/JCO.2022.40.16_suppl.2536
11. Liu Y, Fang W, Han Z, Chen K, Chen Y, Yu J, Liu Y, Ma L, Shi Z, Sun J, Cui J, Wang P. Harnessing the power of tumor-infiltrating lymphocytes: A first-in-human study of GT201 as monotherapy in advanced solid tumors. J Clin Oncol. 2023;41(16_suppl):2551-2551. doi: 10.1200/JCO.2023.41.16_suppl.2551
12. Sarnaik AA, Hamid O, Khushalani NI, Lewis KD, Medina T, Kluger HM, Thomas SS, Domingo-Musibay E, Pavlick AC, Whitman ED, Martin-Algarra S, Corrie P, Curti BD, Oláh J, Lutzky J, Weber JS, Larkin JMG, Shi W, Takamura T, Jagasia M, Qin H, Wu X, Chartier C, Graf Finckenstein F, Fardis M, Kirkwood JM, Chesney JA. Lifileucel, a Tumor-Infiltrating Lymphocyte Therapy, in Metastatic Melanoma. J Clin Oncol. 2021 Aug 20;39(24):2656-2666. doi: 10.1200/JCO.21.00612. Erratum in: J Clin Oncol. 2021 Sep 10;39(26):2972.
13. ClinicalTrials.gov. Universal Granulocyte Macrophage-colony Stimulating Factor (GM-CSF)-Producing and GM.CD40L for Autologous Tumor Vaccine in Mantle Cell Lymphoma. ClinicalTrials.gov Identifier: NCT00101101. <https://clinicaltrials.gov/ct2/show/results/NCT00101101>
14. Immatics. Immatics Presents Data Update on Dose Escalation from Ongoing ACTengine® Cell Therapy Programs. 17 March 2021. <https://www.globenewswire.com/fr/news-release/2021/03/17/2194401/0/en/Immatics-Presents-Data-Update-on-Dose-Escalation-from-Ongoing-ACTengine-Cell-Therapy-Programs.html>
15. Jazaeri AA, Zsiros E, Amaria RN, Artz AS, Edwards RP, Wenham RM, Slomovitz BM, Walther A, Thomas SS, Chesney JA, Morris R, Matsuo K, Gaillard S, Rose PG, Donas JG, Tromp JM, Tavakkoli F, Li H, Fardis M, Monk BJ. Safety and efficacy of adoptive cell transfer using autologous tumor infiltrating lymphocytes (LN-145) for treatment of recurrent, metastatic, or persistent cervical carcinoma. J Clin Oncol. 2019;37(15_suppl):2538-2538. doi: 10.1200/JCO.2019.37.15_suppl.2538
16. Rohaan MW, Borch TH, van den Berg JH, Met Ö, Kessels R, Geukes Foppen MH, Stoltenborg Granhøj J, Nuijen B, Nijenhuis C, Jedema I, van Zon M, Scheij S, Beijnen JH, Hansen M, Voermans C, Noringriis IM, Monberg TJ, Holmstroem RB, Wever LDV, van Dijk M, Grijpink-Ongering LG, Valkenet LHM, Torres Acosta A, Karger M, Borgers JSW, Ten Ham RMT, Retèl VP, van Harten WH, Lalezari F, van Tinteren H, van der Veldt AAM, Hospers GAP, Stevense-den Boer MAM, Suijkerbuijk KPM, Aarts MJB, Piersma D, van den Eertwegh AJM, de Groot JB, Vreugdenhil G, Kapiteijn E, Boers-Sonderen MJ, Fiets WE, van den Berkmortel FWPJ, Ellebaek E, Hölmich LR, van Akkooi ACJ, van Houdt WJ, Wouters MWJM, van Thienen JV, Blank CU, Meerveld-Eggink A, Klobuch S, Wilgenhof S, Schumacher TN, Donia M, Svane IM, Haanen JBAG. Tumor-Infiltrating Lymphocyte Therapy or Ipilimumab in Advanced Melanoma. N Engl J Med. 2022 Dec 8;387(23):2113-2125. doi: 10.1056/NEJMoa2210233
17. Orcurto A, Chiffelle J, Ghisoni E, Barras D, Crespo I, Rodrigo BN, Ochoa de Olza M, Imbimbo M, Rusakiewicz S, Tissot S, Gannon PO, Dafni U, Zimmermann S, Kandalaft LE, Michielin O, Bassani-Sternberg M, Dangaj D, Trueb L, Harari A, Coukos G. In-depth immune and molecular profiling of melanoma patients receiving adoptive T-cell therapy reveals biomarkers of efficacy in ATATIL study. J Clin Oncol. 2021;39(15_suppl):2533-2533. doi: 10.1200/JCO.2021.39.15_suppl.2533
18. Mullinax J, Weber JS, Khushalani NI, Eroglu Z, Brohl AS, Markowitz J, Royster E, Richards A, Zager JS, Sondak VK, Mule' JJ, Pilon-Thomas S, Sarnaik A. Final report of a pilot trial combining ipilimumab and adoptive cell therapy. J Clin Oncol. 2017;35(7_suppl):147-147. doi: 10.1200/JCO.2017.35.7_suppl.147
19. Silk AW, Curti B, Bryan J, Saunders T, Shih W, Kane MP, Hannon P, Fountain C, Felcher J, Zloza A, Kaufman HL, Mehnert JM, McDermott DF. A phase Ib study of interleukin-2 plus pembrolizumab for patients with advanced melanoma. Front Oncol. 2023 Feb 9;13:1108341. doi: 10.3389/fonc.2023.1108341
20. Amaria RN, Haymaker C, Forget M-A, et al. TGF-β dominant negative receptor (TGF-DNRII) and NGFR-transduced tumor-infiltrating lymphocytes (TIL) and high dose interleukin-2 (IL-2) in patients (pts) with metastatic melanoma (MM). Presented at: 2017 World Congress of Melanoma; October 18-21, 2017; Brisbane, Australia. Presentation SMR04-1.
21. Phillips S, Singh S, Lizee G, Solis LM, Welsh JW, Bassett RL, Beal LG, Kim PY, Murthy R, Talukder A, Lai I, Yee C, Patel SP. A phase Ib study of endogenous T cell therapy using SLC45A2-specific CD8 T cells for patients with metastatic uveal melanoma. J Clin Oncol. 2023 41:16_suppl, 9588-9588. doi: 10.1200/JCO.2023.41.16_suppl.9588
22. Hall M, Branthoover H, Innamarato P, et al. An investigation into the role of CD4+ tumor-infiltrating lymphocytes (TIL) in metastatic melanoma patients with a complete response to adoptive cell therapy. J ImmunoTherapy Cancer. 2021;9. doi: 10.1136/jitc-2021-SITC2021.384
23. Shapiro RM, Birch GC, Hu G, Vergara Cadavid J, Nikiforow S, Baginska J, Ali AK, Tarannum M, Sheffer M, Abdulhamid YZ, Rambaldi B, Arihara Y, Reynolds C, Halpern MS, Rodig SJ, Cullen N, Wolff JO, Pfaff KL, Lane AA, Lindsley RC, Cutler CS, Antin JH, Ho VT, Koreth J, Gooptu M, Kim HT, Malmberg KJ, Wu CJ, Chen J, Soiffer RJ, Ritz J, Romee R. Expansion, persistence, and efficacy of donor memory-like NK cells infused for posttransplant relapse. J Clin Invest. 2022 Jun 1;132(11):e154334. doi: 10.1172/JCI154334
24. Zhang X, Weng D, Pan Q, Liu J, Han Z, Peng R, Xu B, Wen X, Cen H, Yan C, Tan M, Zeng L, Lu S, Ou Y, Gong H, Lau JYN, Li Y, Fan Z. Phase I clinical trial to assess safety, pharmacokinetics (PK), pharmacodynamics (PD), and efficacy of NY-ESO-1–specific TCR T-cells (TAEST16001) in HLA-A*02:01 patients with advanced soft tissue sarcoma. J Clin Oncol. 2022 40:16_suppl, 11502-11502. doi: 10.1200/JCO.2022.40.16_suppl.11502
25. Lövgren T, Wolodarski M, Wickström S, Edbäck U, Wallin M, Martell E, Markland K, Blomberg P, Nyström M, Lundqvist A, Jacobsson H, Ullenhag G, Ljungman P, Hansson J, Masucci G, Tell R, Poschke I, Adamson L, Mattsson J, Kiessling R. Complete and long-lasting clinical responses in immune checkpoint inhibitor-resistant, metastasized melanoma treated with adoptive T cell transfer combined with DC vaccination. Oncoimmunology. 2020 Jul 11;9(1):1792058. doi: 10.1080/2162402X.2020.1792058
26. Saberian C, Amaria RN, Najjar AM, Radvanyi LG, Haymaker CL, Forget MA, Bassett RL, Faria SC, Glitza IC, Alvarez E, Parshottam S, Prieto V, Lizée G, Wong MK, McQuade JL, Diab A, Yee C, Tawbi HA, Patel S, Shpall EJ, Davies MA, Hwu P, Bernatchez C. Randomized phase II trial of lymphodepletion plus adoptive cell transfer of tumor-infiltrating lymphocytes, with or without dendritic cell vaccination, in patients with metastatic melanoma. J Immunother Cancer. 2021 May;9(5):e002449. doi: 10.1136/jitc-2021-002449
27. Nikanjam M, Mullen J, Yacoub C, et al. Combination high-dose interleukin-2 and nivolumab for programmed cell death-1 refractory metastatic melanoma: a case series. J Med Case Reports. 2022;16(1):337. doi: 10.1186/s13256-022-03536-y
28. Pili R, Quinn DI, Hauke RJ, Kuzel T, Han Y, Adra N, Logan TF. A randomized, open-label, phase II study of high-dose interleukin 2 vs high-dose interleukin 2 plus entinostat in renal cell carcinoma. J Clin Oncol. 2023 41:16_suppl, e16540-e16540. doi: 10.1200/JCO.2023.41.16_suppl.e16540
29. Curti B, Crittenden M, Seung SK, Fountain CB, Payne R, Chang S, Fleser J, Phillips K, Malkasian I, Dobrunick LB, Urba WJ. Randomized phase II study of stereotactic body radiotherapy and interleukin-2 versus interleukin-2 in patients with metastatic melanoma. J Immunother Cancer. 2020 May;8(1):e000773. doi: 10.1136/jitc-2020-000773
30. Broijl A, van de Donk NW, Bosch F, Mateos MV, Rodríguez-Otero P, Tucci A, Ghia P, Adang AE, Parren PWHI, Tuinhof I, Umarale S, Winograd B, van der Vliet HJ, Kater AP. Phase I dose escalation of LAVA-051, a novel bispecific gamma-delta T-cell engager (Gammabody), in relapsed/refractory hematological malignancies. J Clin Oncol. 2022 40:16_suppl, 2577-2577. doi: 10.1200/JCO.2022.40.16_suppl.2577
31. Strati P, Bachanova V, Goodman A, Pagel JM, Castro JE, Griffis K, Anderson M, Atwal SK, Bickers C, Fremgen D, Ly C, Cooley SA, Elstrom RL, Patel K. Preliminary results of a phase I trial of FT516, an off-the-shelf natural killer (NK) cell therapy derived from a clonal master induced pluripotent stem cell (iPSC) line expressing high-affinity, non-cleavable CD16 (hnCD16), in patients (pts) with relapsed/refractory (R/R) B-cell lymphoma (BCL). J Clin Oncol. 2021 39:15_suppl, 7541-7541. doi: 10.1200/JCO.2021.39.15_suppl.754
32. Furman WL, McCarville B, Shulkin BL, Davidoff A, Krasin M, Hsu CW, Pan H, Wu J, Brennan R, Bishop MW, Helmig S, Stewart E, Navid F, Triplett B, Santana V, Santiago T, Hank JA, Gillies SD, Yu A, Sondel PM, Leung WH, Pappo A, Federico SM. Improved Outcome in Children With Newly Diagnosed High-Risk Neuroblastoma Treated With Chemoimmunotherapy: Updated Results of a Phase II Study Using hu14.18K322A. J Clin Oncol. 2022 Feb 1;40(4):335-344. doi: 10.1200/JCO.21.01375
33. Nissani A, Lev-Ari S, Meirson T, Jacoby E, Asher N, Ben-Betzalel G, Itzhaki O, Shapira-Frommer R, Schachter J, Markel G, Besser MJ. Comparison of non-myeloablative lymphodepleting preconditioning regimens in patients undergoing adoptive T cell therapy. J Immunother Cancer. 2021 May;9(5):e001743. doi: 10.1136/jitc-2020-001743
34. Moore T, Wagner CR, Scurti GM, Hutchens KA, Godellas C, Clark AL, Kolawole EM, Hellman LM, Singh NK, Huyke FA, Wang SY, Calabrese KM, Embree HD, Orentas R, Shirai K, Dellacecca E, Garrett-Mayer E, Li M, Eby JM, Stiff PJ, Evavold BD, Baker BM, Le Poole IC, Dropulic B, Clark JI, Nishimura MI. Clinical and immunologic evaluation of three metastatic melanoma patients treated with autologous melanoma-reactive TCR-transduced T cells. Cancer Immunol Immunother. 2018 Feb;67(2):311-325. doi: 10.1007/s00262-017-2073-0. Epub 2017 Oct 20. Erratum in: Cancer Immunol Immunother. 2017 Dec 20.
35. Bellini A, Pötschger U, Bernard V, Lapouble E, Baulande S, Ambros PF, Auger N, Beiske K, Bernkopf M, Betts DR, Bhalshankar J, Bown N, de Preter K, Clément N, Combaret V, Font de Mora J, George SL, Jiménez I, Jeison M, Marques B, Martinsson T, Mazzocco K, Morini M, Mühlethaler-Mottet A, Noguera R, Pierron G, Rossing M, Taschner-Mandl S, Van Roy N, Vicha A, Chesler L, Balwierz W, Castel V, Elliott M, Kogner P, Laureys G, Luksch R, Malis J, Popovic-Beck M, Ash S, Delattre O, Valteau-Couanet D, Tweddle DA, Ladenstein R, Schleiermacher G. Frequency and Prognostic Impact of ALK Amplifications and Mutations in the European Neuroblastoma Study Group (SIOPEN) High-Risk Neuroblastoma Trial (HR-NBL1). J Clin Oncol. 2021 Oct 20;39(30):3377-3390. doi: 10.1200/JCO.21.00086
36. Tamson V. Moore, Gina M. Scurti, Matthew DeJong, Siao-Yi Wang et al. HDAC inhibition prevents transgene expression downregulation and loss-of-function in T cell-receptor-transduced T cells. Molecular Therapy Oncolytics. VOLUME 20, P352-363, MARCH 26, 2021. doi: 10.1016/j.omto.2021.01.014
37. Qingzhu Jia, Ling Peng, Gang Chen, Haiyang Wu, Dong Zeng, Tao Liu, Yunpeng Zhan, Si Li, Frank Su, Bo Zhu, and Qi-Jing Li. TCR-T cells armored with immune checkpoint blockade in EBV-positive nasopharyngeal carcinoma: The first-in-human phase 1/2 trial. Journal of Clinical Oncology. 2023 41:16_suppl, 6047-6047. doi: 10.3389/fimmu.2022.1079515
38. Achilles Therapeutics. Achilles Therapeutics Presents Encouraging Phase I/IIa Update on Clonal Neoantigen Reactive T Cells in Advanced NSCLC and Melanoma at ESMO IO Congress 2022. News release. December 6, 2022. https://finance.yahoo.com/news/achilles-therapeutics-presents-encouraging-phase-110000868.html
39. Yu AL, Gilman AL, Ozkaynak MF, London WB, Kreissman SG, Chen HX, Smith M, Anderson B, Villablanca JG, Matthay KK, Shimada H, Grupp SA, Seeger R, Reynolds CP, Buxton A, Reisfeld RA, Gillies SD, Cohn SL, Maris JM, Sondel PM; Children's Oncology Group. Anti-GD2 antibody with GM-CSF, interleukin-2, and isotretinoin for neuroblastoma. N Engl J Med. 2010 Sep 30;363(14):1324-34. doi: 10.1056/NEJMoa0911123
